# Genome-Wide Functional Profiling Reveals Genes Required for Tolerance to Benzene Metabolites in Yeast

**DOI:** 10.1371/journal.pone.0024205

**Published:** 2011-08-30

**Authors:** Matthew North, Vickram J. Tandon, Reuben Thomas, Alex Loguinov, Inna Gerlovina, Alan E. Hubbard, Luoping Zhang, Martyn T. Smith, Chris D. Vulpe

**Affiliations:** 1 Department of Nutritional Science and Toxicology, University of California, Berkeley, California, United States of America; 2 Division of Environmental Health Sciences, School of Public Health, University of California, Berkeley, California, United States of America; 3 Division of Biostatistics, School of Public Health, University of California, Berkeley, California, United States of America; Texas A&M University, United States of America

## Abstract

Benzene is a ubiquitous environmental contaminant and is widely used in industry. Exposure to benzene causes a number of serious health problems, including blood disorders and leukemia. Benzene undergoes complex metabolism in humans, making mechanistic determination of benzene toxicity difficult. We used a functional genomics approach to identify the genes that modulate the cellular toxicity of three of the phenolic metabolites of benzene, hydroquinone (HQ), catechol (CAT) and 1,2,4-benzenetriol (BT), in the model eukaryote *Saccharomyces cerevisiae*. Benzene metabolites generate oxidative and cytoskeletal stress, and tolerance requires correct regulation of iron homeostasis and the vacuolar ATPase. We have identified a conserved bZIP transcription factor, Yap3p, as important for a HQ-specific response pathway, as well as two genes that encode putative NAD(P)H:quinone oxidoreductases, *PST2* and *YCP4*. Many of the yeast genes identified have human orthologs that may modulate human benzene toxicity in a similar manner and could play a role in benzene exposure-related disease.

## Introduction

Benzene is a ubiquitous chemical in the environment, and is used in industry for the production of several important chemicals required for the synthesis of products that include plastics, resins, dyes and detergents. Benzene is also present in gasoline and cigarette smoke, resulting in extensive global population, as well as occupational, exposure. In the developing world, benzene is used as an industrial solvent, which leads to severe occupational exposures. The adverse health effects of benzene exposure were first described over one hundred years ago and it is now recognized as a cause of human blood disorders (hematotoxicity) and leukemia (reviewed in [1]).

Benzene undergoes extensive and complex metabolism in the human body, resulting in a number of well-characterized reactive metabolites [2]. This metabolism has made the elucidation of the mechanism of benzene carcinogenicity difficult, and it is speculated that a number of these metabolites work in concert to cause leukemia and other disorders [3], [4]. One class of benzene metabolites implicated as causative agents of benzene-associated disease, are the phenolic compounds hydroquinone (HQ), catechol (CAT), and 1,2,4-benzenetriol (BT). These compounds are all formed by the hydroxylation of phenol, which can be derived through a nonenzymatic rearrangement of benzene oxide, which is itself formed by the action of Cytochrome P450s on benzene in the liver and perhaps other organs [2]. HQ, CAT and BT can travel to the bone marrow and are oxidized both through autoxidation and by myeloperoxidase (MPO) to highly toxic quinones [4]. In humans, NAD(P)H:quinone oxidoreductase 1 (NQO1) catalyses the two-electron reduction of these quinones, detoxifying them to a less toxic hydroquinone. A polymorphism of NQO1, NQO1*2, has been linked to benzene toxicity and hematopoietic disorders [5]. NQO1*2 has a much shorter half-life than NQO1, leading to the suggestion that efficient reduction of the quinones produced from HQ, CAT and BT is an important defense against benzene toxicity. Indeed, other single-nucleotide polymorphisms (SNPs) in both *NQO1* and *MPO* have been shown to influence susceptibility to benzene hematotoxicity in individuals with low level occupational exposure [6]. Therefore, HQ, CAT, and BT may contribute significantly to benzene toxicity in the context of occupational exposure.

The phenolic metabolites of benzene, and especially their respective derived quinones, are highly reactive and known to non-specifically bind both DNA and protein, as well as to induce oxidative stress [7], [8], [9], [10]. This reactivity may in part explain the non-classical carcinogenesis of benzene. So-called classical carcinogens, such as benzo(a)pyrene, covalently bind DNA and are highly mutagenic, as measured by the Ames reversion test [11]. Although there is evidence that benzene and its metabolites can cause damage to DNA [12], [13], the levels detected are lower than those seen with known DNA-damaging carcinogens and benzene is an Ames negative compound [14]. Although the molecular modes of action of the phenolic metabolites of benzene in disease remain unclear, it has been postulated that they could produce DNA damage through a combination of damage to the mitotic spindle, inhibition of topoisomerase II and the formation of DNA strand breaks via reactive oxygen species (ROS) production [1], [15]. Additional characterization of the modes of action of these compounds is important to further our understanding of the pathology and susceptibility to benzene-associated disease.

The ascomycete yeast *Saccharomyces cerevisiae* is an ideal model organism for assessing gene function in mediating toxicity in response to environmental toxicants. Firstly, yeast shares many fundamental cellular processes with humans, which are extremely well characterized. Secondly, the functional tools available, particularly the genome-wide deletion strain collections, allow easy interrogation of the phenotype of every knockout under selective conditions [16]. Previous studies have revealed a low correlation between the upregulation of a specific gene's transcription and its requirement for growth under selective conditions [16], [17], and so growth studies are considered to be a more robust assay to identify genes required for the response to toxicant treatment. This approach has been used to gain insight into the genetic requirements for response to several compounds and nutritional states [16], [18], [19], [20], [21], and the human homologs or functional orthologs of genes identified by this approach have been implicated in altered sensitivity to the same compounds in human cells [22].

Here we report the findings of a genome-wide functional profiling screen of yeast to determine the genes required for optimal growth in response to treatment with HQ, CAT and BT. Our results indicate that a primary mode of toxicity of the phenolic metabolites of benzene is through the generation of oxidative stress. We also identified a requirement for the vacuolar ATPase and the correct regulation of iron homeostasis. Many of the yeast genes identified have human orthologs that could potentially modulate toxicity in a similar manner.

## Results

### Functional profiling of the yeast genome in response to hydroquinone, catechol and 1,2,4-benzenetriol reveals a dose- and time-dependent increase in differentially sensitive strains

The three phenolic benzene metabolites tested, hydroquinone (HQ), catechol (CAT), and 1,2,4-benzenetriol (BT), showed varying potencies of growth inhibition of the wild type yeast strain (Figures S1, S2 and S3); BT is the most inhibitory while HQ is the least. In order to study and compare the genetic requirements of yeast for growth in the presence of these compounds, we selected equitoxic concentrations that resulted in 20% growth inhibition of the wild type (IC_20_), as well as 50% and 25% of this IC_20_ (Table 1). We exposed pools of yeast homozygous diploid deletion mutants (n = 4607) for 5 generations and 15 generations of growth (5 g and 15 g), totaling six treatments per benzene metabolite, with three biological replicates of each (Table 1).

**Table 1:** Benzene metabolite treatments used in functional profiling experiments.

	Hydroquinone		Catechol		1,2,4-Benzenetriol	
Growth inhibitory concentration	5 g	15 g	5 g	15 g	5 g	15 g
25% IC_20_	1 mM	1 mM	0.55 mM	0.55 mM	87.5 µM	87.5 µM
50% IC_20_	2 mM	2 mM	1.1 mM	1.1 mM	175 µM	175 µM
IC_20_	4 mM	4 mM	2.2 mM	2.2 mM	350 µM	350 µM

Each treatment was performed in triplicate for a total of 18 pools of homozygous mutants per metabolite and compared with 12 rich media (YPD) controls in order to identify the strains that exhibit a significant change in growth.

**Figure 1:**
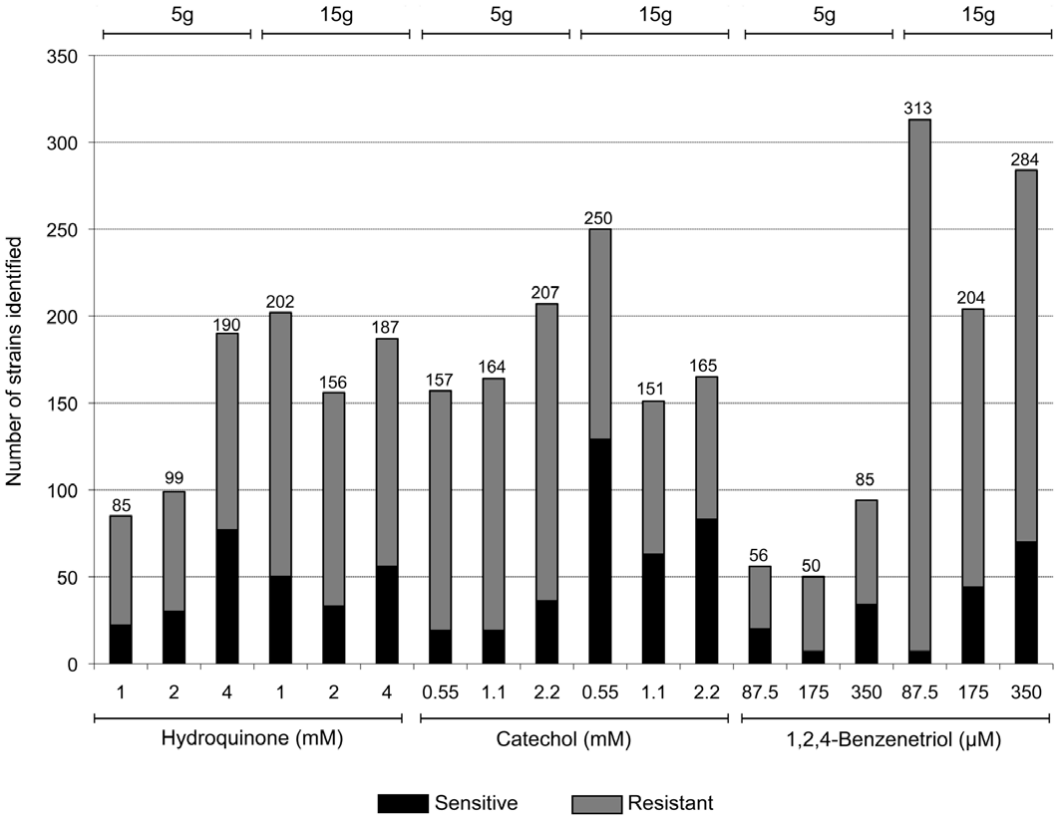
Numbers of sensitive and resistant deletion strains identified by differential sensitivity analysis (DSSA). Exposures were comprised of three equitoxic concentrations of HQ, CAT and BT, equivalent to 25% IC_20_, 50% IC_20_, and the IC_20_ for 5 g and 15 g of growth, resulting in a total of six treatments per benzene metabolite. The number of significant strains increased as the dose and number of generations increased.

**Figure 2:**
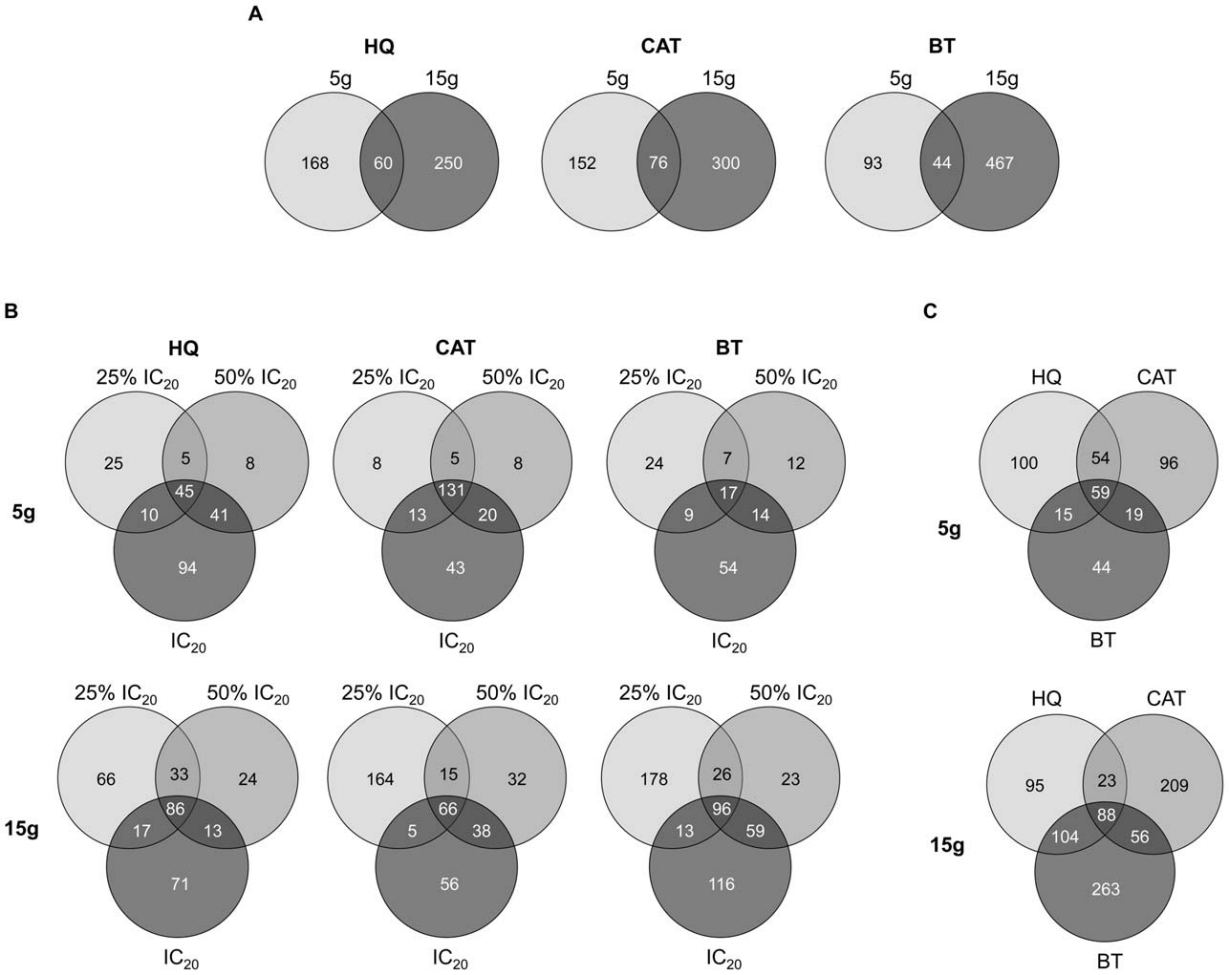
Venn diagrams showing the number of overlapping and distinct genes between treatments. **A** More genes were specific to 15 g with some overlap with 5 g. **B** Similarly, many genes at the highest HQ, CAT and BT concentrations were not found at the lower concentrations. **C** HQ, CAT and BT treatments identified different and overlapping sets of genes.

We used a differential strain sensitivity analysis (DSSA, described in Methods) to identify genes required by yeast for tolerance to these compounds. The number of identified strains (genes) correlated directly both to the dose and the number of generations of growth (Figure 1, Tables S1, S2 and S3), and show differing degrees of overlap (Figure 2). In total, 163 deletion strains were identified by DSSA as significantly sensitive to treatment with HQ (Table S1). Of these, 31 were significantly sensitive in three or more of the six treatment conditions (Table 2). 238 deletion strains were identified as sensitive to treatment with CAT (Table S2), with 33 in three or more treatment conditions (Table 3), and 122 as sensitive to BT (Table S3), with 12 sensitive in 3 or more conditions (Table 4).

**Table 2:** Genes with significant negative log_2_ ratios from DSSA identified in at least three of the six HQ treatments.

ORF	Standard name	5 g			15 g		
		25% IC_20_	50% IC_20_	IC_20_	25% IC_20_	50% IC_20_	IC_20_
		1 mM	2 mM	4 mM	1 mM	2 mM	4 mM
*YOL081W*	*IRA2*	−2.5	−3.3	−4.1	−4	−6.2	−2.6
*YOL085C*		−1.95	−2.1	−3	−4.35	−4.85	−3.3
*YHL009C*	*YAP3*	−2.3	−2.3	−2.9	−4.15	−3.6	
*YJL121C*	*RPE1*	−2.2	−2.4	−2.85	−3.3	−3	
*YDR032C*	*PST2*		−2.3	−3	−2.3	−3.4	−3.2
*YOL013C*	*HRD1*		−1.5	−2.1	−4.2	−3.8	−3.1
*YOL079W*			−2.4	−2.45	−3.55	−2.95	−3.35
*YOL025W*	*LAG2*		−1.7	−1.9	−3.5	−3.3	−2.55
*YJL120W*			−1.9	−2.25	−3.9	−3.8	
*YGR130C*		−2.1	−2.2	−2.4			
*YLR077W*	*FMP25*	−1.6	−1.5	−1.1			
*YKL147C*		−1.6	−1.4	−1.3			
*YCR068W*	*ATG15*	−1.6	−1.2	−1.6			
*YMR237W*	*BCH1*	−1.5	−1.3	−1.4			
*YOR084W*	*LPX1*	−1.3	−1.4	−1.3			
*YCR004C*	*YCP4*		−1.5	−1.5			−3.6
*YOR325W*			−1.3	−1.6			−2.2
*YDL020C*	*RPN4*			−1.6	−3.8	−3.4	
*YNL148C*	*ALF1*	−1.9		−2.5	−3.9		
*YGR139W*		−1.7	−1.5	−1.5			
*YHR030C*	*SLT2*			−1.6	−2.7		−3.3
*YKL040C*	*NFU1*			−1.4		−1.9	−2.1
*YIL162W*	*SUC2*				−3.8	−4.4	−4.1
*YMR022W*	*UBC7*				−2.4	−2.4	−2.2
*YBR035C*	*PDX3*				−2.4	−2.4	−2.3
*YPR074C*	*TKL1*				−2.3	−3.1	−3.8
*YDL225W*	*SHS1*				−1.8	−3.2	−2.9
*YDR112W*	*IRC2*				−4.1	−4.4	−4.3
*YEL056W*	*HAT2*				−3.6	−3.7	−3.8
*YDR457W*	*TOM1*				−3.1	−3.2	−2.2
*YPL138C*	*SPP1*				−2.8	−3.3	−3.2

The requirement of a gene for optimal growth in HQ is quantified with a fitness score, defined as the normalized log_2_ ratio of the deletion strain's growth. A total of 31 (19%) of the identified genes were important for fitness (i.e. had negative fitness scores) in at least three of the six HQ treatments. Most genes identified were significant in one or two treatments and after 15 g of growth. See Table S1 for the list of all identified genes.

**Table 3:** Genes with significant negative log_2_ ratios from DSSA identified in at least three of the six CAT treatments.

ORF	Standard name	5 g			15 g		
		25% IC_20_	50% IC_20_	IC_20_	25% IC_20_	50% IC_20_	IC_20_
		0.55 mM	1.1 mM	2.2 mM	0.55 mM	1.1 mM	2.2 mM
*YGR130C*		−2.3	−2.2	−2.3		−1.9	−1.8
*YPR076W*		−1.8	−1.8	−1.7	−2.9		
*YGR117C*		−2	−2	−1.9	−3.1		
*YKR046C*	*PET10*	−1.4	−1.4	−1.3	−4		
*YDL211C*		−1.2	−1.3	−1.1	−4		
*YAL002W*	*VPS8*			−2.3	−3.5	−3.6	−5
*YJL120W*				−1.5	−2.75	−2.95	−4
*YKL133C*				−1.2	2.8	2.6	2.6
*YJL121C*	*RPE1*			−1.1	−2.9	−2.6	−3.8
*YOR084W*	*LPX1*	−1.2	−1.1	−1.1			
*YPL230W*	*USV1*	−1.2	−1.2	−1.1			
*YHR060W*	*VMA22*	−1.4	−2.05	−2.45			
*YHR137W*	*ARO9*	−2	−2	−1.9			
*YDL182W*	*LYS20*	−1.7	−1.9	−1.8			
*YGL043W*	*DST1*	−1.6	−1.6	−1.4			
*YGL071W*	*AFT1*	−1.4	−2.65	−3.8			
*YKL119C*	*VPH2*	−2	−2.85	−2.4			
*YOR089C*	*VPS21*			−1.5		−2.45	−4.7
*YCL007C*		−2.1	−2.2	−2.7			
*YOR331C*		−2.1	−2.2	−2.1			
*YKR052C*	*MRS4*				−2.45	−4.35	−5.7
*YMR153W*	*NUP53*				−0.45	−2.2	−2
*YFR036W*	*CDC26*				−5.2	−4.95	−4.75
*YKR020W*	*VPS51*				−2.3	−2.4	−2.95
*YKL071W*					−1.6	−1.3	−1.3
*YKL123W*					−2.2	−1.6	−1.7
*YKL222C*					−2.2	−1.9	−2
*YGL094C*	*PAN2*				−2.1	−2.1	−2.2
*YGR110W*	*CLD1*				−1.9	−1.8	−1.9
*YDL236W*	*PHO13*				−1.8	−1.6	−1.7
*YKL051W*	*SFK1*				−1.8	−1.7	−1.8
*YKR033C*					*−1.8*	*−1.5*	*−1.6*
*YHL026C*					*−1.7*	*−1.4*	*−1.5*

The requirement of a gene for optimal growth in CAT is quantified with a fitness score, defined as the normalized log_2_ ratio of the deletion strain's growth. A total of 33 (14%) of the identified genes were important for fitness (i.e. had negative fitness scores) in at least three of the six CAT treatments. Most genes identified were significant in one or two treatments and after 15 g of growth. See Table S2 for the list of all identified genes.

**Table 4:** Genes with significant negative log_2_ ratios from DSSA identified in at least three of the six BT treatments.

ORF	Standard name	5 g			15 g		
		25% IC_20_	50% IC_20_	IC_20_	25% IC_20_	50% IC_20_	IC_20_
		87.5 µM	175 µM	350 µM	87.5 µM	175 µM	350 µM
*YBR292C*		−2.3	−1.5	−1.2			−1.5
*YJL120W*				−3.25	−2.95	−4.5	−4.6
*YGR130C*		−2.1	−2.4	−2.2			
*YJL121C*	*RPE1*			−2.6	−3.1		−4.2
*YHR206W*	*SKN7*			−2.45	−3.7	−4.7	
*YML028W*	*TSA1*			−2.2		−2.7	−3.3
*YGL148W*	*ARO2*			−1.3		−2.6	−3.05
*YPR074C*	*TKL1*			−1.3		−3.75	−3.9
*YLR380W*	*CSR1*			−1.1		−4.6	−2.8
*YBR035C*	*PDX3*				−2.2	−2.4	−2.6
*YIL162W*	*SUC2*				−2	−3.3	−3.7
*YFR036W*	*CDC26*				−3.1	−4.3	−4.4

The requirement of a gene for optimal growth in BT is quantified with a fitness score, defined as the normalized log_2_ ratio of the deletion strain's growth. A total of 12 (10%) of the identified genes were important for fitness (i.e. had negative fitness scores) in at least three of the six BT treatments. Most genes identified were significant in one or two treatments and after 15 g of growth. See Table S3 for the list of all identified genes.

### Functional category enrichment analysis indicates common requirements for tolerance of benzene metabolites

We analyzed the sensitive data sets for each metabolite (i.e. those with negative fitness scores from DSSA) for enrichment of biological attributes by identifying significantly overrepresented MIPS (Munich Information Center for Protein Sequences) Functional Classification categories (Table 5). A number of functional categories were shared by more than one metabolite including the oxidative stress response (MIPS Category 32.01.01) and the vacuole (42.25).

**Table 5:** Genes required for growth in the presence of HQ, CAT and BT, and their associated MIPS Functional Categories (genes were identified in at least one of the six treatments).

Hydroquinone				
MIPS category	p-value	Genes identified	k^a^	f^b^
Oxidative stress response	0.0022	*PST2, TRX2, SKN7, HYR1, OCA1, FRT1*	6	55
Stress response	0.006581	*CLN3, TPS2, SHO1, SLT2, CPR2, UTH1, UBC7, SIW14, RTS1, ASR1*	10	162
Chemoperception and response	0.007785	*RDS1, STB5, URE2*	3	17
Budding, cell polarity and filament formation	0.009887	*CLN3, RPN4, SHS1, SPT3, GIN4, SHO1, BUD27, SLT2, ARP1, RCY1, HSL1, NAP1, TPM1, LPX1, BEM4*	15	312

Catechol				
MIPS category	p-value	Genes identified	k^a^	f^b^
Vacuolar/lysosomal transport	2.91E-12	*VPS8, CCZ1, BSD2, STP22, PEP7, VPS3, CUP5, VMA8, VPS45, VAM7, VPS35, ATG27, VPS25, VMA5, AVT3, YPT52, VPS51, SNF7, VPS36, VPS9, PEP12, VPS21, VPS28, VPS30, ATG5, MRL1, VPS4*	27	153
Protein targeting, sorting and translocation	6.48E-07	*VPS8, CCZ1, BSD2, STP22, PEP7, VPS3, CUP5, VPS45, ERP5, VTH1, PEP8, VPS35, VPS25, YPT52, VPS51, SSA2, SNF7, VPS36, VPS9, NUP53, PEP12, VPS21, VPS17, TIM18, VPS28, VPS30, ATG5, VPS4*	28	281
Vacuole or lysosome	2.39E-06	*CCZ1, CUP5, VPS45, VAM7, TRX2, YHR138C, SFK1, VAM10, ATG5, VPS4*	10	44
MAPKKK cascade	0.002368	*PKH1, PTP3, SLT2, BCK1, SMK1*	5	27
Vesicular transport	0.003929	*TMN2, VPS45, VTH1, SSA2, YPT6, VPS9, VPS17, VPS30*	8	72
Lipid/fatty acid transport	0.004421	*DRS2, ITR1, SFH5, CSR1, PDR16, PDR5*	6	44
Vesicle formation	0.004489	*SYS1, ATG27, VPS51*	3	10
Intracellular transport vesicles	0.004489	*SYS1, ATG27, VPS51*	3	10
Endocytosis	0.004768	*FEN2, PKH1, CUP5, RCY1, YPT52, ENT4, VPS21*	7	59
Homeostasis of protons	0.006153	*CUP5, VMA8, VMA22, RAV1, VMA5, VPH2*	6	47
Osmosensing and response	0.007552	*PTP3, SHO1, VPS45, SLT2, YKL161C*	5	35
Oxygen and radical detoxification	0.007806	*PRX1, TRX2, TSA1*	3	12
Electron transport	0.009263	*GRX3, CUP5, VMA8, TRX2, OYE2, VMA5, SDH1, OYE3*	8	83

1,2,4-Benzenetriol				
MIPS category	p-value	Genes identified	k^a^	f^b^
Oxidative stress response	5.71E-05	*YBP1, TRX2, SKN7, HYR1, SRX1, YAP1, TSA1*	7	55
Vacuole or lysosome	0.001167	*DOA4, TRX2, SFK1, VPS33, VAM10*	5	44
Chemoperception and response	0.003383	*RDS1, STB5, IZH2*	3	17
Cellular export and secretion	0.00763	*ERP3, GCS1, PHO86, FPS1*	4	43
Lipid/fatty acid transport	0.008277	*FPS1, CSR1, NCR1, PXA1*	4	44
Homeostasis of metal ions	0.008941	*SIT1, AFT1, VMA5, FRE8, MAC1, IZH2*	6	98

a Number of genes of specific category required for tolerance to benzene metabolite.

b Number of genes of specific category by MIPS.

### Pathway enrichment, hierarchical clustering and network clustering analyses reveal shared tolerance requirements

**Figure 3:**
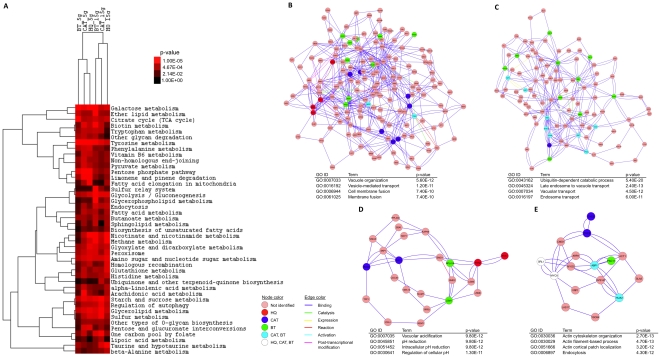
Pathway enrichment, hierarchical clustering and network clustering analyses of TAG4 microarray data identifying pathways and processes affected by benzene metabolite treatment. **A** Clustergram of KEGG pathways significantly affected by benzene metabolite treatment. **B–E** Clusters (sub-networks) identified by mapping fitness data to the STRING yeast interaction data set. Clusters were analyzed for enrichment of Gene Ontology (GO) Biological Processes within each sub-network. Cellular components required for metabolite tolerance identified from clusters included (parentheses indicate sub-network identification number – see Tables S5 and S6): **B** The vacuole (5 g #8). **C** Endosomes (15 g #27). **D** The vacuolar ATPase (15 g #39). **E** The actin cytoskeleton (15 g #34). In each panel, the complete cluster is visualized.

We performed two pathway analyses of the microarray data (described in Methods). In the first, we identified significantly enriched KEGG (Kyoto Encyclopedia of Genes and Genomes) pathways required for tolerance to treatment within the 5 g and 15 g data sets for each benzene metabolite (Table S4). Of the 99 annotated KEGG pathways available for *S. cerevisiae*, 43 had at least 60% of their genes represented in the data sets. These 43 were then clustered to determine shared tolerance pathways in both a compound- and generational-dependent context (Figure 3A). In the second analysis, we mapped data for all metabolites to the STRING database of *S. cerevisiae* protein-protein interactions and identified clusters (sub-networks) enriched for targets of any benzene metabolite (Figure 3B-E). Sub-networks from this analysis were then assessed for significant overrepresentation of Gene Ontology (GO) Biological Process categories. 19 sub-networks were identified at 5 g (Table S5), and 41 at 15 g (Table S6).

These analyses identified components required for response to all three metabolites and complement the DSSA and MIPS analyses conducted for each metabolite individually. Such pathway approaches would also allow the identification of essential genes likely to be involved in the processes overlooked in our study due to our lack of essential gene representation.

### Benzene metabolites induce oxidative stress

We found that multiple cellular components involved in the response to and protection from oxidative stress are required for growth in the presence of the three benzene metabolites. HQ, CAT and BT can all autoxidize to form quinones (HQ to 1,4-benzoquinone (1,4-BQ), CAT to 1,2-benzoquinone (1,2-BQ) and BT to 2-hydroxy-1,4-benzoquinone (2-OH-1,4-BQ)), and thus can undergo redox cycling by interacting with single electron acceptors such as O_2_, leading to the accumulation of reactive oxygen species (ROS) [23]. Phenolic compounds such as HQ, CAT and BT can also produce hydroxyl radicals through reaction with iron [24], and redox cycling. The oxidative stress response (32.01.01) was the most significantly overrepresented MIPS category in the HQ and BT data (Table 5), and the identification of oxygen and radical detoxification (32.07.07) in the CAT data indicates that all three compounds cause oxidative stress. A requirement for the oxidative stress response was not identified by pathway analysis as it is not a KEGG pathway and has few protein-protein interactions due to it being a transcriptional response.

**Figure 4:**
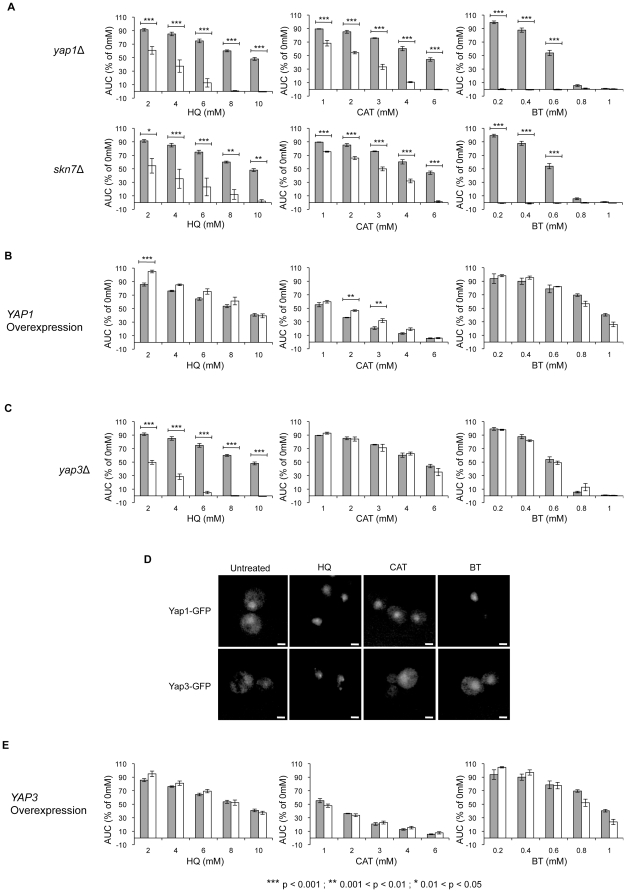
Confirmation of oxidative stress mutant sensitivity by growth curve analysis. The AUC was calculated for each strain after 24 h of exposure to the indicated doses of HQ, CAT and BT. The bars represent mean AUC as a percentage of the untreated for each strain with standard error of three replicates. Sensitivity was determined by comparison to the wild type strain (gray bars = wild type; white bars = indicated deletion/overexpression strain). **A** *yap1*Δ and *skn7*Δ are both sensitive to HQ, CAT and BT. **B** Overexpression of *YAP1* results in resistance to HQ and CAT, but not to BT. **C** *yap3*Δ is sensitive to only HQ. **D** Yap1-GFP is retained in the nucleus following treatment with HQ, CAT and BT, whereas Yap3-GFP is retained in the nucleus only following treatment with HQ. Cells were treated with the IC_20_ dose of each metabolite for 30 min. Scale bar = 2 µm. **E** Overexpression of *YAP3* does not affect tolerance of HQ, CAT or BT.

Yap1p and Skn7p are transcription factors that control two specialized oxidative stress regulons in yeast [25]. Both *yap1*Δ (BT- hereafter, the benzene metabolite treatments in which each strain was identified are indicated in parentheses following gene names) and *skn7*Δ (HQ, CAT, BT) were identified as being significantly sensitive to treatment with benzene metabolites, and subsequent analysis of the strains individually confirmed sensitivity (Figure 4A). Previous studies show that *yap1*Δ and *skn7*Δ both exhibit decreased resistance to oxidative stress, in part due to decreased induction of antioxidant defense pathways [25]. The strains were both least sensitive to CAT and most sensitive to BT. Several members of the Yap1p signal transduction pathway were also identified as sensitive: *HYR1* (HQ, BT) encodes a thiol peroxidase that senses intracellular hydroperoxide levels and transduces a redox signal to Yap1p [26], [27], while *YBP1* (BT) is required for the oxidation of cysteine residues of Yap1p [28]. In the absence of Ybp1p there is no nuclear accumulation of Yap1p in response to stress.

To provide further evidence of oxidative stress induction, we assessed the fitness of a strain overexpressing *YAP1* when exposed to HQ, CAT and BT (Figure 4B). *YAP1* overexpression has previously been shown to increase resistance to several oxidative stress-inducing compounds [29], [30], [31], and we found that it increased resistance of yeast cells to treatment with HQ and CAT (Figure 4B). The decrease in HQ and CAT toxicity observed following increased induction of antioxidant response genes suggests that both metabolites result in increased ROS. *YAP1* overexpression did not result in increased growth over WT following BT treatment (Figure 4B) even though BT appears to be the strongest oxidative stressor of the three (since *yap1*Δ was most sensitive to BT). We hypothesize that Yap1p may be saturated in WT cells following BT treatment i.e. that the downstream response is already maximal. The relatively small increase in tolerance to HQ and CAT can be explained by the observation that *YAP1* is only slightly overexpressed in this strain [32]. The commercially available *SKN7* overexpression strain was not assessed as Skn7p levels do not change [32] (the strain also has severe growth defects).

### Yap3p – hydroquinone-specific nuclear localization and sensitivity

The eight-member Yap family of basic leucine zipper (bZIP) transcription factors responds to cellular stressors [33]. In addition to the Yap1p oxidative stress response, Arr1p (Yap8p) responds to arsenicals [34], while Cad1p (Yap2p) responds to cadmium [31]. DSSA showed *yap3*Δ to be sensitive to HQ, which was confirmed by follow-up analysis (Figure 4C). Interestingly, *yap3*Δ was not found to be sensitive to CAT or BT (Figure 4C), indicating that Yap3p could be part of a specific stress response to HQ; the Yap3p response pathway has not been previously characterized. To further investigate the role of Yap3p, we determined the cellular localization of Yap3p following HQ, CAT and BT stress. As a control we studied Yap1p, which is retained in the nucleus following treatment with the oxidative stressor hydrogen peroxide [35]. We assessed the cellular distribution of Yap3-GFP and Yap1-GFP following 30 minutes treatment with the IC_20_ doses of HQ, CAT and BT. *yap1*Δ is sensitive to HQ, CAT and BT and we found Yap1-GFP was retained in nuclei following treatment with all three metabolites. In contrast, Yap3-GFP was retained only following treatment with HQ (Figure 4D), supporting a specific role for Yap3p in the transcriptional response to HQ.

To further assess the functional role of Yap3p in cellular tolerance of HQ, we assessed the fitness of a strain overexpressing *YAP3*. Unlike *YAP1* overexpression, *YAP3* overexpression did not result in increased tolerance to HQ, CAT or BT treatment (Figure 4E). The strain tested only overexpresses *YAP3* at a low level [32], thus it is possible that no resistance to HQ was seen due to the only minor increase in protein abundance. It could also be due to a combination of a small number of transcriptional targets coupled with already maximal induction of the Yap3p pathway in response to HQ treatment in WT cells.

### Oxidative stress protection is required for benzene metabolite tolerance

The thioredoxin and the glutaredoxin pathways carry out a significant amount of cellular oxidative stress protection. Several members of the cytoplasmic thioredoxin system were required for metabolite tolerance: Trx2p (HQ, CAT, BT), one of two yeast thioredoxin isoenzymes and cofactor of Tsa1p (CAT, BT) a thioredoxin peroxidase that acts as both a ribosome-associated and free cytoplasmic antioxidant. Deletion of *TSA1* results in hypersensitivity to oxidative stress [37]. *SRX1* (BT) encoding sulfiredoxin, a protein that contributes to oxidative stress resistance by reducing oxidant-formed cysteine-sulfinic acid groups in Tsa1p [38]. Finally, Csr1p (HQ, CAT, BT), which specifically interacts with thioredoxin peroxidase and may have a role in oxidative stress resistance [39].

Although oxidative stress was observed (as measured by the sensitivity of *yap1*Δ and the induction of the thioredoxin pathway), we found little requirement for the glutathione (glutaredoxin) pathway in yeast benezene metabolite tolerance, in contrast to data from both rodent and human epidemiological studies [15], [40], [41]. Given the contrasting data, we analyzed the growth of *gsh1*Δ and *glr1*Δ, involved in GSH synthesis and recycling, in the presence of HQ, CAT and BT (Figure S4). *gsh1*Δ showed no sensitivity to HQ and mild sensitivity to CAT and BT, while *glr1*Δ was not sensitive to CAT or BT, but was sensitive to HQ. However, at the IC_20_ dose of each, there was no significant requirement for GSH. Conversely, KEGG pathway analysis identified glutathione metabolism (KEGG pathway map00480) as significantly required for tolerance (Figure 3A). Together these data suggest that glutathione plays a less significant role than thioredoxin in yeast, and perhaps only responds to severe stress.

### Benzene metabolites induce endoplasmic reticulum stress

Deletion of proteasome transcription factor *RPN4* (HQ, BT), ubiquitin-conjugating enzyme *UBC7* (HQ), or ubiquitin-protein ligase *HRD1* (HQ), results in significant sensitivity to HQ. *hrd1*Δ is deficient in ER-associated protein degradation (ERAD) [42], suggesting the presence of damaged proteins and endoplasmic reticulum (ER) stress; HQ is known to bind proteins, producing adducts [9]. Kinase *SLT2* (HQ, CAT), involved in ER stress response, is also required for tolerance [43]. HQ, CAT and BT all reduce disulfide bridges by binding thiol groups in cysteines in the same manner as the reducing agent dithiothreitol (DTT), which is used experimentally to induce ER stress.

### Membrane lipids are cellular targets of benzene metabolites

Lipid/fatty acid transport (MIPS category 20.01.13) was identified in both the CAT and BT DSSA analysis (Table 5), and several pathways involved in lipid and fatty acid metabolism were identified by KEGG analysis (Figure 3A). Lipid peroxidation, the oxidative degradation of membrane lipids by free radical species, is caused by benzene metabolites[8] but is expected to be a secondary outcome of the oxidative stress that they cause; sensitivity is likely due to defects in new lipid metabolism, required to replace damaged membrane components. Genes identified include: *ISC1* (HQ), a mitochondrial phospholipase C that hydrolyses complex sphingolipids to produce ceramide, a major component lipid of cell membranes. *ISC1* has functional orthologs in both rats and humans, and *de novo* synthesis of ceramide is significantly inhibited by high doses of benzene in rats [44]. *NCR1* (CAT), a functional ortholog of the human Niemann Pick C1 (NPC1), involved in sphingolipid metabolism. *PXA1* (CAT), required for import of long chain fatty acids into peroxisomes; the peroxisome (map04146) was also identified by KEGG pathway analysis (Figure 3A) and could be required to prevent the fatty acids accumulating in the cytosol, as occurs when its function is impaired. Lastly, *DRS2* (BT), a flippase that maintains vesicle membrane lipid asymmetry, was identified; vesicle defects can result in increased sensitivity through a number of routes including reduced transport of damaged proteins to the vacuole.

### The vacuole is important for benzene metabolite tolerance

MIPS functional category analysis identified the vacuole/lysosome (42.25) as important for response to CAT and BT. This is supported by the identification of endocytosis by MIPS (20.09.18.09.01) and KEGG pathway analysis (map04144-Figure 3A) and by identification of the vacuole (Figure 3B), endosomes (Figure 3C), and vacuolar ATPase (Figure 3D) by protein-protein network analysis. Sensitive strains included *vam7*Δ (CAT) and *vam10*Δ (CAT, BT) with defects in vacuolar morphogenesis, and *vps3*Δ (CAT) and *vps8*Δ (CAT) lacking proteins of the CORVET endosomal tethering complex, required for biogenesis of the vacuole and endosomes. Similarly, components of the V-ATPase vacuolar proton pump (*vma5*Δ (HQ, BT), *vma8*Δ (CAT) and *cup5*Δ (CAT)) and proteins required for correct assembly of the V-ATPase (*vph2*Δ (CAT) and *vma22*Δ (HQ)) were identified as significantly sensitive following treatment with each of the metabolites tested. Sensitivity of vacuolar transport mutants could be indicative of vesicle trafficking defects, supported by MIPS analysis identification of protein targeting, sorting and translocation (14.04), and vesicular transport (20.09.07, 42.09) and formation (20.09.07.25) for CAT, and cellular export and secretion (20.09.16) for BT (Table 5). Disruption of trafficking could be a secondary effect of actin disruption by benzene metabolites (discussed below), as endosomes move along actin [45]. Lipid peroxidation caused by benzene metabolites could also lead to endosomal deficiency through damage to vesicular membranes.

### Iron homeostasis is required for benzene metabolite tolerance

Several iron homeostasis-related deletion strains that are sensitive to low iron were identified as sensitive to benzene metabolite treatment by DSSA and growth analysis (Figure S5). *AFT1* (HQ, CAT, BT) and *MAC1* (CAT, BT) encode transcription factors required for the transcriptional response to low iron and copper, respectively. *aft1*Δ cells are sensitive to low iron availability [46], and *mac1*Δ exhibits a secondary iron deficiency due to dependence of iron uptake on copper uptake, which is regulated by Mac1p [47]. *FRE8* (CAT, BT) encodes a protein with similarity to iron/copper reductases, and the absence of Fre8p increases sensitivity to low iron [47]. The requirement for V-ATPase subunit Cup5p (CAT) connects the V-ATPase and iron homeostasis, as it is important for both [48]. In contrast to higher eukaryotes, yeast lacks ferritin as a means of storing iron and so the majority of cellular iron is stored in the yeast vacuole. Disruption of V-ATPase function causes an increase in vacuolar pH, resulting in decreased iron solubility in the vacuole and decreased iron availability to the cell [49], [50]. The requirement of the V-ATPase in benzene metabolite tolerance may be to ensure adequate available iron. HQ and BT have been shown to chelate iron, reducing bioavailability [51], and CAT releases iron from ferritin by chelation [52].

### NADPH is required for benzene metabolite tolerance

The pentose phosphate pathway (map00030) was identified by KEGG analysis (Figure 3A), and two enzymes of this pathway (Rpe1p and Tkl1p) were identified as sensitive to all three benzene metabolites by DSSA, indicating a requirement for NADPH in tolerance. The pentose phosphate pathway is required for the efficient regeneration of cellular NADPH [53], which is needed for a number of detoxification enzymes in yeast, including the glutaredoxin and thioredoxin pathways. As *YJL120W* (HQ, CAT, BT) is antisense to *RPE1*, deletion of this dubious ORF is functionally equivalent to deletion of *RPE1*. Interestingly, KEGG analysis showed a more significant requirement for the pentose phosphate pathway at 5 g than 15 g, indicating that the cellular requirement for NADPH regeneration is greater in earlier generations. This may be due to rapid depletion of cellular NADPH upon initial exposure that can be corrected over time by upregulation of pathway genes.

### The cytoskeleton is a cellular target of benzene metabolites

The sensitivity of alpha-tubulin folding protein mutant *alf1*Δ (HQ, CAT) suggests that microtubules may be cellular targets of benzene metabolites. HQ can bind sulfhydyl groups and is known to inhibit polymerization of microtubules *in vitro* [54], [55], while BT has also been shown to inhibit tubulin polymerization [56]. In yeast, HQ has also been shown to induce aneuploidy, but not crossovers or gene conversions [57], consistent with this hypothesis. Protein-protein network analysis also identified the actin cytoskeleton as a cellular component affected by benzene metabolite treatment (Figure 3E). *ARP1* (HQ) was identified and encodes an actin-related protein of the dynactin complex, required for spindle orientation and nuclear migration. This indicates that benzene metabolites induce cytoskeletal stress, and agrees with observations that deficiencies in *HOG1* and *SWE1* (kinases that signal cytoskeletal stress and induce cell cycle arrest) abolish HQ-induced delay at the G2/M transition [57]. Actin stress could be a consequence of oxidative damage to cells by benzene metabolites; hydrogen peroxide-induced oxidative stress is known to cause actin reorganization in cultured human retinal pigment epithelial cells [58] and HQ also produces actin aggregates in the same cell type [59]. MIPS analysis provides additional evidence of cytoskeletal stress, with the identification of budding, cell polarity and filament formation (43.01.03.05) and MAPKKK cascade (30.01.05.01.03) as sensitive to HQ and CAT treatment, respectively (Table 5). Disruption of the actin cytoskeleton could also affect vesicle trafficking, as previously discussed.

### Pst2p and Ycp4p – NAD(P)H:quinone oxidoreductases?

**Figure 5:**
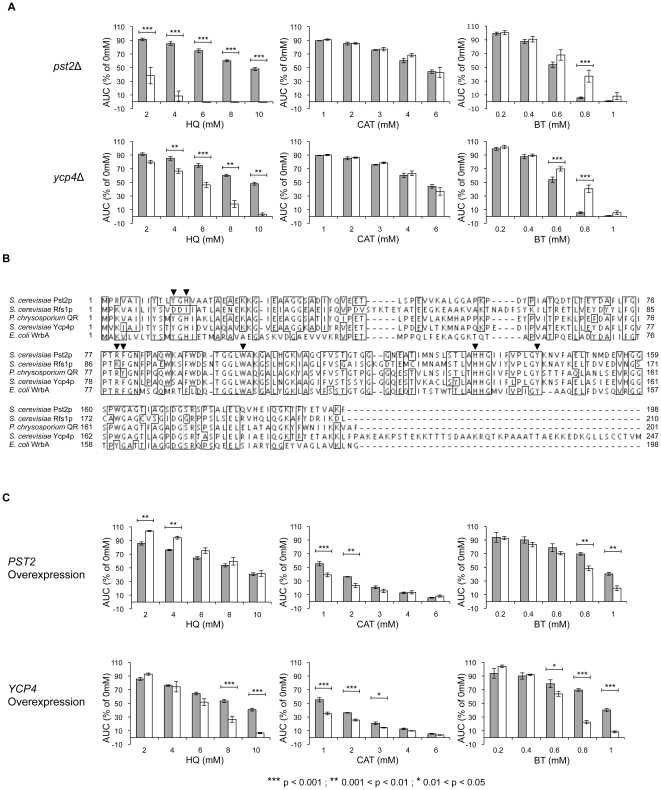
Growth curve analysis of putative quinone oxidoreductase mutants. The AUC was calculated for each strain after 24 h of exposure to the indicated doses of HQ, CAT and BT. The bars represent mean AUC as a percentage of the untreated for each strain with standard error of three replicates. Sensitivity was determined by comparison to the wild-type strain (gray bars = wild type; white bars = indicated deletion/overexpression strain). **A** *pst2*Δ and *ycp4*Δ are both sensitive to HQ, unaffected by CAT, and resistant to BT. **B** Protein sequence alignment (ClustalW2) of known and putative quinone oxidoreductases. Identical amino acids in at least 4/5 ORFs analyzed are boxed. Residues thought important for WrbA function are indicated by arrowheads. **C** Overexpression of *PST2* results in resistance to HQ, and sensitivity to CAT and BT. Overexpression of *YCP4* results in sensitivity to HQ, CAT and BT.

Both *pst2*Δ (HQ) and *ycp4*Δ (HQ) were identified by DSSA as sensitive to only HQ. Pst2p and Ycp4p are members of a family of three flavodoxin-like proteins (the third is Rfs1p), whose molecular function and cellular role are unknown. Pst2p and Ycp4p have both been detected in mitochondria in high-throughput studies, but may also be present in the cytoplasm. Pst2p is 67% identical to Ycp4p (100% coverage), and 53% identical to Rfs1p (98% coverage). Ycp4p is the largest of the three proteins (247 amino acids), and has a longer C-terminal region; Pst2p and Rfs1p are similar in length (Pst2p is 198 amino acids; Rfs1p is 210 amino acids). *PST2* is induced by oxidative stress in a Yap1p-dependent manner [25], and *YCP4* has two upstream AP-1-like sites, indicative of Yap1p-binding [60]. Sensitivity of both *pst2*Δ and *ycp4*Δ to HQ was confirmed by analysis of the individual deletion strains (Figure 5A). Neither mutant was sensitive to CAT, but both were resistant to BT (Figure 5A). Domain predictions using the protein sequences of Pst2p, Ycp4p and Rfs1p revealed a single, shared, predicted domain. The reference parent, 3b6iA, is annotated in the RCSB Protein Data Bank (http://www.rcsb.org/pdb) as one chain of WrbA [61], a bacterial flavodoxin-like NAD(P)H:quinone oxidoreductase from *E. coli* that is closely related to mammalian Nqo1 [23], [62]. NAD(P)H:quinone oxidoreductases catalyze the two-electron reduction of toxic quinones to their corresponding less toxic hydroquinone, using either NADH or NADPH as an electron donor, and with a flavin cofactor, either FMN or FAD [63]. Direct two-electron reduction avoids redox cycling and the resulting production of an extremely toxic and reactive semiquinone intermediate. Concordantly, polymorphisms of the human quinone oxidoreductase NQO1 associate with a higher incidence of benzene-associated disease [6], [64]. Protein sequence comparisons also support Pst2p, Ycp4p and Rfs1p as orthologs of WrbA (Figure 5B). Pst2p and Ycp4p are both 46% identical to WrbA (Pst2p = 95% coverage; Ycp4p = 80% coverage), and Rfs1p is 37% identical to WrbA (95% coverage). Ycp4p has previously been proposed as a structural homolog of WrbA [60], and Pst2p, Ycp4p and Rfs1p are also all orthologs of the previously characterized 1,4-benzoquinone oxidoreductase from the basidiomycete fungus *Phanerochaete chrysosporium* [65] (Figure 5B). Alignment of the five protein sequences shows extensive sequence conservation, particularly in regions identified in WrbA as important for protein function (Figure 5B), such as four key active site residues (Phe-80, Trp-98, His-133 and Tyr-143). Although Rfs1p has some conserved sites, there are several other residues important for WrbA function that are conserved in Pst2p and Ycp4 but not Rfs1p [61], [62], and *rfs1*Δ has no consequences for response to benzene metabolites (Figure S6). Thus, Rfs1p may not function as a quinone oxidoreductase.

The only characterized yeast NAD(P)H:quinone oxidoreductase is cytoplasmic Lot6p [66]. Lot6p is also a WrbA ortholog and is considered to be the yeast ortholog of human NQO1. Despite both being orthologous to WrbA, Pst2p shares only 31% identity with Lot6p (72% coverage). Lot6p is known to reduce 1,4-BQ *in vitro* [66], but *lot6*Δ was not identified as sensitive to either HQ or BT by DSSA. We assessed the growth of *lot6*Δ following benzene metabolite treatment and confirmed that it is not sensitive to either HQ or BT (Figure S6). Interestingly, *lot6*Δ was sensitive to CAT (Figure S6), suggesting that Lot6p can reduce 1,2-BQ *in vivo*; however, this reduction may not be efficient as there was no observed sensitivity of *lot6*Δ at a concentration close to the IC_20_.

To gain greater insight into the roles of these putative oxidoreductases in benzene metabolite tolerance, we assessed the fitness of yeast strains overexpressing both *PST2* and *YCP4* during exposure (Figure 5C). Overexpression of quinone oxidoreductases is known to modulate the toxicity of compounds; for example, NQO1 expression influences toxicity of the chemotherapeutic agent mitomycin C. Mitomycin C has a quinone ring that when reduced to a hydroquinone becomes a potent alkylating agent. Many tumor cells have endogenous overexpression of NQO1 [67], which explains the chemotherapeutic effectiveness of the drug. In cell culture, NQO1 overexpression also increases mitomycin C cytotoxicity [68].

Given these data, overexpression of *PST2* and *YCP4* in yeast should reverse the sensitivities seen in the corresponding deletions following HQ treatment; both should become resistant due to increased reduction of the more toxic 1,4-BQ back to HQ. This is true for overexpression of *PST2* (Figure 5C), but not *YCP4*, which causes increased sensitivity to higher doses of HQ. A reversal of the BT resistance measured in *pst2*Δ and *ycp4*Δ was also observed when *PST2* and *YCP4* were overexpressed; both strains showed increased sensitivity (Figure 5C). Overexpression of both *PST2* and *YCP4* resulted in further increased sensitivity of both strains to CAT treatment, for unknown reasons. This unexpected increase could reflect strain background differences (in terms of the enzymatic defense against CAT) between the diploid deletion and haploid overexpression strains.

## Discussion

Benzene, a ubiquitous environmental contaminant that is widely used in industry, is an established human carcinogen. It undergoes extensive metabolism in humans and thus the molecular mechanisms underlying its toxicity remain elusive despite extensive study. To gain insight into these mechanisms we have performed genome-wide functional profiling of yeast, in order to identify non-essential genes required for tolerance to treatment with three phenolic benzene metabolites, hydroquinone (HQ), catechol (CAT) and 1,2,4-benzenetriol (BT). Assessing these metabolites individually has identified overlapping and synergistic toxicities, consistent with previously proposed mechanisms of carcinogenesis of benzene in which multiple metabolites act in concert [3], [4]. Comparison of the genes associated with sensitivity to these compounds suggests a mechanistic basis for toxicity and has identified conserved toxicity genes in humans (Table 6).

**Table 6:** Selected yeast genes required for benzene metabolite tolerance and their orthologous human genes.

Yeast gene^a^	Human gene ortholog	Human protein
*ALF1*	*TBCB*	Tubulin-folding cofactor B
*ARP1*	*ACTR1B*	Beta-centractin
*CUP5*	*ATP6V0C*	V-type proton ATPase 16kDa proteolipid subunit
*DRS2*	*ATP8B1*	Probable phospholipid-transporting ATPase 1C
*FRE8*	*DUOX2*	Dual oxidase 2
*HYR1*	*GPX3*	Glutathione peroxidase 3
*HRD1*	*SYVN1*	E3 ubiquitin-protein ligase synoviolin
*ISC1*	*SMPD2*	Sphingomyelin phosphodiesterase 2
*MRS4*	*SLC25A37*	Mitoferrin-1
*NCR1*	*NPC1*	Niemann-Pick C1 protein
*NFU1*	*NFU1*	NFU1 iron-sulfur cluster scaffold homolog, mitochondrial
*RPE1*	*RPE*	Ribulose-phosphate 3-epimerase
*SRX1*	*SRXN1*	Sulfiredoxin-1
*TKL1*	*TKT*	Transketolase
*TRX2*	*TXN*	Thioredoxin
*TSA1*	*PRDX1*	Peroxiredoxin-1
*VMA5*	*ATP6V1C1*	V-type proton ATPase subunit C 1

a Deletion of any of these genes increased the sensitivity of the mutant strain to HQ and/or CAT and/or BT (listed in alphabetical order).

**Figure 6:**
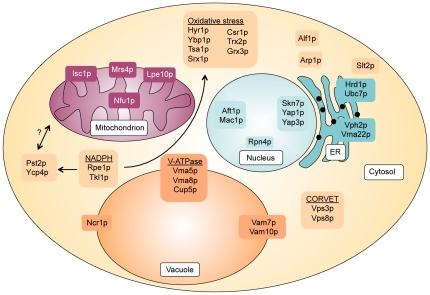
Proteins identified as being required for tolerance to benzene metabolites.

We determined that yeast cells are more resistant to HQ, CAT and BT than human cells, based on comparison of yeast IC_20_ doses with treatment doses used in studies with human cells in culture [69], [70], [71]. This increased resistance could be due to the absence of peroxidase enzymes in yeast (such as MPO in humans), which rapidly catalyze the oxidation of HQ, CAT and BT to more toxic quinone species. We identified both distinct and overlapping cellular processes and proteins between metabolites as important for tolerance, including the oxidative stress response, lipid/fatty acid transport, and the pentose phosphate pathway (Figure 6). Data also indicate that metabolites (particularly HQ) damage proteins, leading to both cytoskeletal and ER stress, and a requirement for ER-associated protein degradation. The vacuole, particularly the vacuolar ATPase, is important for tolerance. Benzene metabolites, especially CAT, appear to disrupt intracellular vesicle trafficking as an indirect result of lipid peroxidation caused by oxidative stress and/or cytoskeletal disruption. CAT may affect trafficking more than HQ and BT due to a bias in the species of ROS produced upon exposure, discussed below. These shared functional categories indicate that some molecular targets may be common to HQ, CAT and BT.

In agreement with observations from previous studies in other organisms [8], [72], [73], our results show that the primary mechanism of toxicity of these metabolites is induction of oxidative stress; we did not identify a requirement for the mitochondrial electron transport chain, suggesting that the level of hydrogen peroxide produced by HQ, CAT and BT does not significantly contribute to oxidative toxicity. The DSSA profiles identified in our study for HQ, CAT and BT are similar to that seen following cumene hydroperoxide treatment, an oxidant that is structurally similar to the phenolic metabolites of benzene [74], and requires the vacuole and vacuolar ATPase for tolerance. The profiles of HQ and BT from DSSA are more similar to each other than to that of CAT, perhaps reflecting differential ROS generation due to the difference between the *para* (1,4) position of the hydroxyl groups of HQ and BT and the *ortho* (1,2) hydroxyl groups of CAT. HQ, CAT and BT likely produce differing proportions of a wide range of ROS, which require multiple oxidative stress responses for tolerance. The lack of significant sensitivity of yeast glutathione pathway mutants to HQ, CAT and BT reflects either a difference in detoxification pathways between yeast and humans, or functional redundancy between glutathione pathway enzymes in yeast. The lack of significant sensitivity to decreased GSH levels suggest that the yeast thioredoxin system may be more important than the glutaredoxin system for benzene metabolite tolerance; previous studies have suggested it also has a more prominent role in hydrogen peroxide metabolism [75]. Based on these results, it may be prudent to reevaluate the role of the thioredoxin pathway in the human response to benzene metabolites.

Our data implicate the previously uncharacterized bZIP transcription factor Yap3p as being involved in a cellular response specific to HQ. Yap3p may respond to ER stress, as *yap3*Δ is sensitive to tunicamycin, a compound that causes ER stress through induction of the unfolded protein response [53]. This is consistent with the identification of more ER stress sensitive mutants in the HQ data compared to the CAT and BT data, and suggests that HQ causes more extensive damage to proteins than CAT or BT. *yap3*Δ was also identified in a similar screen as being sensitive to arsenic (As) treatment [21], suggesting that As and HQ (but not CAT or BT) share some cellular targets; analysis of transcription factor binding sequences has previously shown that Yap3p sites co-occur with those of Arr1p (Yap8p), the major arsenic response transcription factor [76]. Tubulin may be such a shared cellular target, as both HQ and As are able to bind to tubulin [21]. This could indicate that Yap3p is involved in the response to cytoskeletal stress, though our hypothesis is complicated by the fact that BT also inhibits microtubule polymerization [56] without *yap3*Δ cells being sensitive to it. Overexpression of *Candida albicans YAP3* (*FCR3*) in *S. cerevisiae* results in increased transcription of *PDR5*, an ABC transporter that confers resistance to a range of xenobiotic compounds [77], and Yap3p also interacts with Pdr5p [78]. *pdr5*Δ was not identified as significantly sensitive to HQ in this study (though it is sensitive to CAT) and is also not sensitive to As [21], indicating that although *PDR5* is a Yap3p target, it is not involved in the cellular response to HQ or As. High-throughput studies of the transcriptional targets of the total complement of yeast transcription factors are currently being undertaken by several groups [79], and these data will soon elucidate Yap3p targets and provide insight into the Yap3p stress response.

Several other processes important for benzene metabolite tolerance such as the vacuole, iron homeostasis, and lipid peroxidation, were likely identified due to the induction of oxidative stress by these compounds. Loss of the V-ATPase results in chronic oxidative stress [80] and sensitizes cells to exogenous oxidative stress; previous data show that vacuolar function is required for oxidative stress resistance [74]. The requirement of the V-ATPase may also be due to a need to maintain soluble (i.e. bioavailable) iron in the vacuole. The known decrease in available cellular iron caused through chelation by benzene metabolites [51] is potentiated by a disrupted V-ATPase [49], [50], leading to extremely low iron levels. HQ, CAT and BT can also cause iron deficiency through the disruption of iron-sulfur (Fe-S) clusters by ROS. Fe-S clusters are vital reactive centers of a large number of proteins and are vulnerable to attack and disruption by ROS. Loss of Fe^2+^ from Fe-S clusters can lead to further ROS production through Fenton chemistry [81], resulting in amplified disruption; increased synthesis and assembly of new Fe-S clusters then depletes available iron. Evidence to support the disruption of Fe-S clusters by benzene metabolites comes from the identification by DSSA of *nfu1*Δ (HQ). Nfu1p is a scaffold protein required for correct assembly of Fe-S clusters in the mitochondria [82]. Additionally, *MRS4* (CAT, BT) encodes a mitochondrial iron transporter required for efficient mobilization of iron into the mitochondria, and is known to be sensitive to low iron [47]; under conditions requiring the increased synthesis of Fe-S clusters, *mrs4*Δ cells are unable to transport sufficient iron into mitochondria. The identification of sulfur metabolism (map00920) by KEGG pathway analysis supports a requirement for increased Fe-S synthesis in response to HQ, CAT and BT treatment.

We identified Pst2p and Ycp4p as putative mitochondrial NAD(P)H:quinone oxidoreductases that are capable of reducing *para* (1,4) quinones (such as those produced through autoxidation of HQ and BT), but not *ortho* (1,2) quinones (e.g. 1,2-BQ from autoxidation of CAT). The absence of Pst2p or Ycp4p is therefore deleterious following HQ treatment due to increased levels of more toxic 1,4-BQ. The resistance of both *pst2*Δ and *ycp4*Δ to BT can be explained by the autoxidation rate of BT, which is higher than that of HQ or CAT [83]. HQ, CAT and BT all autoxidize by two successive one-electron oxidations, producing an extremely reactive semiquinone intermediate; these semiquinones are the most reactive and most toxic of the quinone species. Constant reduction of 2-OH-1,4-BQ back to BT results in elevated levels of transient semiquinones through repeat autoxidation of BT. Therefore, preventing reduction of 2-OH-1,4-BQ is beneficial to the cell as increased 2-OH-1,4-BQ is less harmful than increased transient semiquinone levels; concordantly, strains overexpressing *PST2* and *YCP4* have increased sensitivity to BT. The HQ resistance and BT sensitivity of the *PST2* overexpression strain is a reversal of the *pst2*Δ phenotype, and provides mechanistic support of the hypothesis that Pst2p and Ycp4p are quinone oxidoreductases. It is unclear why *YCP4* overexpression causes increased sensitivity to HQ, CAT and BT, but it could be due to unintended general cellular consequences; the C-terminal region of Ycp4p, which is not present in Pst2p, may aggregate at high abundance, causing cells to become sensitized to any stressor. The lack of sensitivity of *pst2*Δ and *ycp4*Δ to CAT (Figure 4A) can be explained if Pst2p and Ycp4p are only able to reduce *para* quinones. Based on homology to the bacterial oxidoreductase WrbA, we propose that they function by direct two-electron reduction of quinones with the same mechanism as human NQO1 and the characterized cytoplasmic yeast NAD(P)H:quinone oxidoreductase Lot6p. Lot6p is not required for tolerance to HQ or BT, despite Lot6p being able to reduce 1,4-BQ *in vitro*. *lot6*Δ is, however, moderately sensitive to CAT, indicating that Lot6p may be capable of reducing 1,2-BQ *in vivo*. HQ and BT may target the mitochondria more than CAT, as the CAT sensitivity profile has more representation of cytoplasmic targets, such as vesicular trafficking. We propose Pst2p and Ycp4p to be novel yeast orthologs of NQO1 that are required for HQ tolerance.

Both homologous recombination (map03440) and non-homologous end joining (map03450) DNA repair pathways were identified by KEGG analysis as associated with sensitivity to benzene metabolite treatment (Figure 3A), but the absence of strains identified by DSSA suggests genotoxicity is limited. However, DNA damage caused by these metabolites has been reported in many other studies and they have also been shown to bind DNA [9]. As our study shows very strong evidence for oxidative stress induction but only limited evidence of DNA damage, we suggest that a significant proportion of damage measured in other studies is an indirect effect resulting from reactive oxygen intermediates and/or topoisomerase inhibition [84], [85], [86], [87], [88], though we cannot rule out species differences in damage repair.

Oxidative DNA damage occurs in several ways: Hydrogen peroxide generated by CAT exposure increases the amount of 8-oxodG [85], while lipid peroxidation results in the production of malondialdehyde, which reacts with dA and dG in DNA. Lipid damage also produces etheno adducts in DNA that rearrange to form crosslinks between strands [89]. Yeast cells lacking the DNA helicase Sgs1p, required for the maintenance of genomic stability, have increased sensitivity to high doses of HQ [90], and RNAi knockdown of *WRN*, the human ortholog of *SGS1*, increases HQ-generated DNA damage [91].

Many of the genes identified in this study have human orthologs (Table 6). Only two (*PRDX1* and *TXN*) are currently associated with HQ in the Comparative Toxicogenomics Database (http://ctd.mdibl.org) and none are associated with CAT or BT. All could be novel targets or modulators of benzene toxicity in humans. Though oxidative stress appears to be the primary means through which these metabolites exert their toxicity, many secondary processes were also identified that could be informative in terms of human susceptibility. The deleterious health effects caused by benzene in humans are likely due to multiple modes of toxicity, induced by multiple metabolites, acting synergistically [4].

Benzene is thought to cause leukemia through metabolite-induced oxidative stress, via mitochondrial imbalance in hematopoietic cells [92]. The presence of phenolic benzene metabolites in bone marrow is likely to generate more oxidative stress than observed in yeast due to the presence of enzymes such as myeloperoxidase, which enhances the production of benzoquinones through direct oxidation [4]. There may be many as-yet unidentified SNPs in human oxidative stress response genes that could increase susceptibility to benzene toxicity and that should be studied.

## Materials and Methods

### Yeast strains and culture

Diploid yeast deletion strains used in parallel analysis pools and for individual strain analyses were of the BY4743 background (*MATa*/*MAT*α, *his3*Δ*1*/*his3*Δ*1*, *leu2*Δ*0*/*leu2*Δ*0*, *lys*Δ*0*/*LYS2*, *MET15*/*met15*Δ*0*, *ura3*ΔΔ*0*/*ura3*Δ*0*, Invitrogen Corporation, Carlsbad, CA). Yap1-GFP and Yap3-GFP strains were of the haploid BY4741 background (*MATa*, *his3*Δ*1*, *leu2*Δ*0*, *met15*Δ*0*, *ura3*Δ*0*, Invitrogen Corporation, Carlsbad, CA). Haploid yeast MORF (Movable ORF-[32]) strains for protein overexpression were of the Y258 background (*MATa*, *pep*4-3, *his*4-580, *ura*3-53, *leu*2-3,112, Thermo Fisher Scientific Open Biosystems, Huntsville, AL). Growth was conducted in liquid rich media (1% yeast extract, 2% peptone, 2% dextrose, YPD) for deletion strain pool growth and growth curve assays, and liquid synthetic complete media lacking uracil (SC-ura) using either 2% dextrose or 2% raffinose as a carbon source for pre-growths of overexpression strains for growth curve assays (detailed below). Liquid rich media for induction of protein overexpression for growth curve assays was liquid rich media containing both galactose and raffinose (1% yeast extract, 2% peptone, 2% galactose, 2% raffinose, YPGal+Raf).

### Benzene metabolite exposures

Hydroquinone (HQ), catechol (CAT) and 1,2,4-benzenetriol (BT) (Sigma-Aldrich, St Louis, MO) stock solutions were prepared in sterile nuclease-free water (ISC BioExpress, Kaysville, UT) and protected from light until use.

### Deletion strain growth curve assays

Yeast strains were pre-grown to mid-log phase, diluted to an optical density at 600 nm (OD_600_) of 0.0165, and dispensed into different wells of a 48-well plate (non-treated polystyrene, Grenier Bio-One, Monroe, NC). Benzene metabolite stock solutions were added to the desired final concentrations with at least two replicates per dose. Plates were incubated in a GENios microplate reader (Tecan, Durham, NC) set to 30°C with intermittent shaking. OD_595_ measurements were taken at 15-minute intervals for a period of 24 hours. Raw absorbance data were averaged for all replicates, background corrected, and plotted as a function of time. The area under the curve (AUC) was calculated with Excel 2008 (Microsoft Corporation, Redmond, WA), as a measure of growth, and expressed as a percentage of the control. AUCs were compared with either one- or two-way ANOVA followed by Dunnett or Bonferroni post-tests, respectively, using GraphPad Prism version 5.01 (GraphPad Software, La Jolla, CA). Data for each strain is derived from three independent biological replicates.

### Overexpression strain growth curve assays

Yeast overexpression strains were pre-grown overnight to stationary phase in SC-ura 2% dextrose, then diluted 1∶100 in SC-ura 2% raffinose and grown overnight again to alleviate glucose repression. Cells were then diluted in YPGal+Raf to induce protein overexpression, and grown for 5 hours to mid-log phase. Cells were subsequently diluted to an optical density at 600 nm (OD_600_) of 0.0165 in YPGal+Raf, and dispensed into different wells of a 48-well plate. Benzene metabolite treatment, plate measurement and data processing were all carried out in the same manner as for the deletion strain growth curve assays.

### Parallel analysis of yeast deletion mutants

Pooled growth of homozygous deletion strains, genomic DNA extraction, barcode amplification, and Affymetrix TAG4 array hybridization were performed as previously described [21]. Raw and processed data files are available at the Gene Expression Omnibus (GEO) database.

### Differential strain sensitivity analysis (DSSA)

Raw TAG4 array data were processed and significant strains identified as previously described [21]. Briefly, a fitness score (ave(log_2_{Y|X = treated})-ave(log_2_{Y|X = control}), the difference in the mean of the log_2_ hybridization signal between treatment and control) was calculated for each deletion strain, to identify those differentially sensitive. This fitness score is an indicator of the effect of the benzene metabolite on the growth of the deletion strain. A negative fitness score indicates the deletion strain is sensitive to benzene metabolite treatment, and that the gene product absent in that strain is likely required for tolerance to that metabolite.

### MIPS functional classification scoring

Data sets from DSSA were verified for enrichment of any particular biological attribute by identifying significantly overrepresented MIPS (Munich Information Center for Protein Sequences) Functional Classification categories [93] by a hypergeometric distribution using the Functional Specification resource, FunSpec (http://funspec.med.utoronto.ca/) [94], with a p-value cutoff of 0.01.

### Pathway enrichment and hierarchical clustering

Raw TAG4 array data were normalized using the Faster Cyclic Loess Method [95] through the fastlo package in R [96], adapted for the TAG4 chip design [97]. Normalization for each benzene metabolite was performed separately and each normalization set also included control chips. For the purpose of normalization, hybridization signals of UP and DOWN barcodes of the same deletion strain were treated as separate measurements and then grouped following normalization.

The normalized data for each benzene metabolite was used in a pathway enrichment method called Structurally Enhanced Pathway Enrichment Analysis (SEPEA), [98]. The biochemical pathways chosen were from the *S. cerevisiae* KEGG pathway database [99]. SEPEA differs from other pathway enrichment methods in that it considers the network structure of the various pathways in the analyses – pathways where perturbed genes (whose deletion strain hybridization signal is altered as a result of metabolite treatment) are a close relative to each other in a graph/network sense are assigned more significance. The gene-wise statistic chosen to be used by SEPEA was the non-parametric Kruskal-Wallis anova statistic [100], which compares medians of four groups of treatments (control, 25% IC_20_, 50% IC_20_ and IC_20_ concentrations) with 12, 3, 3, 3 replicates in each of the groups respectively. Several null hypotheses for SEPEA have been previously described [98]. In this study, SEPEA_NT2 with 10̂5 permutations was chosen for analyses. For each pathway, the null hypothesis tested by SEPEA_NT2 is that the growth phenotype corresponding to deletion strains associated with each of the genes involved in the pathway is independent of the administered dose of the given benzene metabolite. SEPEA_NT2 was coded in Java programming language [101].

Not all genes present in the KEGG database have associated experimental measurements, due to the use of only the non-essential deletion collection of strains in this study. We therefore selected KEGG pathways with TAG4 array data for at least 60% of their genes. This resulted in 43 pathways, whose associated SEPEA p-values were negative log_10_ transformed and hierarchically clustered in Cluster 3.0 [102], using Spearman rank correlation as a measure of distance and average linkage for forming clustering. The resultant clustergram was viewed in TreeView [102].

### Network clustering

The manuscript (Thomas, R. *et al.*) describing the statistical methodology for network clustering is in preparation. The methodology uses an overall system-wide interaction network as a basis for the analyses. In this analysis, the protein interaction network from the STRING database [103] is used. Associated with each interaction in STRING is a score (ranging from 0–1000) for how likely it is to be a true positive. *S. cerevisiae* protein interactions with a score cutoff of 700 (defined as “high-confidence”) were used. Approximately 4000 proteins with 150000 interactions represent this interaction network; these proteins formed the network nodes. The degree of a node is the number of interactions that it is involved in. The node degree distribution followed more or less a power-law distribution. There were a relatively small number of nodes involved in a large number of interactions. Such nodes are referred to as hub nodes.

Significant genes identified in each of the metabolite treatments were determined by fitting gene-specific mixed effects models to the previously described fastlo normalized data. The model takes into account correlation between UP and DOWN barcode measurements of the same deletion strain by accounting for the random chip effect: 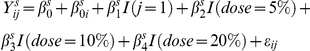 where 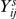 is an expression for a strain *s* on a chip *i*. A subscript *j* indicates a barcode measurement (UP or DOWN). The estimates for this saturated model are:


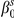
 is the mean of the UP barcode measurements of the control chips


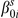
 is the adjustment for a chip (random effect)


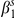
 is the average difference between UP and DOWN barcode measurements (fixed effect)


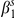
- 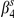 are effects of the doses of the benzene metabolites (fixed effects)

To get to the parameters of interest (dose effects adjusted for the chip and barcode type) we computed a Likelihood Ratio Statistic comparing the full model (above) with the following reduced model: 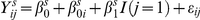



The mixed models were estimated using the nlme package in R [104]. A Holm step-down multiple testing correction (using the multtest package in R [105]) was applied to the p-values that were based on Likelihood Ratio Statistic; the resulting set of genes (deletion strains) was obtained from a 0.05 cut-off.

For the analysis across the metabolites, deletion strains sensitive to at least one of the metabolites were selected. Data for the different generation points (5 g and 15 g) were considered independently.

These metabolite-specific data sets were mapped onto the system-wide interaction network. The goal of the network clustering analysis was to find sub-networks in this system-wide network that are enriched with targets of different combinations of metabolites. The significance of different clusters is based on the overall neighborhood characteristics of the nodes of the interaction network. For example, by random chance it is likely that a randomly picked node is closer to a given hub node than a non-hub node. The analyses were coded in Java programming language [101]. Clusters (sub-networks) were visualized using Cytoscape version 2.8.0 [106]. Enrichment analysis for identification of significantly overrepresented Gene Ontology (GO) Biological Processes [107] within clusters was conducted using the R package topGO [108].

### Fluorescence microscopy

Yap1-GFP and Yap3-GFP were grown to mid-log phase and 300 µl of cells were treated with the IC_20_ dose of HQ, CAT or BT (see Table 1) for 30 minutes. 3 µl of cells were transferred to glass slides following treatment, and microscopy was performed on a Deltavision Spectris DV4 deconvolution microscope (Applied Precision Instruments, Issaquah, WA). Images were not processed.

### Domain prediction, protein identity calculation and sequence alignment

Domain predictions for Pst2p, Ycp4p and Rfs1p were generated using the Ginzu process of the Robetta full-chain protein structure prediction server (http://robetta.bakerlab.org) [109]. Sequence identity between proteins was calculated with BLASTp (http:// blast.ncbi.nlm.nih.gov/Blast.cgi?PAGE = Proteins), using default parameters. Sequences were aligned with ClustalW2 (http://www.ebi.ac.uk/Tools/clustalw2/index.html), using default parameters.

## Supporting Information

Figure S1 **Dose determination of hydroquinone (HQ) for parallel analysis studies.** Growth curve assay for BY4743 wild type treated with increasing concentrations of HQ in YPD media. Measurements of the optical density at 595 nm were taken at 15-minute intervals, with each point in the curve representing the average of three replicate measurements in the microplate. Standard error was omitted from the graph for clarity. Total cell growth in 24 h was determined by calculating the area under the curve (AUC) for each of the growth curves. The selected exposures concentrations were 1,2 and 4 mM HQ.
(PDF)

Figure S2 **Dose determination of catechol (CAT) for parallel analysis studies.** Growth curve assay for BY4743 wild type treated with increasing concentrations of CAT in YPD media. Measurements of the optical density at 595 nm were taken at 15-minute intervals, with each point in the curve representing the average of three replicate measurements in the microplate. Standard error was omitted from the graph for clarity. Total cell growth in 24 h was determined by calculating the area under the curve (AUC) for each of the growth curves. The selected exposures concentrations were 0.55,1.1 and 2.2 mM CAT.
(PDF)

Figure S3 **Dose determination of 1,2,4-benzenetriol (BT) for parallel analysis studies.** Growth curve assay for BY4743 wild type treated with increasing concentrations of BT in YPD media. Measurements of the optical density at 595 nm were taken at 15-minute intervals, with each point in the curve representing the average of three replicate measurements in the microplate. Standard error was omitted from the graph for clarity. Total cell growth in 24 h was determined by calculating the area under the curve (AUC) for each of the growth curves. The selected exposures concentrations were 87.5,175 and 350 µM BT.
(PDF)

Figure S4 **Growth curve analysis of glutathione pathway mutants.** The AUC was calculated for each strain after 24 h of exposure to the indicated doses of HQ, CAT and BT. The bars represent mean AUC as a percentage of the untreated for each strain with standard error of three replicates. Sensitivity was determined by comparison to the wild type strain (gray bars = wild type; white bars = indicated deletion strain). There is no significant requirement for the glutaredoxin pathway for tolerance of any of the metabolites tested at the IC_20_ dose, although *glr1*Δ is moderately sensitive to HQ, and *gsh1*Δ shows some sensitivity to both CAT and BT.
(PDF)

Figure S5 **Growth curve analysis of iron homeostasis mutants.** The AUC was calculated for each strain after 24 h of exposure to the indicated doses of HQ, CAT and BT. The bars represent mean AUC as a percentage of the untreated for each strain with standard error of three replicates. Sensitivity was determined by comparison to the wild type strain (gray bars = wild type; white bars = indicated deletion strain). Both *aft1*Δ and *cup5*Δ are extremely sensitive to HQ, CAT and BT. The negative AUC values for *cup5*Δ treated with BT are due to the slow growth (and so low AUC values) of *cup5*Δ combined with the absorbance at 595 nm of a BT-derived compound. It was not possible to assess *aft1*Δ when treated with BT, due to insufficient growth of an untreated control to use for normalization. However, no growth of *aft1*Δ was ever seen following BT treatment at any dose.
(PDF)

Figure S6 **Growth curve analysis of** ***rfs1***Δ **and** ***lot6***Δ**.** The AUC was calculated for each strain after 24 h of exposure to the indicated doses of HQ, CAT and BT. The bars represent mean AUC as a percentage of the untreated for each strain with standard error of three replicates. Sensitivity was determined by comparison to the wild type strain (gray bars = wild type; white bars = indicated deletion strain). Deletion of *RFS1* has no effect on HQ, CAT or BT tolerance. The sensitivity of *lot6*Δ to CAT suggests that Lot6p may reduce the quinone form of CAT (1,2-BT) *in vivo*.
(PDF)

Table S1 **Complete list of yeast genes (n = 478) identified by DSSA after treatment with hydroquinone (HQ), ranked by the number of hits in 6 treatments.** Yeast pools were exposed to 3 different concentrations of hydroquinone for two generation-points, for a total of 6 treatments. The yeast ORFs/genes correspond to deletion strains that exhibited a significant change in growth in at least one treatment with hydroquinone (q<0.05). Numeric values are fitness scores (log_2_ ratios) calculated only for significant genes in each individual treatment. Empty cells indicate that the gene was not significant in that particular treatment.
(DOC)

Table S2 **Complete list of yeast genes (n = 528) identified by DSSA after treatment with catechol (CAT), ranked by the number of hits in 6 treatments.** Yeast pools were exposed to 3 different concentrations of catechol for two generation-points, for a total of 6 treatments. The yeast ORFs/genes correspond to deletion strains that exhibited a significant change in growth in at least one treatment with catechol (q<0.05). Numeric values are fitness scores (log_2_ ratios) calculated only for significant genes in each individual treatment. Empty cells indicate that the gene was not significant in that particular treatment.
(DOC)

Table S3 **Complete list of yeast genes (n = 604) identified by DSSA after treatment with 1,2,4-benzenetriol (BT), ranked by the number of hits in 6 treatments.** Yeast pools were exposed to 3 different concentrations of 1,2,4-benzenetriol for two generation-points, for a total of 6 treatments. The yeast ORFs/genes correspond to deletion strains that exhibited a significant change in growth in at least one treatment with 1,2,4-benzenetriol (q<0.05). Numeric values are fitness scores (log_2_ ratios) calculated only for significant genes in each individual treatment. Empty cells indicate that the gene was not significant in that particular treatment.
(DOC)

Table S4 **Complete results of KEGG pathway enrichment analysis.** This study provided data for ∼900 of the ∼2000 genes represented in the 99 annotated KEGG pathways for *S. cerevisiae*. Highlighted in yellow are the 43 KEGG pathways for which at least 60% of genes annotated to that pathway are present in the PDA screen data - these pathways are clustered in Figure 3A.
(XLS)

Table S5 **Complete results of network clustering analysis at 5 generations.** 19 sub-networks (clusters) were identified at 5 g. The four most significantly enriched GO Biological Processes are shown for each network.
(XLS)

Table S6 **Complete results of network clustering analysis at 15 generations.** 41 sub-networks (clusters) were identified at 15 g. The four most significantly enriched GO Biological Processes are shown for each network.
(XLS)
