# Supplementary material for: Genome-Wide Functional Profiling Reveals Genes Required for Tolerance to Benzene Metabolites in Yeast
Source: PLoS One. 2011 Aug 30;6(8):e24205. doi: 10.1371/journal.pone.0024205 (PMC3166172; doi:10.1371/journal.pone.0024205)
Supplement: Figure S2 — Dose determination of catechol (CAT) for parallel analysis studies. Growth curve assay for BY4743 wild type treated with increasing concentrations of CAT in YPD media. Measurements of the optical density at 595 nm were taken at 15-minute intervals, with each point in the curve representing the average of three replicate measurements in the microplate. Standard error was omitted from the graph for clarity. Total cell growth in 24 h was determined by calculating the area under the curve (AUC) for each of the growth curves. The selected exposures concentrations were 0.55,1.1 and 2.2 mM CAT. (PDF) [file pone.0024205.s002.pdf]

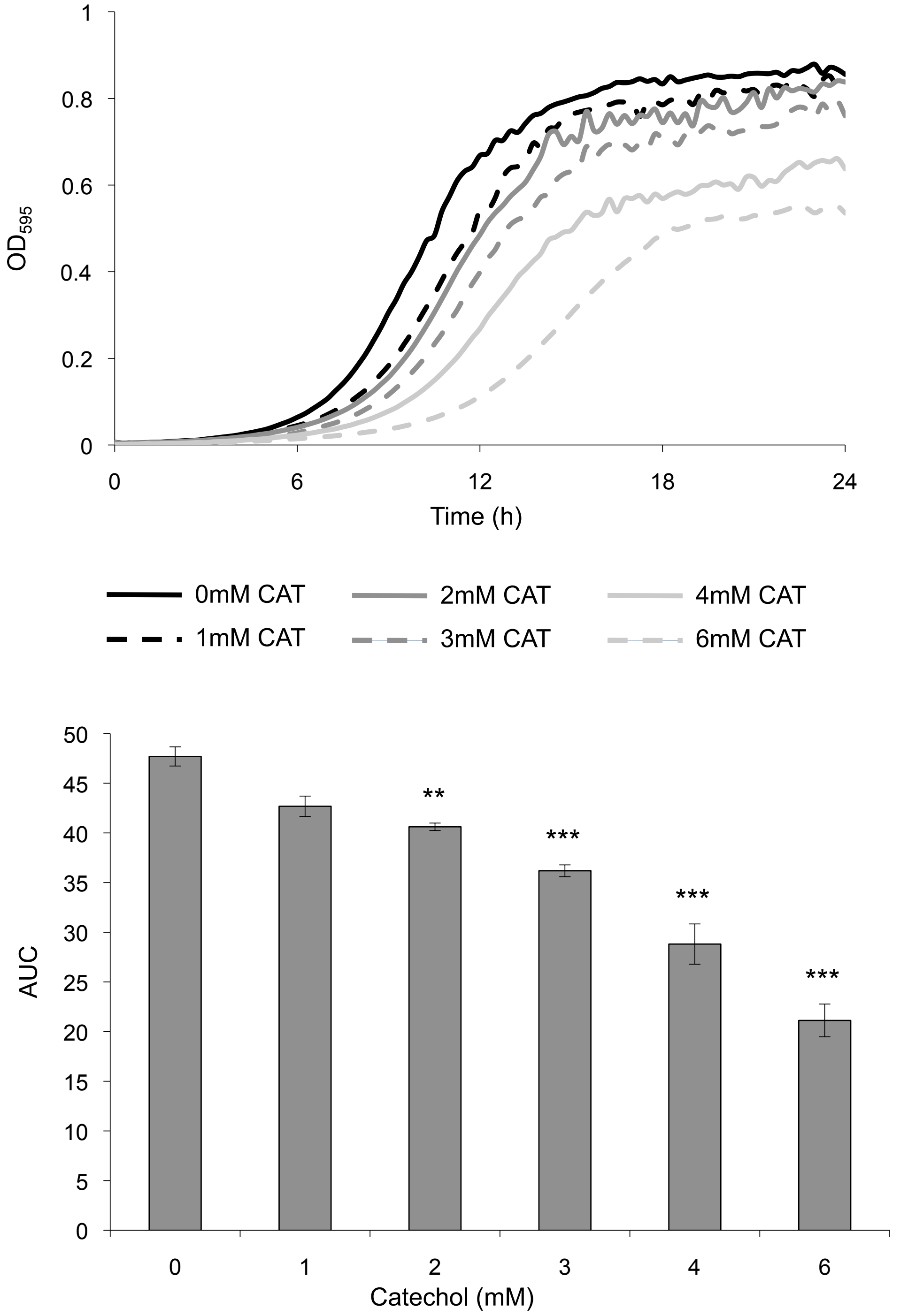

*** p < 0.001 ; ** 0.001 < p < 0.01

Figure S2. Dose determination of catechol (CAT) for parallel analysis studies. Growth curve assay for BY4743 wild type treated with increasing concentrations of CAT in YPD media. Measurements of the optical density at 595nm were taken at 15-minute intervals, with each point in the curve representing the average of three replicate measurements in the microplate. Standard error was omitted from the graph for clarity. Total cell growth in 24h was determined by calculating the area under the curve (AUC) for each of the growth curves. The selected exposures concentrations were 0.55,1.1 and 2.2mM CAT.
