# Supplementary material for: Genome-Wide Functional Profiling Reveals Genes Required for Tolerance to Benzene Metabolites in Yeast
Source: PLoS One. 2011 Aug 30;6(8):e24205. doi: 10.1371/journal.pone.0024205 (PMC3166172; doi:10.1371/journal.pone.0024205)
Supplement: Figure S4 — Growth curve analysis of glutathione pathway mutants. The AUC was calculated for each strain after 24 h of exposure to the indicated doses of HQ, CAT and BT. The bars represent mean AUC as a percentage of the untreated for each strain with standard error of three replicates. Sensitivity was determined by comparison to the wild type strain (gray bars = wild type; white bars = indicated deletion strain). There is no significant requirement for the glutaredoxin pathway for tolerance of any of the metabolites tested at the IC20 dose, although glr1Δ is moderately sensitive to HQ, and gsh1Δ shows some sensitivity to both CAT and BT. (PDF) [file pone.0024205.s004.pdf]

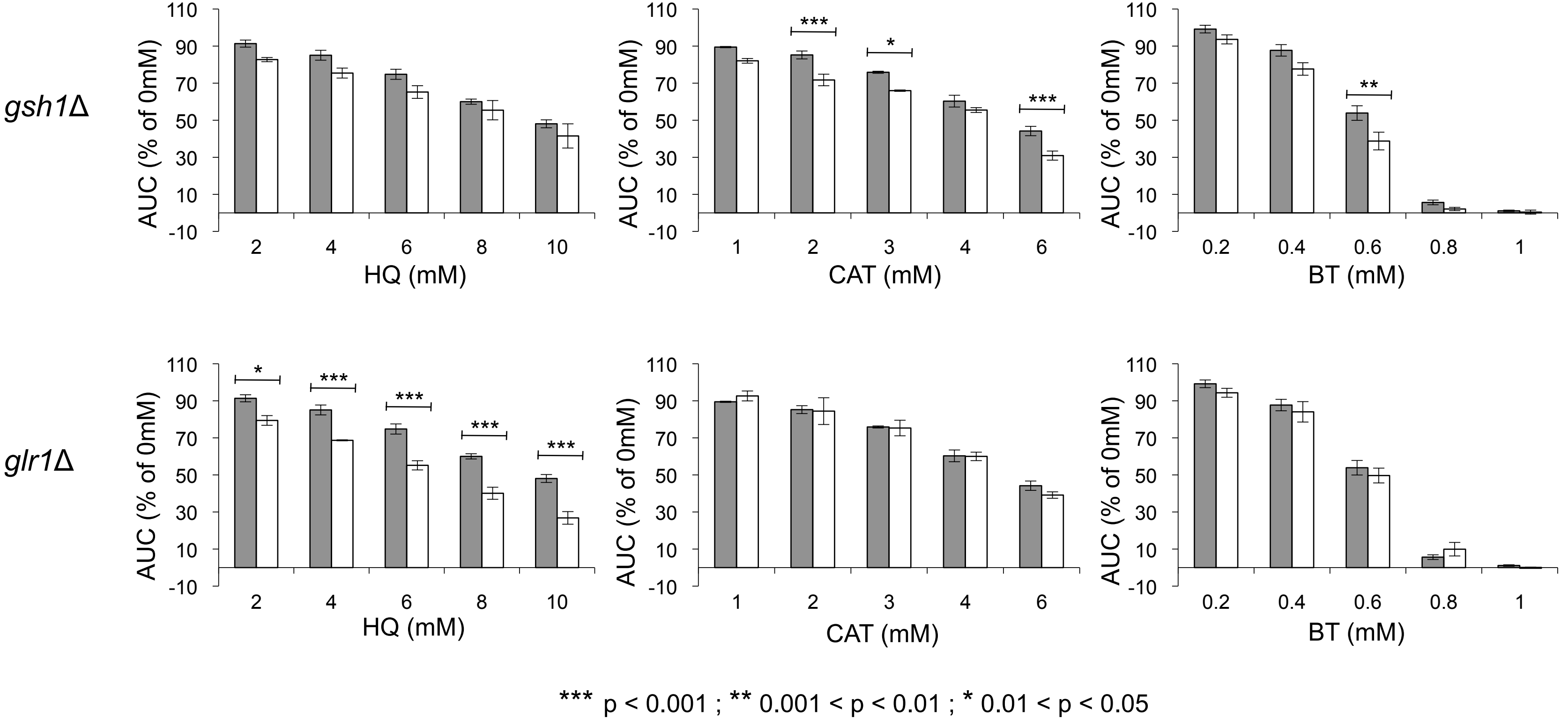

Figure S4. Growth curve analysis of glutathione pathway mutants. The AUC was calculated for each strain after 24h of exposure to the indicated doses of HQ, CAT and BT. The bars represent mean AUC as a percentage of the untreated for each strain with standard error of three replicates. Sensitivity was determined by comparison to the wild type strain (gray bars = wild type; white bars = indicated deletion strain). There is no significant requirement for the glutaredoxin pathway for tolerance of any of the metabolites tested at the IC_{20} dose, although *glr1*Δ is moderately sensitive to HQ, and *gsh1*Δ shows some sensitivity to both CAT and BT.
