# Supplementary material for: Genome-Wide Functional Profiling Reveals Genes Required for Tolerance to Benzene Metabolites in Yeast
Source: PLoS One. 2011 Aug 30;6(8):e24205. doi: 10.1371/journal.pone.0024205 (PMC3166172; doi:10.1371/journal.pone.0024205)
Supplement: Figure S5 — Growth curve analysis of iron homeostasis mutants. The AUC was calculated for each strain after 24 h of exposure to the indicated doses of HQ, CAT and BT. The bars represent mean AUC as a percentage of the untreated for each strain with standard error of three replicates. Sensitivity was determined by comparison to the wild type strain (gray bars = wild type; white bars = indicated deletion strain). Both aft1Δ and cup5Δ are extremely sensitive to HQ, CAT and BT. The negative AUC values for cup5Δ treated with BT are due to the slow growth (and so low AUC values) of cup5Δ combined with the absorbance at 595 nm of a BT-derived compound. It was not possible to assess aft1Δ when treated with BT, due to insufficient growth of an untreated control to use for normalization. However, no growth of aft1Δ was ever seen following BT treatment at any dose. (PDF) [file pone.0024205.s005.pdf]

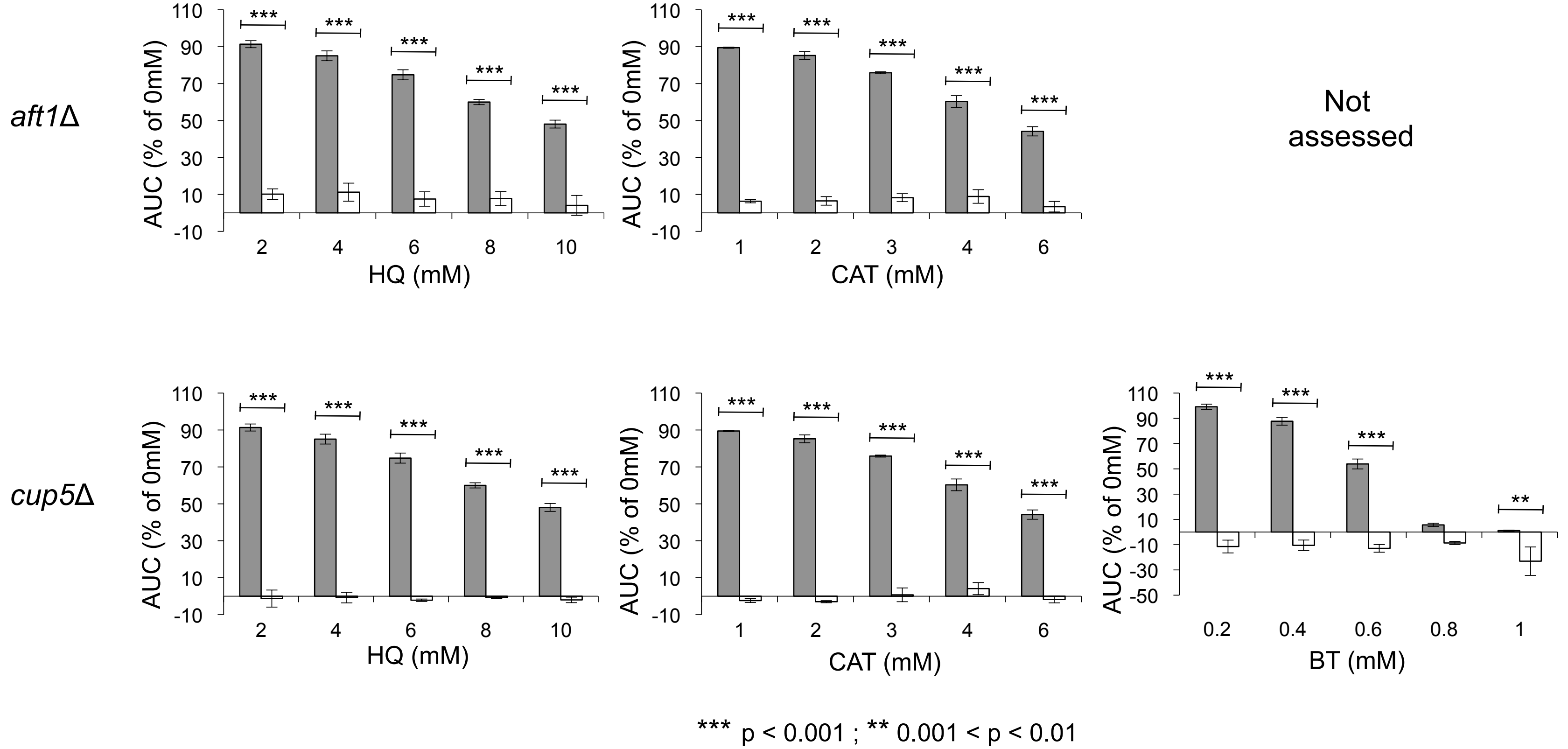

Figure S5. Growth curve analysis of iron homeostasis mutants. The AUC was calculated for each strain after 24h of exposure to the indicated doses of HQ, CAT and BT. The bars represent mean AUC as a percentage of the untreated for each strain with standard error of three replicates. Sensitivity was determined by comparison to the wild type strain (gray bars = wild type; white bars = indicated deletion strain). Both *aft1*Δ and *cup5*Δ are extremely sensitive to HQ, CAT and BT. The negative AUC values for *cup5*Δ treated with BT are due to the slow growth (and so low AUC values) of *cup5*Δ combined with the absorbance at 595nm of a BT-derived compound. It was not possible to assess *aft1*Δ when treated with BT, due to insufficient growth of an untreated control to use for normalization. However, no growth of *aft1*Δ was ever seen following BT treatment at any dose.
