# Supplementary material for: Genome-Wide Functional Profiling Reveals Genes Required for Tolerance to Benzene Metabolites in Yeast
Source: PLoS One. 2011 Aug 30;6(8):e24205. doi: 10.1371/journal.pone.0024205 (PMC3166172; doi:10.1371/journal.pone.0024205)
Supplement: Figure S6 — Growth curve analysis of rfs1Δ and lot6Δ. The AUC was calculated for each strain after 24 h of exposure to the indicated doses of HQ, CAT and BT. The bars represent mean AUC as a percentage of the untreated for each strain with standard error of three replicates. Sensitivity was determined by comparison to the wild type strain (gray bars = wild type; white bars = indicated deletion strain). Deletion of RFS1 has no effect on HQ, CAT or BT tolerance. The sensitivity of lot6Δ to CAT suggests that Lot6p may reduce the quinone form of CAT (1,2-BT) in vivo. (PDF) [file pone.0024205.s006.pdf]

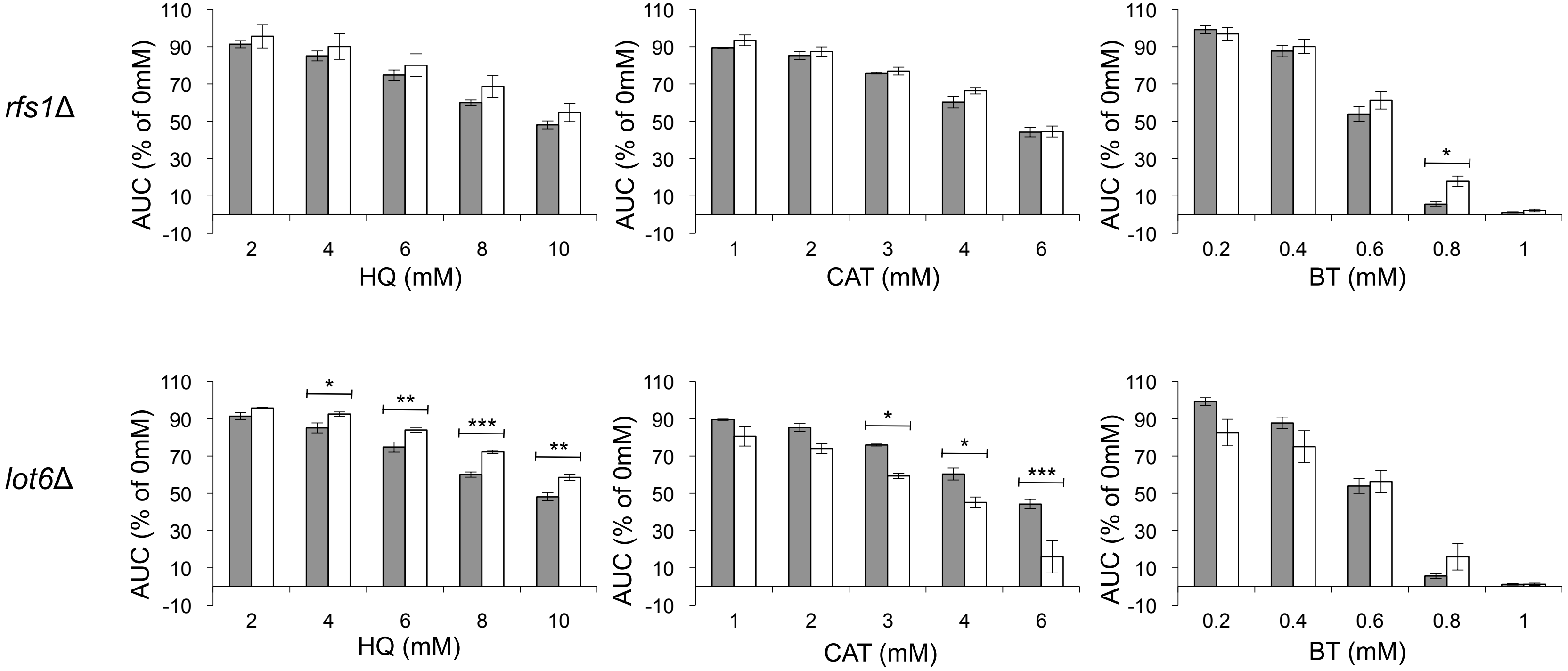

*** $p < 0.001$; ** $0.001 < p < 0.01$; * $0.01 < p < 0.05$

Figure S6. Growth curve analysis of *rfs1*Δ and *lot6*Δ. The AUC was calculated for each strain after 24h of exposure to the indicated doses of HQ, CAT and BT. The bars represent mean AUC as a percentage of the untreated for each strain with standard error of three replicates. Sensitivity was determined by comparison to the wild type strain (gray bars = wild type; white bars = indicated deletion strain). Deletion of *RFS1* has no effect on HQ, CAT or BT tolerance. The sensitivity of *lot6*Δ to CAT suggests that Lot6p may reduce the quinone form of CAT (1,2-BT) *in vivo*.
