# Supplementary material for: Genome-Wide Functional Profiling Reveals Genes Required for Tolerance to Benzene Metabolites in Yeast
Source: PLoS One. 2011 Aug 30;6(8):e24205. doi: 10.1371/journal.pone.0024205 (PMC3166172; doi:10.1371/journal.pone.0024205)
Supplement: Table S1 — Complete list of yeast genes (n = 478) identified by DSSA after treatment with hydroquinone (HQ), ranked by the number of hits in 6 treatments. Yeast pools were exposed to 3 different concentrations of hydroquinone for two generation-points, for a total of 6 treatments. The yeast ORFs/genes correspond to deletion strains that exhibited a significant change in growth in at least one treatment with hydroquinone (q<0.05). Numeric values are fitness scores (log2 ratios) calculated only for significant genes in each individual treatment. Empty cells indicate that the gene was not significant in that particular treatment. (DOC) [file pone.0024205.s007.doc]

**Table S1. Complete list of yeast genes (n = 478) identified by DSSA after treatment with hydroquinone (HQ), ranked by the number of hits in 6 treatments.** Yeast pools were exposed to 3 different concentrations of hydroquinone for two generation-points, for a total of 6 treatments. The yeast ORFs / genes correspond to deletion strains that exhibited a significant change in growth in at least one treatment with hydroquinone (q < 0.05). Numeric values are fitness scores (log_2_ ratios) calculated only for significant genes in each individual treatment. Empty cells indicate that the gene was not significant in that particular treatment.

|  |  | 5 generations | | | 15 generations | | |  |
| --- | --- | --- | --- | --- | --- | --- | --- | --- |
| ORF | Gene | 25% IC_20_ | 50% IC_20_ | IC_20_ | 25% IC_20_ | 50% IC_20_ | IC_20_ | # of hits |
|  |  | 1mM | 2mM | 4mM | 1mM | 2mM | 4mM |  |
| *YOL081W* | *IRA2* | -2.5 | -3.3 | -4.1 | -4 | -6.2 | -2.6 | 6 |
| *YOL085C* |  | -1.95 | -2.1 | -3 | -4.35 | -4.85 | -3.3 | 6 |
| *YOR314W* |  | 2.3 | 2.5 | 2.4 | 2.7 | 2.4 | 2.5 | 6 |
| *YDR458C* | *HEH2* | 2.65 | 2.95 | 2.85 | 3.9 | 2.9 | 3 | 6 |
| *YPL157W* | *TGS1* | 3.3 | 2.65 | 2.85 | 3.7 | 3.2 | 4.1 | 6 |
| *YDL223C* | *HBT1* | 2.9 | 2.9 | 2.2 | 2.6 | 2.8 | 2.6 | 6 |
| *YHL009C* | *YAP3* | -2.3 | -2.3 | -2.9 | -4.15 | -3.6 |  | 5 |
| *YJL121C* | *RPE1* | -2.2 | -2.4 | -2.85 | -3.3 | -3 |  | 5 |
| *YJR088C* |  | 2.6 | 2.8 | 2.9 | 3.7 | 3.3 |  | 5 |
| *YDR032C* | *PST2* |  | -2.3 | -3 | -2.3 | -3.4 | -3.2 | 5 |
| *YOL013C* | *HRD1* |  | -1.5 | -2.1 | -4.2 | -3.8 | -3.1 | 5 |
| *YOL079W* |  |  | -2.4 | -2.45 | -3.55 | -2.95 | -3.35 | 5 |
| *YOL025W* | *LAG2* |  | -1.7 | -1.9 | -3.5 | -3.3 | -2.55 | 5 |
| *YNL140C* |  | 1.8 |  | 2 | 4.2 | 4.4 | 5 | 5 |
| *YDR153C* | *ENT5* | 2.5 | 2.3 | 2.8 | 2.9 | 2.9 |  | 5 |
| *YPL178W* | *CBC2* | 2.6 |  | 2.9 | 3 | 3 | 3.3 | 5 |
| *YOR133W* | *EFT1* |  | 2.8 | 3.2 | 3.5 | 3 | 3.4 | 5 |
| *YCR073W-A* | *SOL2* | 2.5 | 2.9 | 3.4 |  |  | 3.4 | 4 |
| *YJR044C* | *VPS55* | 2.6 | 2.5 | 2.9 |  | 2.5 |  | 4 |
| *YBR213W* | *MET8* | 2.6 | 2.4 | 2.3 | 3 |  |  | 4 |
| *YGL226C-A* | *OST5* | 3.1 | 2.6 | 3 | 2.6 |  |  | 4 |
| *YJL120W* |  |  | -1.9 | -2.25 | -3.9 | -3.8 |  | 4 |
| *YGR070W* | *ROM1* |  | 1.9 | 2.1 | 2.6 | 2.7 |  | 4 |
| *YPL035C* |  | 2.6 | 3 | 3.1 | 3.1 |  |  | 4 |
| *YIR028W* | *DAL4* | 4.6 | 4.3 | 3.8 | 2.7 |  |  | 4 |
| *YBR170C* | *NPL4* |  | 2.7 | 2.5 | 3.5 |  | 3.5 | 4 |
| *YDL230W* | *PTP1* |  |  | 1.2 | 3.1 | 3.1 | 3.3 | 4 |
| *YMR184W* | *ADD37* |  |  | 1.9 | 2.4 | 2.1 | 2.4 | 4 |
| *YFR018C* |  |  |  | 2.1 | 3 | 3 | 3 | 4 |
| *YMR289W* | *ABZ2* |  |  | 2.1 | 3.9 | 3.4 | 3.3 | 4 |
| *YKR078W* |  |  |  | 2.3 | 2.7 | 2.4 | 2.7 | 4 |
| *YGR130C* |  | -2.1 | -2.2 | -2.4 |  |  |  | 3 |
| *YLR077W* | *FMP25* | -1.6 | -1.5 | -1.1 |  |  |  | 3 |
| *YKL147C* |  | -1.6 | -1.4 | -1.3 |  |  |  | 3 |
| *YCR068W* | *ATG15* | -1.6 | -1.2 | -1.6 |  |  |  | 3 |
| *YMR237W* | *BCH1* | -1.5 | -1.3 | -1.4 |  |  |  | 3 |
| *YOR084W* |  | -1.3 | -1.4 | -1.3 |  |  |  | 3 |
| *YBR298C* | *MAL31* | 1.8 | 2.1 | 2.1 |  |  |  | 3 |
| *YLR169W* |  | 1.9 | 1.7 | 1.8 |  |  |  | 3 |
| *YDR175C* | *RSM24* | 2 | 2.1 | 1.9 |  |  |  | 3 |
| *YOR019W* |  | 2 | 2.1 | 2.3 |  |  |  | 3 |
| *YGR041W* | *BUD9* | 2.2 | 2.5 | 2.5 |  |  |  | 3 |
| *YOR352W* |  | 2.3 | 2.6 | 2.5 |  |  |  | 3 |
| *YDR105C* | *TMS1* | 2.5 | 2.8 | 3.1 |  |  |  | 3 |
| *YCR006C* |  | 2.5 | 2.7 | 2.9 |  |  |  | 3 |
| *YGR282C* | *BGL2* | 2.6 | 2.6 | 2.5 |  |  |  | 3 |
| *YDL216C* | *RRI1* | 2.7 | 2.5 | 2.4 |  |  |  | 3 |
| *YGL138C* |  | 2.9 | 3.1 | 3.2 |  |  |  | 3 |
| *YER002W* | *NOP16* | 2.9 | 3.1 | 2.6 |  |  |  | 3 |
| *YDR179C* | *CSN9* | 2.9 | 3.1 |  | -2 |  |  | 3 |
| *YKL166C* | *TPK3* | 3.1 | 2.9 | 2.8 |  |  |  | 3 |
| *YER169W* | *RPH1* | 3.2 | 3.1 | 3.6 |  |  |  | 3 |
| *YBR187W* | *GDT1* | 3.4 | 3.6 | 3.7 |  |  |  | 3 |
| *YCR004C* | *YCP4* |  | -1.5 | -1.5 |  |  | -3.6 | 3 |
| *YOR325W* |  |  | -1.3 | -1.6 |  |  | -2.2 | 3 |
| *YIL036W* | *CST6* |  | 1.7 |  | 3.3 | 3.3 |  | 3 |
| *YDR163W* | *CWC15* |  | 2.2 | 2.5 |  |  | 2.7 | 3 |
| *YDL020C* | *RPN4* |  |  | -1.6 | -3.8 | -3.4 |  | 3 |
| *YNL148C* | *ALF1* | -1.9 |  | -2.5 | -3.9 |  |  | 3 |
| *YGR139W* |  | -1.7 | -1.5 | -1.5 |  |  |  | 3 |
| *YGL004C* | *RPN14* | 1.8 | 1.8 | 1.8 |  |  |  | 3 |
| *YOR364W* |  | 2 | 2.2 | 2.2 |  |  |  | 3 |
| *YJR010C-A* | *SPC1* | 2.5 | 2.1 | 2.5 |  |  |  | 3 |
| *YKL221W* | *MCH2* | 2.9 | 2.5 | 2.6 |  |  |  | 3 |
| *YEL012W* | *UBC8* | 3 | 3.2 | 3.5 |  |  |  | 3 |
| *YPL274W* | *SAM3* | 3.4 | 3.1 | 2.9 |  |  |  | 3 |
| *YMR272C* | *SCS7* | 3.7 | 3.1 |  | 1.7 |  |  | 3 |
| *YML070W* | *DAK1* | 4 | 4.1 | 3.8 |  |  |  | 3 |
| *YMR238W* | *DFG5* |  | 1.7 | 1.6 | 2.4 |  |  | 3 |
| *YMR244C-A* |  |  | 2.4 | 2.2 |  |  | 2.2 | 3 |
| *YHR030C* | *SLT2* |  |  | -1.6 | -2.7 |  | -3.3 | 3 |
| *YKL040C* | *NFU1* |  |  | -1.4 |  | -1.9 | -2.1 | 3 |
| *YMR063W* | *RIM9* |  |  | 1.5 |  | 2.6 | 3.1 | 3 |
| *YGR035C* |  |  |  | 1.8 |  | 2.2 | 2.3 | 3 |
| *YLR335W* | *NUP2* |  |  | 1.8 | 2.8 |  | 2.2 | 3 |
| *YKR035C* | *OPI8* |  |  | 2.9 | 2.4 |  | 2.8 | 3 |
| *YIL162W* | *SUC2* |  |  |  | -3.8 | -4.4 | -4.1 | 3 |
| *YMR022W* | *QRI8* |  |  |  | -2.4 | -2.4 | -2.2 | 3 |
| *YBR035C* | *PDX3* |  |  |  | -2.4 | -2.4 | -2.3 | 3 |
| *YPR074C* | *TKL1* |  |  |  | -2.3 | -3.1 | -3.8 | 3 |
| *YPR200C* | *ARR2* |  |  |  | 1.9 | 1.8 | 2 | 3 |
| *YBR273C* | *UBX7* |  |  |  | 2 | 2.3 | 2.1 | 3 |
| *YBR225W* |  |  |  |  | 2.3 | 2.2 | 2.5 | 3 |
| *YPL052W* | *OAZ1* |  |  |  | 2.3 | 2.3 | 2.3 | 3 |
| *YOL101C* | *IZH4* |  |  |  | 2.3 | 2.5 | 2.8 | 3 |
| *YHL037C* |  |  |  |  | 2.4 | 2.5 | 2.7 | 3 |
| *YJL217W* |  |  |  |  | 2.4 | 2.6 | 2.7 | 3 |
| *YER080W* | *FMP29* |  |  |  | 2.5 | 2.2 | 2.3 | 3 |
| *YDR314C* | *RAD34* |  |  |  | 2.3 | 3.1 | 2.5 | 3 |
| *YBR184W* |  |  |  |  | 2.5 | 2.3 | 2.5 | 3 |
| *YNR019W* | *ARE2* |  |  |  | 2.5 | 2.9 | 2.7 | 3 |
| *YNR024W* |  |  |  |  | 2.6 | 2.5 | 2 | 3 |
| *YKR096W* |  |  |  |  | 2.6 | 2.9 | 3.3 | 3 |
| *YDR447C* | *RPS17B* |  |  |  | 2.7 | 2.2 | 2.2 | 3 |
| *YDR354W* | *TRP4* |  |  |  | 2.7 | 2.6 | 3 | 3 |
| *YJL218W* |  |  |  |  | 2.7 | 2.4 | 2.6 | 3 |
| *YGL232W* | *TAN1* |  |  |  | 2.7 | 2.7 | 2.5 | 3 |
| *YJR110W* | *YMR1* |  |  |  | 2.8 | 2.5 | 2.3 | 3 |
| *YJL108C* | *PRM10* |  |  |  | 2.8 | 3.2 | 3.2 | 3 |
| *YJL166W* | *QCR8* |  |  |  | 2.9 | 3.1 | 3.6 | 3 |
| *YJR080C* | *FMP26* |  |  |  | 3 | 3.7 | 3 | 3 |
| *YFL055W* | *AGP3* |  |  |  | 3 | 3.4 | 3.8 | 3 |
| *YFL020C* | *PAU5* |  |  |  | 3 | 3.3 | 2.8 | 3 |
| *YIL032C* |  |  |  |  | 3 | 2.5 | 2.4 | 3 |
| *YER087C-A* |  |  |  |  | 3.1 | 2.7 | 2.9 | 3 |
| *YFL011W* | *HXT10* |  |  |  | 3.2 | 3.2 | 2.9 | 3 |
| *YMR291W* |  |  |  |  | 3.2 | 3.2 | 3.6 | 3 |
| *YLR042C* |  |  |  |  | 3.3 | 3.1 | 3.2 | 3 |
| *YMR042W* | *ARG80* |  |  |  | 3.5 | 3.3 | 3.4 | 3 |
| *YPR128C* | *ANT1* |  |  |  | 3.6 | 3.8 | 3.3 | 3 |
| *YDR312W* | *SSF2* |  |  |  | 3.9 | 4 | 3.4 | 3 |
| *YPR011C* |  |  |  |  | 4.3 | 4.9 | 5.1 | 3 |
| *YFL019C* |  |  |  |  | 4.4 | 4.6 | 5 | 3 |
| *YFL032W* |  |  |  |  | 4.4 | 4 | 4.6 | 3 |
| *YBR113W* |  |  |  |  | 4.4 | 3.6 | 4.5 | 3 |
| *YDL225W* | *SHS1* |  |  |  | -1.8 | -3.2 | -2.9 | 3 |
| *YDR112W* | *IRC2* |  |  |  | -4.1 | -4.4 | -4.3 | 3 |
| *YEL056W* | *HAT2* |  |  |  | -3.6 | -3.7 | -3.8 | 3 |
| *YDR457W* | *TOM1* |  |  |  | -3.1 | -3.2 | -2.2 | 3 |
| *YPL138C* | *SPP1* |  |  |  | -2.8 | -3.3 | -3.2 | 3 |
| *YDR400W* | *URH1* |  |  |  | 2.3 | 2 | 2.2 | 3 |
| *YFL041W* | *FET5* |  |  |  | 2.3 | 2.4 | 2.3 | 3 |
| *YMR299C* | *DYN3* |  |  |  | 2.3 | 2.3 | 1.9 | 3 |
| *YBR149W* | *ARA1* |  |  |  | 2.6 | 2.6 | 2.6 | 3 |
| *YKL096W-A* | *CWP2* |  |  |  | 2.7 | 2.5 | 2.7 | 3 |
| *YMR204C* | *INP1* |  |  |  | 2.8 | 2.7 | 2.7 | 3 |
| *YLR203C* | *MSS51* |  |  |  | 3.3 | 2.6 | 2.8 | 3 |
| *YML119W* |  |  |  |  | 3.3 | 3.1 | 3 | 3 |
| *YDR336W* |  |  |  |  | 3.3 | 3.2 | 3.1 | 3 |
| *YMR114C* |  |  |  |  | 3.5 | 3.4 | 3.7 | 3 |
| *YNL047C* | *SLM2* |  |  |  | 4 | 4 | 4.3 | 3 |
| *YEL028W* |  |  |  |  | 4.1 | 4.3 | 4.1 | 3 |
| *YOL118C* |  |  |  |  | 4.1 | 3.9 | 3.3 | 3 |
| *YJL046W* |  |  |  |  | 4.2 | 4.4 | 3.7 | 3 |
| *YJR079W* |  |  |  |  | 4.3 | 4.4 | 3.9 | 3 |
| *YOR092W* | *ECM3* |  |  |  | 4.4 | 4.6 | 4.1 | 3 |
| *YPR109W* |  |  |  |  | 4.4 | 4.4 | 5.7 | 3 |
| *YMR105C* | *PGM2* |  |  |  | 4.6 | 4.7 | 5.1 | 3 |
| *YDL010W* |  |  |  |  | 4.7 | 4.2 | 4 | 3 |
| *YBR138C* |  |  |  |  | 4.8 | 3.9 | 4.9 | 3 |
| *YKL174C* | *TPO5* |  |  |  | 5.1 | 4.7 | 4.3 | 3 |
| *YDR291W* | *HRQ1* |  |  |  | 5.1 | 4.8 | 4.9 | 3 |
| *YML009C* | *MRPL39* |  |  |  | 5.3 | 5.1 | 4.9 | 3 |
| *YBL065W* |  |  |  |  | 5.8 | 5.4 | 5.7 | 3 |
| *YER019W* | *ISC1* | -2.5 |  | -2.1 |  |  |  | 2 |
| *YKR012C* |  | -1.4 |  | -1 |  |  |  | 2 |
| *YDL211C* |  | -1.3 |  | -1.1 |  |  |  | 2 |
| *YMR256C* | *COX7* | 1.9 | 2.3 |  |  |  |  | 2 |
| *YDR465C* | *RMT2* | 2 |  | 2.1 |  |  |  | 2 |
| *YBR299W* | *MAL32* | 2.3 |  | 2.5 |  |  |  | 2 |
| *YMR304W* | *UBP15* | 2.3 | 2.2 |  |  |  |  | 2 |
| *YBR068C* | *BAP2* |  | -3.2 | -2.8 |  |  |  | 2 |
| *YML008C* | *ERG6* |  | -2.9 | -2 |  |  |  | 2 |
| *YIR023W* | *DAL81* |  | -2.45 | -2.65 |  |  |  | 2 |
| *YHR151C* |  |  | -2.5 | -1.8 |  |  |  | 2 |
| *YKR046C* | *PET10* |  | -1.4 | -1.3 |  |  |  | 2 |
| *YMR283C* | *RIT1* |  | -1.1 | -1.1 |  |  |  | 2 |
| *YGL175C* | *SAE2* |  | 1.6 | 1.5 |  |  |  | 2 |
| *YDR441C* | *APT2* |  | 1.8 | 1.9 |  |  |  | 2 |
| *YOR292C* |  |  | 2 | 2.1 |  |  |  | 2 |
| *YJL131C* |  |  | 2.1 | 2.5 |  |  |  | 2 |
| *YDR025W* | *RPS11A* |  | 2.1 | 2.4 |  |  |  | 2 |
| *YER066C-A* |  |  | 2.1 | 2.5 |  |  |  | 2 |
| *YGL041C* |  |  | 2.2 | 2.2 |  |  |  | 2 |
| *YGL240W* | *DOC1* |  | 2.3 | 2.25 |  |  |  | 2 |
| *YDR283C* | *GCN2* |  | 2.4 | 2.5 |  |  |  | 2 |
| *YIL139C* | *REV7* |  | 2.5 | 2 |  |  |  | 2 |
| *YIL153W* | *RRD1* |  | 2.6 | 2.7 |  |  |  | 2 |
| *YLL043W* | *FPS1* |  | 2.7 | 2.4 |  |  |  | 2 |
| *YLR450W* | *HMG2* |  | 3.1 | 3.1 |  |  |  | 2 |
| *YLR192C* | *HCR1* |  | -2.4 | -2.3 |  |  |  | 2 |
| *YDR132C* |  |  |  | -1.8 | -2.4 |  |  | 2 |
| *YHR013C* | *ARD1* |  | -1.9 | -2.35 |  |  |  | 2 |
| *YNL099C* | *OCA1* |  | -2 | -1.6 |  |  |  | 2 |
| *YOR061W* | *CKA2* |  |  | 1 |  |  | 1.5 | 2 |
| *YJR053W* | *BFA1* |  |  | 1 |  |  | 2 | 2 |
| *YOR366W* |  |  |  | 1.5 | 2.3 |  |  | 2 |
| *YHR014W* | *SPO13* |  |  | 1.5 | 1.7 |  |  | 2 |
| *YDL202W* | *MRPL11* |  |  | 1.5 |  |  | -2.5 | 2 |
| *YGL229C* | *SAP4* |  |  | 2.1 |  | 2.3 |  | 2 |
| *YGL125W* | *MET13* |  |  | 2.3 |  |  | 2.5 | 2 |
| *YOR199W* |  |  |  | 2.4 |  |  | 3 | 2 |
| *YMR280C* | *CAT8* | 2.1 |  | 1.9 |  |  |  | 2 |
| *YGL118C* |  | 2.5 |  | 2.7 |  |  |  | 2 |
| *YKL066W* |  | 3 | 3.5 |  |  |  |  | 2 |
| *YNL305C* |  | 3.7 |  |  |  |  | 3.3 | 2 |
| *YBR037C* | *SCO1* |  | 1.6 | 1.6 |  |  |  | 2 |
| *YMR294W-A* |  |  | 1.9 | 1.8 |  |  |  | 2 |
| *YHL039W* |  |  | 2 | 2.1 |  |  |  | 2 |
| *YDR401W* |  |  | 2.1 | 2.5 |  |  |  | 2 |
| *YBR175W* | *SWD3* |  | 2.5 | 2.8 |  |  |  | 2 |
| *YGR261C* | *APL6* |  | 2.8 | 3.9 |  |  |  | 2 |
| *YPL225W* |  |  |  | -1.6 |  |  | -3.2 | 2 |
| *YDR423C* | *CAD1* |  |  | 1.7 | 2.2 |  |  | 2 |
| *YGR263C* | *SAY1* |  |  | 2 | 2.8 |  |  | 2 |
| *YKR048C* | *NAP1* |  |  |  | -3.2 |  | -3.6 | 2 |
| *YBL047C* | *EDE1* |  |  |  | -2.8 | -2.8 |  | 2 |
| *YKR047W* |  |  |  |  | -2.7 | -3.25 |  | 2 |
| *YMR275C* | *BUL1* |  |  |  | -2.3 |  | -2.5 | 2 |
| *YHR206W* | *SKN7* |  |  |  | -2.1 | -4.3 |  | 2 |
| *YML131W* |  |  |  |  | 1.7 |  | 1.7 | 2 |
| *YDR319C* |  |  |  |  | 1.9 | 1.8 |  | 2 |
| *YDR326C* | *YSP2* |  |  |  | 2 | 1.9 |  | 2 |
| *YBR169C* | *SSE2* |  |  |  | 2 | 2 |  | 2 |
| *YNL010W* |  |  |  |  | 2.1 | 1.9 |  | 2 |
| *YLR443W* | *ECM7* |  |  |  | 2.2 | 2.3 |  | 2 |
| *YNL122C* |  |  |  |  | 2.2 | 2.3 |  | 2 |
| *YIR030C* | *DCG1* |  |  |  | 2.3 | 2.5 |  | 2 |
| *YBR186W* | *PCH2* |  |  |  | 2.3 |  | 2.2 | 2 |
| *YNL278W* | *CAF120* |  |  |  | 2.4 |  | 2.7 | 2 |
| *YKR088C* | *TVP38* |  |  |  | 2.4 |  | 2.1 | 2 |
| *YDL161W* | *ENT1* |  |  |  | 2.5 | 2.5 |  | 2 |
| *YLR327C* | *TMA10* |  |  |  | 2.5 | 2.8 |  | 2 |
| *YGR107W* |  |  |  |  | 2.5 | 2 |  | 2 |
| *YBL107C* |  |  |  |  | 2.6 |  | 2.3 | 2 |
| *YEL068C* |  |  |  |  | 2.6 |  | 2.8 | 2 |
| *YMR044W* | *IOC4* |  |  |  | 2.8 | 2.6 |  | 2 |
| *YGR230W* | *BNS1* |  |  |  | 2.9 | 3.5 |  | 2 |
| *YPL013C* | *MRPS16* |  |  |  | 2.9 | 2.5 |  | 2 |
| *YFL013W-A* |  |  |  |  | 3.3 |  | 3 | 2 |
| *YOR173W* | *DCS2* |  |  |  | 3.6 | 2.9 |  | 2 |
| *YBL062W* |  |  |  |  |  | -2.7 | -2.9 | 2 |
| *YLR422W* |  |  |  |  |  | 1.8 | 2.2 | 2 |
| *YGR271W* | *SLH1* |  |  |  |  | 2 | 1.9 | 2 |
| *YPR106W* | *ISR1* |  |  |  |  | 2.1 | 2.4 | 2 |
| *YBR019C* | *GAL10* |  |  |  |  | 2.8 | 3 | 2 |
| *YHR157W* | *REC104* |  |  |  |  | 4.1 | 4.4 | 2 |
| *YML079W* |  |  |  |  |  | 5.5 | 4.9 | 2 |
| *YLR111W* |  |  |  |  | -3.2 | -3.2 |  | 2 |
| *YER084W* |  |  |  |  | -2.2 |  | -3.3 | 2 |
| *YBR129C* | *OPY1* |  |  |  | 1.7 |  | 1.7 | 2 |
| *YMR284W* | *YKU70* |  |  |  | 1.9 | 2.1 |  | 2 |
| *YHR079C-B* |  |  |  |  | 2.3 | 2.4 |  | 2 |
| *YLR188W* | *MDL1* |  |  |  | 2.3 | 2.4 |  | 2 |
| *YDR221W* | *GTB1* |  |  |  | 2.4 | 2.2 |  | 2 |
| *YOR030W* | *DFG16* |  |  |  | 2.4 |  | 3 | 2 |
| *YPR140W* | *TAZ1* |  |  |  | 2.7 | 2.7 |  | 2 |
| *YDL070W* | *BDF2* |  |  |  | 2.9 |  | 2.6 | 2 |
| *YMR119W-A* |  |  |  |  | 3.2 | 3.1 |  | 2 |
| *YCR007C* |  |  |  |  | 4 | 3.9 |  | 2 |
| *YJL027C* |  |  |  |  |  | -2.7 | -2.4 | 2 |
| *YGR209C* | *TRX2* |  |  |  |  | -2.2 | -2.5 | 2 |
| *YMR101C* | *SRT1* |  |  |  |  | 1.7 | 2 | 2 |
| *YOR086C* | *TCB1* | -2.5 |  |  |  |  |  | 1 |
| *YOR014W* | *RTS1* | -2.3 |  |  |  |  |  | 1 |
| *YPR064W* |  | -1.8 |  |  |  |  |  | 1 |
| *YKL176C* | *LST4* | -1.5 |  |  |  |  |  | 1 |
| *YER067W* |  | -1.4 |  |  |  |  |  | 1 |
| *YKL097C* |  | -1.3 |  |  |  |  |  | 1 |
| *YBR292C* |  | -1.2 |  |  |  |  |  | 1 |
| *YMR307W* | *GAS1* | 1.5 |  |  |  |  |  | 1 |
| *YDR525W* | *API2* | 1.6 |  |  |  |  |  | 1 |
| *YDL158C* |  | 1.8 |  |  |  |  |  | 1 |
| *YCL055W* | *KAR4* | 2 |  |  |  |  |  | 1 |
| *YBR156C* | *SLI15* | 2.2 |  |  |  |  |  | 1 |
| *YMR250W* | *GAD1* | 2.6 |  |  |  |  |  | 1 |
| *YCL030C* | *HIS4* | 3.4 |  |  |  |  |  | 1 |
| *YGL203C* | *KEX1* | 4.3 |  |  |  |  |  | 1 |
| *YHR057C* | *CPR2* |  | -1.8 |  |  |  |  | 1 |
| *YOR082C* |  |  | -1.4 |  |  |  |  | 1 |
| *YMR157C* | *FMP39* |  | -1.3 |  |  |  |  | 1 |
| *YJR154W* |  |  | 1.3 |  |  |  |  | 1 |
| *YJR051W* | *OSM1* |  | 1.6 |  |  |  |  | 1 |
| *YLR262C* | *YPT6* |  | 1.9 |  |  |  |  | 1 |
| *YGL071W* | *AFT1* |  |  | -2.05 |  |  |  | 1 |
| *YPL161C* | *BEM4* |  |  | -2.1 |  |  |  | 1 |
| *YAL040C* | *CLN3* |  |  | -1.9 |  |  |  | 1 |
| *YKL080W* | *VMA5* |  |  | -1.9 |  |  |  | 1 |
| *YKL119C* | *VPH2* |  |  | -1.9 |  |  |  | 1 |
| *YLR451W* | *LEU3* |  |  | -1.65 |  |  |  | 1 |
| *YHR060W* | *VMA22* |  |  | -1.8 |  |  |  | 1 |
| *YDL173W* |  |  |  | -1.8 |  |  |  | 1 |
| *YDR359C* | *VID21* |  |  | -1.7 |  |  |  | 1 |
| *YPR153W* |  |  |  | -1.95 |  |  |  | 1 |
| *YDR484W* | *VPS52* |  |  | -2 |  |  |  | 1 |
| *YLR436C* | *ECM30* |  |  | -1.6 |  |  |  | 1 |
| *YHR015W* | *MIP6* |  |  | -1.6 |  |  |  | 1 |
| *YKR072C* | *SIS2* |  |  | -1.5 |  |  |  | 1 |
| *YBL051C* | *PIN4* |  |  | -1.5 |  |  |  | 1 |
| *YDR229W* | *IVY1* |  |  | -1.4 |  |  |  | 1 |
| *YAL011W* | *SWC3* |  |  | -1.4 |  |  |  | 1 |
| *YNL183C* | *NPR1* |  |  | -1.3 |  |  |  | 1 |
| *YMR194W* | *RPL36A* |  |  | -1.3 |  |  |  | 1 |
| *YCR049C* |  |  |  | -1.2 |  |  |  | 1 |
| *YOR324C* | *FRT1* |  |  | -1.2 |  |  |  | 1 |
| *YER156C* |  |  |  | -1.1 |  |  |  | 1 |
| *YKL197C* | *PEX1* |  |  | -1.1 |  |  |  | 1 |
| *YKL100C* |  |  |  | -1 |  |  |  | 1 |
| *YHR120W* | *MSH1* |  |  | 1.4 |  |  |  | 1 |
| *YDR065W* |  |  |  | 1.4 |  |  |  | 1 |
| *YDR520C* |  |  |  | 1.5 |  |  |  | 1 |
| *YJR105W* | *ADO1* |  |  | 1.5 |  |  |  | 1 |
| *YOR138C* | *RUP1* |  |  | 1.6 |  |  |  | 1 |
| *YCL036W* | *GFD2* |  |  | 1.6 |  |  |  | 1 |
| *YML073C* | *RPL6A* |  |  | 1.7 |  |  |  | 1 |
| *YOR161C* | *PNS1* |  |  | 1.7 |  |  |  | 1 |
| *YCL056C* |  |  |  | 1.8 |  |  |  | 1 |
| *YBR159W* | *IFA38* |  |  | 1.8 |  |  |  | 1 |
| *YGL214W* |  |  |  | 1.9 |  |  |  | 1 |
| *YNR005C* |  |  |  | 1.9 |  |  |  | 1 |
| *YGR183C* | *QCR9* |  |  | 2 |  |  |  | 1 |
| *YMR286W* | *MRPL33* |  |  | 2.1 |  |  |  | 1 |
| *YGL087C* | *MMS2* |  |  | 2.1 |  |  |  | 1 |
| *YLR062C* | *BUD28* |  |  | 2.2 |  |  |  | 1 |
| *YOR021C* |  |  |  | 2.3 |  |  |  | 1 |
| *YER051W* | *JHD1* |  |  | 2.4 |  |  |  | 1 |
| *YDR456W* | *NHX1* |  |  | 2.6 |  |  |  | 1 |
| *YER047C* | *SAP1* |  |  | 2.7 |  |  |  | 1 |
| *YNL326C* | *PFA3* | 1.7 |  |  |  |  |  | 1 |
| *YCR044C* | *PER1* | 2 |  |  |  |  |  | 1 |
| *YHR037W* | *PUT2* | 2.1 |  |  |  |  |  | 1 |
| *YNL236W* | *SIN4* | 2.4 |  |  |  |  |  | 1 |
| *YGL038C* | *OCH1* | 2.5 |  |  |  |  |  | 1 |
| *YEL036C* | *ANP1* | 2.5 |  |  |  |  |  | 1 |
| *YLL057C* | *JLP1* | 2.6 |  |  |  |  |  | 1 |
| *YGL179C* | *TOS3* | 3.9 |  |  |  |  |  | 1 |
| *YKL135C* | *APL2* | 4 |  |  |  |  |  | 1 |
| *YDL117W* | *CYK3* |  | 1.7 |  |  |  |  | 1 |
| *YDR074W* | *TPS2* |  |  | -2.8 |  |  |  | 1 |
| *YCR053W* | *THR4* |  |  | -2.4 |  |  |  | 1 |
| *YDR392W* | *SPT3* |  |  | -2.3 |  |  |  | 1 |
| *YKL098W* |  |  |  | -2.3 |  |  |  | 1 |
| *YGR057C* | *LST7* |  |  | -2.3 |  |  |  | 1 |
| *YDR162C* | *NBP2* |  |  | -2.2 |  |  |  | 1 |
| *YBR197C* |  |  |  | -2.1 |  |  |  | 1 |
| *YHR178W* | *STB5* |  |  | -1.9 |  |  |  | 1 |
| *YLR315W* | *NKP2* |  |  | -1.8 |  |  |  | 1 |
| *YFL023W* | *BUD27* |  |  | -1.8 |  |  |  | 1 |
| *YNL229C* | *URE2* |  |  | -1.7 |  |  |  | 1 |
| *YHR028C* | *DAP2* |  |  | -1.6 |  |  |  | 1 |
| *YNL056W* | *OCA2* |  |  | -1.6 |  |  |  | 1 |
| *YHR132C* | *ECM14* |  |  | -1.5 |  |  |  | 1 |
| *YNL032W* | *SIW14* |  |  | -1.5 |  |  |  | 1 |
| *YLR414C* |  |  |  | -1.2 |  |  |  | 1 |
| *YHR104W* | *GRE3* |  |  | -1.2 |  |  |  | 1 |
| *YMR205C* | *PFK2* |  |  | 1.3 |  |  |  | 1 |
| *YPL213W* | *LEA1* |  |  | 1.6 |  |  |  | 1 |
| *YMR075W* | *RCO1* |  |  | 1.9 |  |  |  | 1 |
| *YCR027C* | *RHB1* |  |  | 2 |  |  |  | 1 |
| *YLL006W* | *MMM1* |  |  | 2.1 |  |  |  | 1 |
| *YLR133W* | *CKI1* |  |  | 2.1 |  |  |  | 1 |
| *YGL058W* | *RAD6* |  |  | 2.2 |  |  |  | 1 |
| *YHR159W* |  |  |  | 2.3 |  |  |  | 1 |
| *YMR166C* |  |  |  | 2.6 |  |  |  | 1 |
| *YDR431W* |  |  |  |  | -3.3 |  |  | 1 |
| *YGL164C* | *YRB30* |  |  |  | -2.2 |  |  | 1 |
| *YDL018C* | *ERP3* |  |  |  | -2 |  |  | 1 |
| *YLR380W* | *CSR1* |  |  |  | -2 |  |  | 1 |
| *YJR019C* | *TES1* |  |  |  | -1.8 |  |  | 1 |
| *YMR019W* | *STB4* |  |  |  | -1.7 |  |  | 1 |
| *YCR106W* | *RDS1* |  |  |  | -1.5 |  |  | 1 |
| *YDL242W* |  |  |  |  | -1.4 |  |  | 1 |
| *YIR031C* | *DAL7* |  |  |  | 1.7 |  |  | 1 |
| *YHR153C* | *SPO16* |  |  |  | 1.8 |  |  | 1 |
| *YMR244W* |  |  |  |  | 1.9 |  |  | 1 |
| *YDR402C* | *DIT2* |  |  |  | 1.9 |  |  | 1 |
| *YBR164C* | *ARL1* |  |  |  | 1.9 |  |  | 1 |
| *YHR185C* | *PFS1* |  |  |  | 2 |  |  | 1 |
| *YBR071W* |  |  |  |  | 2.1 |  |  | 1 |
| *YDL135C* | *RDI1* |  |  |  | 2.2 |  |  | 1 |
| *YDR409W* | *SIZ1* |  |  |  | 2.3 |  |  | 1 |
| *YOR137C* | *SIA1* |  |  |  | 2.3 |  |  | 1 |
| *YNL130C* | *CPT1* |  |  |  | 2.4 |  |  | 1 |
| *YDR322W* | *MRPL35* |  |  |  | 2.4 |  |  | 1 |
| *YMR175W* | *SIP18* |  |  |  | 2.5 |  |  | 1 |
| *YHR177W* |  |  |  |  | 2.5 |  |  | 1 |
| *YBR114W* | *RAD16* |  |  |  | 2.6 |  |  | 1 |
| *YDR305C* | *HNT2* |  |  |  | 2.7 |  |  | 1 |
| *YFL025C* | *BST1* |  |  |  | 2.9 |  |  | 1 |
| *YKL101W* | *HSL1* |  |  |  |  | -2.75 |  | 1 |
| *YML097C* | *VPS9* |  |  |  |  | -2.7 |  | 1 |
| *YDR507C* | *GIN4* |  |  |  |  | -2.4 |  | 1 |
| *YER098W* | *UBP9* |  |  |  |  | -1.6 |  | 1 |
| *YPR179C* | *HDA3* |  |  |  |  | 1.6 |  | 1 |
| *YML022W* | *APT1* |  |  |  |  | 1.7 |  | 1 |
| *YJL048C* | *UBX6* |  |  |  |  | 1.8 |  | 1 |
| *YPL015C* | *HST2* |  |  |  |  | 1.9 |  | 1 |
| *YBL106C* | *SRO77* |  |  |  |  | 2.3 |  | 1 |
| *YOR167C* | *RPS28A* |  |  |  |  | 2.3 |  | 1 |
| *YPR201W* | *ARR3* |  |  |  |  | 2.4 |  | 1 |
| *YGR192C* | *TDH3* |  |  |  |  | 2.6 |  | 1 |
| *YFL044C* | *OTU1* |  |  |  |  | 2.6 |  | 1 |
| *YOR216C* | *RUD3* |  |  |  |  |  | -3.15 | 1 |
| *YER139C* |  |  |  |  |  |  | -3.5 | 1 |
| *YGL147C* | *RPL9A* |  |  |  |  |  | -3.4 | 1 |
| *YAR003W* | *SWD1* |  |  |  |  |  | -3.4 | 1 |
| *YNL239W* | *LAP3* |  |  |  |  |  | -2.55 | 1 |
| *YHR129C* | *ARP1* |  |  |  |  |  | -2.9 | 1 |
| *YKL205W* | *LOS1* |  |  |  |  |  | -2.6 | 1 |
| *YPR093C* | *ASR1* |  |  |  |  |  | -2.5 | 1 |
| *YJL132W* |  |  |  |  |  |  | -2.5 | 1 |
| *YBL080C* | *PET112* |  |  |  |  |  | -2.3 | 1 |
| *YLR023C* | *IZH3* |  |  |  |  |  | -1.6 | 1 |
| *YDR250C* |  |  |  |  |  |  | 1.6 | 1 |
| *YGR144W* | *THI4* |  |  |  |  |  | 1.6 | 1 |
| *YLR356W* |  |  |  |  |  |  | 1.8 | 1 |
| *YMR003W* |  |  |  |  |  |  | 1.8 | 1 |
| *YOR275C* | *RIM20* |  |  |  |  |  | 1.9 | 1 |
| *YFL010C* | *WWM1* |  |  |  |  |  | 1.9 | 1 |
| *YMR154C* | *RIM13* |  |  |  |  |  | 1.9 | 1 |
| *YJR050W* | *ISY1* |  |  |  |  |  | 2 | 1 |
| *YMR219W* | *ESC1* |  |  |  |  |  | 2.5 | 1 |
| *YFL012W* |  |  |  |  |  |  | 2.6 | 1 |
| *YPL027W* | *SMA1* |  |  |  |  |  | 2.7 | 1 |
| *YGR122W* |  |  |  |  |  |  | 2.75 | 1 |
| *YDL073W* |  |  |  |  |  |  | 2.9 | 1 |
| *YJL099W* | *CHS6* |  |  |  | -2.8 |  |  | 1 |
| *YNL079C* | *TPM1* |  |  |  | -2.7 |  |  | 1 |
| *YLR426W* |  |  |  |  | -2.7 |  |  | 1 |
| *YPL066W* |  |  |  |  | -2.4 |  |  | 1 |
| *YMR153W* | *NUP53* |  |  |  | -2.3 |  |  | 1 |
| *YKL123W* |  |  |  |  | -2.2 |  |  | 1 |
| *YJL204C* | *RCY1* |  |  |  | -2.2 |  |  | 1 |
| *YCL050C* | *APA1* |  |  |  | -1.9 |  |  | 1 |
| *YKR033C* |  |  |  |  | -1.8 |  |  | 1 |
| *YDL236W* | *PHO13* |  |  |  | -1.8 |  |  | 1 |
| *YHL026C* |  |  |  |  | -1.7 |  |  | 1 |
| *YKL071W* |  |  |  |  | -1.7 |  |  | 1 |
| *YMR311C* | *GLC8* |  |  |  | 1.7 |  |  | 1 |
| *YLR036C* |  |  |  |  | 1.7 |  |  | 1 |
| *YBR006W* | *UGA2* |  |  |  | 1.8 |  |  | 1 |
| *YGL242C* |  |  |  |  | 1.9 |  |  | 1 |
| *YFR026C* |  |  |  |  | 2 |  |  | 1 |
| *YMR262W* |  |  |  |  | 2 |  |  | 1 |
| *YDR220C* |  |  |  |  | 2 |  |  | 1 |
| *YLR341W* | *SPO77* |  |  |  | 2.2 |  |  | 1 |
| *YDR414C* | *ERD1* |  |  |  | 2.5 |  |  | 1 |
| *YGR242W* |  |  |  |  | 2.6 |  |  | 1 |
| *YDR385W* | *EFT2* |  |  |  | 2.6 |  |  | 1 |
| *YCL005W* | *LDB16* |  |  |  | 2.7 |  |  | 1 |
| *YNL101W* | *AVT4* |  |  |  | 2.7 |  |  | 1 |
| *YKL133C* |  |  |  |  | 2.8 |  |  | 1 |
| *YIL009C-A* | *EST3* |  |  |  | 3.1 |  |  | 1 |
| *YKR041W* |  |  |  |  | 3.4 |  |  | 1 |
| *YLR352W* |  |  |  |  |  | -4.1 |  | 1 |
| *YLR020C* | *YEH2* |  |  |  |  | -2 |  | 1 |
| *YLR265C* | *NEJ1* |  |  |  |  | 1.8 |  | 1 |
| *YDR306C* |  |  |  |  |  | 2 |  | 1 |
| *YGR038W* | *ORM1* |  |  |  |  | 2.2 |  | 1 |
| *YLR304C* | *ACO1* |  |  |  |  | 2.5 |  | 1 |
| *YDR389W* | *SAC7* |  |  |  |  | 2.7 |  | 1 |
| *YDR388W* | *RVS167* |  |  |  |  | 2.9 |  | 1 |
| *YLR061W* | *RPL22A* |  |  |  |  | 3 |  | 1 |
| *YKL160W* | *ELF1* |  |  |  |  |  | -4.6 | 1 |
| *YKR042W* | *UTH1* |  |  |  |  |  | -3.9 | 1 |
| *YOR089C* | *VPS21* |  |  |  |  |  | -3.8 | 1 |
| *YIR037W* | *HYR1* |  |  |  |  |  | -3.6 | 1 |
| *YGR135W* | *PRE9* |  |  |  |  |  | -3.5 | 1 |
| *YPL102C* |  |  |  |  |  |  | -3.1 | 1 |
| *YMR274C* | *RCE1* |  |  |  |  |  | -2.3 | 1 |
| *YDR247W* | *VHS1* |  |  |  |  |  | -2.1 | 1 |
| *YKL076C* | *PSY1* |  |  |  |  |  | -2.1 | 1 |
| *YCL045C* |  |  |  |  |  |  | -2 | 1 |
| *YMR264W* | *CUE1* |  |  |  |  |  | -1.9 | 1 |
| *YKL027W* |  |  |  |  |  |  | -1.8 | 1 |
| *YNL289W* | *PCL1* |  |  |  |  |  | -1.8 | 1 |
| *YGR124W* | *ASN2* |  |  |  |  |  | -1.8 | 1 |
| *YNL253W* | *TEX1* |  |  |  |  |  | -1.6 | 1 |
| *YER118C* | *SHO1* |  |  |  |  |  | -1.6 | 1 |
| *YOR005C* | *DNL4* |  |  |  |  |  | -1.5 | 1 |
| *YIR013C* | *GAT4* |  |  |  |  |  | -1.4 | 1 |
| *YPL022W* | *RAD1* |  |  |  |  |  | 1.5 | 1 |
| *YPL057C* | *SUR1* |  |  |  |  |  | 1.6 | 1 |
| *YPL183C* |  |  |  |  |  |  | 1.8 | 1 |
| *YPR012W* |  |  |  |  |  |  | 1.8 | 1 |
| *YJR010W* | *MET3* |  |  |  |  |  | 1.9 | 1 |
| *YJR004C* | *SAG1* |  |  |  |  |  | 2 | 1 |
| *YHL027W* | *RIM101* |  |  |  |  |  | 2 | 1 |
| *YBL079W* | *NUP170* |  |  |  |  |  | 2.1 | 1 |
| *YDR403W* | *DIT1* |  |  |  |  |  | 2.1 | 1 |
| *YNL294C* | *RIM21* |  |  |  |  |  | 2.4 | 1 |
| *YDR384C* | *ATO3* |  |  |  |  |  | 2.5 | 1 |
| *YPL065W* | *VPS28* |  |  |  |  |  | 2.5 | 1 |
| *YLR373C* | *VID22* |  |  |  |  |  | 2.7 | 1 |
| *YOR235W* | *IRC13* |  |  |  |  |  | 2.9 | 1 |
| *YMR064W* | *AEP1* |  |  |  |  |  | 3.1 | 1 |
| *YOR078W* | *BUD21* |  |  |  |  |  | 3.5 | 1 |
| *YMR216C* | *SKY1* |  |  |  |  |  | 3.7 | 1 |
