# Supplementary material for: Genome-Wide Functional Profiling Reveals Genes Required for Tolerance to Benzene Metabolites in Yeast
Source: PLoS One. 2011 Aug 30;6(8):e24205. doi: 10.1371/journal.pone.0024205 (PMC3166172; doi:10.1371/journal.pone.0024205)
Supplement: Table S2 — Complete list of yeast genes (n = 528) identified by DSSA after treatment with catechol (CAT), ranked by the number of hits in 6 treatments. Yeast pools were exposed to 3 different concentrations of catechol for two generation-points, for a total of 6 treatments. The yeast ORFs/genes correspond to deletion strains that exhibited a significant change in growth in at least one treatment with catechol (q<0.05). Numeric values are fitness scores (log2 ratios) calculated only for significant genes in each individual treatment. Empty cells indicate that the gene was not significant in that particular treatment. (DOC) [file pone.0024205.s008.doc]

**Table S2. Complete list of yeast genes (n = 528) identified by DSSA after treatment with catechol (CAT), ranked by the number of hits in 6 treatments.** Yeast pools were exposed to 3 different concentrations of catechol for two generation-points, for a total of 6 treatments. The yeast ORFs / genes correspond to deletion strains that exhibited a significant change in growth in at least one treatment with catechol (q < 0.05). Numeric values are fitness scores (log_2_ ratios) calculated only for significant genes in each individual treatment. Empty cells indicate that the gene was not significant in that particular treatment.

|  |  | 5 generations | | | 15 generations | | |  |
| --- | --- | --- | --- | --- | --- | --- | --- | --- |
| ORF | Gene | 25% IC_20_ | 50% IC_20_ | IC_20_ | 25% IC_20_ | 50% IC_20_ | IC_20_ | # of hits |
|  |  | 0.55mM | 1.1mM | 2.2mM | 0.55mM | 1.1mM | 2.2mM |  |
| *YMR286W* | *MRPL33* | 1.8 | 1.8 | 1.9 | 2.7 | 2 | 2.5 | 6 |
| *YOR314W* |  | 2.3 | 2.3 | 2.3 | 2.6 | 2.2 | 2.3 | 6 |
| *YOR021C* |  | 2.6 | 3 | 3 | 4.3 | 2.1 | 2 | 6 |
| *YPL157W* | *TGS1* | 3.25 | 3.35 | 3.5 | 3.7 | 2.8 | 2.8 | 6 |
| *YJR088C* |  | 3.3 | 3.2 | 3.3 | 3.1 | 2.4 | 2.6 | 6 |
| *YDR179C* | *CSN9* | 2.8 | 2.6 | 2.7 | -1.9 | -1.6 | -1.7 | 6 |
| *YDR458C* | *HEH2* | 2.9 | 3.05 | 2.9 | 3.4 | 2.8 | 2.8 | 6 |
| *YDL223C* | *HBT1* | 2.25 | 2.4 | 2.35 | 2.3 | 2.2 | 2.2 | 6 |
| *YKL174C* | *TPO5* | 1.9 | 2.1 | 2.2 | 5.5 | 5.1 | 4.8 | 6 |
| *YBR037C* | *SCO1* | 2 | 2.2 | 1.8 | 2.3 | 2.4 | 2.3 | 6 |
| *YLR335W* | *NUP2* | 2.2 | 2.4 | 2.5 | 2.3 | 1.8 | 2 | 6 |
| *YNL101W* | *AVT4* | 2.4 | 2.7 | 2.7 | 2.7 | 2.5 | 2.3 | 6 |
| *YGR263C* | *SAY1* | 2.4 | 2.7 | 2.6 | 2.5 | 2.2 | 2.5 | 6 |
| *YDR153C* | *ENT5* | 3.2 | 3.5 | 3.2 | 3.1 | 2.4 | 2.6 | 6 |
| *YOR133W* | *EFT1* | 3.4 | 3.7 | 3.6 | 3.4 | 2.4 | 2.7 | 6 |
| *YCR073W-A* | *SOL2* | 3 | 3.1 | 3.1 | 2.9 | 2.1 |  | 5 |
| *YER169W* | *RPH1* | 3.1 | 3 | 3.4 |  | 2 | 2.1 | 5 |
| *YBR044C* | *TCM62* | 1.8 |  | 1.8 | 3 | 2.3 | 2.6 | 5 |
| *YGR130C* |  | -2.3 | -2.2 | -2.3 |  | -1.9 | -1.8 | 5 |
| *YIR028W* | *DAL4* | 3.2 | 3.3 | 3.1 | 3.1 | 2.6 |  | 5 |
| *YGL242C* |  |  | 1.5 | 1.4 | 1.7 | 1.4 | 1.5 | 5 |
| *YNL264C* | *PDR17* |  | 2 | 1.8 | 2.4 | 2.6 | 2.5 | 5 |
| *YMR262W* |  |  | 2 | 2.1 | 2.3 | 1.8 | 1.7 | 5 |
| *YER066C-A* |  | 2.2 | 2.4 | 2.4 | 3.5 |  |  | 4 |
| *YIL139C* | *REV7* | 2.1 | 2.2 | 2.1 |  | 2 |  | 4 |
| *YOR292C* |  | 1.7 | 1.9 | 2 | 2.9 |  |  | 4 |
| *YJL131C* |  | 2.1 | 2.1 | 2.3 | 3.6 |  |  | 4 |
| *YMR304W* | *UBP15* | 3.4 | 3.4 | 3.4 |  |  | 2 | 4 |
| *YOR352W* |  | 2.3 | 2.2 | 2.2 | -1.5 |  |  | 4 |
| *YBR187W* | *GDT1* | 4.3 | 4.6 | 4.5 | 2.8 |  |  | 4 |
| *YPR076W* |  | -1.8 | -1.8 | -1.7 | -2.9 |  |  | 4 |
| *YCL046W* |  | 1.9 | 1.8 | 1.8 |  | 1.8 |  | 4 |
| *YCR011C* | *ADP1* | 1.7 | 1.7 | 1.8 |  | 1.7 |  | 4 |
| *YGR117C* |  | -2 | -2 | -1.9 | -3.1 |  |  | 4 |
| *YDR105C* | *TMS1* | 2.7 | 2.9 | 2.9 | 3 |  |  | 4 |
| *YDR314C* | *RAD34* | 1.7 | 1.8 | 1.9 | 2 |  |  | 4 |
| *YDR456W* | *NHX1* | 2.5 | 2.8 | 2.7 |  | 1.7 |  | 4 |
| *YKR046C* | *PET10* | -1.4 | -1.4 | -1.3 | -4 |  |  | 4 |
| *YDL211C* |  | -1.2 | -1.3 | -1.1 | -4 |  |  | 4 |
| *YGL138C* |  | 2.6 | 3 | 3.2 |  |  | 2.1 | 4 |
| *YER167W* | *BCK2* |  | 1.3 | 1.2 |  | 1.2 | 1.7 | 4 |
| *YAL002W* | *VPS8* |  |  | -2.3 | -3.5 | -3.6 | -5 | 4 |
| *YJL120W* |  |  |  | -1.5 | -2.75 | -2.95 | -4 | 4 |
| *YKL133C* |  |  |  | -1.2 | 2.8 | 2.6 | 2.6 | 4 |
| *YJL121C* | *RPE1* |  |  | -1.1 | -2.9 | -2.6 | -3.8 | 4 |
| *YGR034W* | *RPL26B* | 1.7 | 1.6 | 1.8 |  | 1.9 |  | 4 |
| *YMR103C* |  | 1.8 | 2.1 | 2.1 | 2.2 |  |  | 4 |
| *YGR070W* | *ROM1* | 1.9 | 2.4 | 2.3 | 2.6 |  |  | 4 |
| *YMR244C-A* |  | 2.1 | 2.4 | 2.2 | 2.4 |  |  | 4 |
| *YMR184W* | *ADD37* | 2.1 | 2.1 | 2.1 | 2.4 |  |  | 4 |
| *YBR170C* | *NPL4* | 2.3 | 2.5 | 2.3 | 3.3 |  |  | 4 |
| *YFR018C* |  | 2.4 | 2.6 | 2.8 | 2.7 |  |  | 4 |
| *YKR035C* | *OPI8* | 2.7 | 2.7 | 2.7 | 2.1 |  |  | 4 |
| *YPL035C* |  | 2.7 | 2.9 | 2.9 | 3.2 |  |  | 4 |
| *YBR175W* | *SWD3* | 2.8 | 2.9 | 3 | 2.7 |  |  | 4 |
| *YMR280C* | *CAT8* | 2.9 | 3 | 3 | 2.2 |  |  | 4 |
| *YGL118C* |  | 3.1 | 3.3 | 3 | 2.2 |  |  | 4 |
| *YDR423C* | *CAD1* |  | 1.4 | 1.6 | 1.8 | 1.8 |  | 4 |
| *YJR044C* | *VPS55* | 2.6 | 2.7 | 2.5 |  |  |  | 3 |
| *YIL153W* | *RRD1* | 2 | 2.1 | 1.8 |  |  |  | 3 |
| *YOR084W* |  | -1.2 | -1.1 | -1.1 |  |  |  | 3 |
| *YJL208C* | *NUC1* | 1.4 | 1.2 | 1.3 |  |  |  | 3 |
| *YLR450W* | *HMG2* | 2.8 | 2.7 | 3 |  |  |  | 3 |
| *YLR297W* |  | 1.4 | 1.8 | 1.8 |  |  |  | 3 |
| *YOR019W* |  | 1.9 | 1.8 | 1.8 |  |  |  | 3 |
| *YPL230W* |  | -1.2 | -1.2 | -1.1 |  |  |  | 3 |
| *YJR052W* | *RAD7* | 1.2 | 1.2 | 1.2 |  |  |  | 3 |
| *YEL068C* |  | 1.5 |  | 1.5 | 3 |  |  | 3 |
| *YML048W-A* |  | 1.4 | 1.6 | 1.6 |  |  |  | 3 |
| *YJL070C* |  | 1.2 | 1.3 | 1.3 |  |  |  | 3 |
| *YMR261C* | *TPS3* | 2.1 | 2.3 | 2.3 |  |  |  | 3 |
| *YMR256C* | *COX7* | 2.3 | 2.5 | 2.3 |  |  |  | 3 |
| *YHR060W* | *VMA22* | -1.4 | -2.05 | -2.45 |  |  |  | 3 |
| *YER047C* | *SAP1* | 2.7 | 2.5 | 2.8 |  |  |  | 3 |
| *YHR046C* | *INM1* | 1.8 | 2 | 2.2 |  |  |  | 3 |
| *YHR137W* | *ARO9* | -2 | -2 | -1.9 |  |  |  | 3 |
| *YER051W* | *JHD1* | 2.4 | 2.4 | 2.5 |  |  |  | 3 |
| *YOR161C* | *PNS1* | 1.9 | 2.2 | 2.2 |  |  |  | 3 |
| *YER002W* | *NOP16* | 2.7 | 2.6 | 2.7 |  |  |  | 3 |
| *YKL166C* | *TPK3* | 2.1 | 2 | 2.2 |  |  |  | 3 |
| *YBR298C* | *MAL31* | 2.3 | 2.6 | 2.3 |  |  |  | 3 |
| *YJL127C* | *SPT10* | 1.9 | 1.6 | 1.7 |  |  |  | 3 |
| *YMR272C* | *SCS7* | 2 | 1.7 | 1.7 |  |  |  | 3 |
| *YGR183C* | *QCR9* | 2 | 1.5 |  |  | 1.8 |  | 3 |
| *YGR192C* | *TDH3* | 1.8 | 1.6 | 1.6 |  |  |  | 3 |
| *YBR114W* | *RAD16* | 1.9 | 2.1 | 2.1 |  |  |  | 3 |
| *YDL182W* | *LYS20* | -1.7 | -1.9 | -1.8 |  |  |  | 3 |
| *YDR465C* | *RMT2* | 1.9 | 2 | 2 |  |  |  | 3 |
| *YBR213W* | *MET8* | 2.8 | 2.8 | 2.8 |  |  |  | 3 |
| *YDL216C* | *RRI1* | 1.7 | 1.6 | 1.6 |  |  |  | 3 |
| *YNR005C* |  | 1.7 | 1.7 | 1.6 |  |  |  | 3 |
| *YLR121C* | *YPS3* | 1.8 | 1.8 | 1.7 |  |  |  | 3 |
| *YPR049C* | *ATG11* | 1.4 | 1.5 | 1.5 |  |  |  | 3 |
| *YOR199W* |  | 2.4 | 2.3 | 2.3 |  |  |  | 3 |
| *YLR073C* |  | 1.6 | 1.8 | 1.9 |  |  |  | 3 |
| *YCR001W* |  | 1.7 | 1.7 | 1.8 |  |  |  | 3 |
| *YCR006C* |  | 3 | 3.2 | 3.2 |  |  |  | 3 |
| *YCL036W* | *GFD2* | 1.7 | 1.8 | 1.7 |  |  |  | 3 |
| *YDR377W* | *ATP17* | 1.7 | 1.6 | 1.5 |  |  |  | 3 |
| *YDR163W* | *CWC15* | 1.7 | 1.8 | 1.7 |  |  |  | 3 |
| *YDR283C* | *GCN2* | 2.2 | 2 | 2.1 |  |  |  | 3 |
| *YDR441C* | *APT2* | 1.5 | 1.7 | 1.6 |  |  |  | 3 |
| *YDR175C* | *RSM24* | 2.1 | 1.8 | 1.8 |  |  |  | 3 |
| *YGL043W* | *DST1* | -1.6 | -1.6 | -1.4 |  |  |  | 3 |
| *YGL071W* | *AFT1* | -1.4 | -2.65 | -3.8 |  |  |  | 3 |
| *YGL041C* |  | 1.6 | 1.8 | 1.7 |  |  |  | 3 |
| *YLR169W* |  | 1.9 | 1.8 | 1.8 |  |  |  | 3 |
| *YDR520C* |  | 1.7 | 1.9 | 1.8 |  |  |  | 3 |
| *YDR226W* | *ADK1* | 2.3 | 2 | 1.9 |  |  |  | 3 |
| *YOL013W-A* |  | 1.9 | 1.7 | 1.7 |  |  |  | 3 |
| *YGL087C* | *MMS2* | 2.3 | 2.4 | 2.4 |  |  |  | 3 |
| *YDR025W* | *RPS11A* | 2.3 | 2.1 | 2.1 |  |  |  | 3 |
| *YGL229C* | *SAP4* | 2 | 1.9 | 2 |  |  |  | 3 |
| *YDL213C* | *NOP6* | 1.6 | 1.8 | 1.8 |  |  |  | 3 |
| *YDL204W* | *RTN2* | 1.4 | 1.3 | 1.2 |  |  |  | 3 |
| *YGL226C-A* | *OST5* | 2.9 | 2.9 | 3.1 |  |  |  | 3 |
| *YGL175C* | *SAE2* | 1.6 | 1.7 | 1.7 |  |  |  | 3 |
| *YGR041W* | *BUD9* | 2.9 | 3 | 2.9 |  |  |  | 3 |
| *YKL119C* | *VPH2* | -2 | -2.85 | -2.4 |  |  |  | 3 |
| *YNL187W* |  |  | 1.5 | 1.5 | 3.1 |  |  | 3 |
| *YOR044W* | *IRC23* |  | 1.7 |  |  | 1.5 | 1.6 | 3 |
| *YOR089C* | *VPS21* |  |  | -1.5 |  | -2.45 | -4.7 | 3 |
| *YLR202C* |  |  |  | 1.6 |  | 1.6 | 1.7 | 3 |
| *YCL007C* |  | -2.1 | -2.2 | -2.7 |  |  |  | 3 |
| *YOR331C* |  | -2.1 | -2.2 | -2.1 |  |  |  | 3 |
| *YJR135C* | *MCM22* | 1.4 | 1.6 | 1.7 |  |  |  | 3 |
| *YGR035C* |  | 1.4 |  | 1.5 |  | 1.6 |  | 3 |
| *YLR421C* | *RPN13* | 1.5 | 1.7 | 1.7 |  |  |  | 3 |
| *YGL004C* | *RPN14* | 1.6 | 1.9 | 1.6 |  |  |  | 3 |
| *YMR294W-A* |  | 1.7 |  | 2 | 2.1 |  |  | 3 |
| *YMR075W* | *RCO1* | 1.7 | 1.9 | 1.9 |  |  |  | 3 |
| *YCR027C* | *RHB1* | 1.7 | 1.8 | 1.9 |  |  |  | 3 |
| *YDR159W* | *SAC3* | 1.7 | 2.2 | 1.9 |  |  |  | 3 |
| *YOR201C* | *MRM1* | 1.8 | 1.7 | 1.5 |  |  |  | 3 |
| *YBL062W* |  | 1.8 | 1.9 | 1.9 |  |  |  | 3 |
| *YHL039W* |  | 1.8 | 2.1 | 2.2 |  |  |  | 3 |
| *YJR010C-A* | *SPC1* | 1.9 | 2 | 1.9 |  |  |  | 3 |
| *YHR182W* |  | 2 | 2.2 | 2.2 |  |  |  | 3 |
| *YOR364W* |  | 2 | 1.9 | 2.2 |  |  |  | 3 |
| *YDR401W* |  | 2 | 2.4 | 2.2 |  |  |  | 3 |
| *YHR037W* | *PUT2* | 2.1 | 2.4 | 2.1 |  |  |  | 3 |
| *YLR133W* | *CKI1* | 2.2 | 2.6 | 2.6 |  |  |  | 3 |
| *YMR166C* |  | 2.3 | 2.6 | 2.6 |  |  |  | 3 |
| *YHR159W* |  | 2.5 | 2.9 | 2.8 |  |  |  | 3 |
| *YPL178W* | *CBC2* | 2.8 | 3.1 | 3.1 |  |  |  | 3 |
| *YML070W* | *DAK1* | 3 | 3 | 3.4 |  |  |  | 3 |
| *YOR106W* | *VAM3* | 3 | 2.9 | 2 |  |  |  | 3 |
| *YEL012W* | *UBC8* | 3.4 | 3.7 | 3.8 |  |  |  | 3 |
| *YGR261C* | *APL6* | 3.4 | 3.7 | 3.4 |  |  |  | 3 |
| *YIL009C-A* | *EST3* |  |  | 1.4 | 2.7 | 2.1 |  | 3 |
| *YKR052C* | *MRS4* |  |  |  | -2.45 | -4.35 | -5.7 | 3 |
| *YMR153W* | *NUP53* |  |  |  | -0.45 | -2.2 | -2 | 3 |
| *YMR284W* | *YKU70* |  |  |  | 2.75 | 2 | 2 | 3 |
| *YFR036W* | *CDC26* |  |  |  | -5.2 | -4.95 | -4.75 | 3 |
| *YKR020W* | *VPS51* |  |  |  | -2.3 | -2.4 | -2.95 | 3 |
| *YKL071W* |  |  |  |  | -1.6 | -1.3 | -1.3 | 3 |
| *YKL123W* |  |  |  |  | -2.2 | -1.6 | -1.7 | 3 |
| *YKL222C* |  |  |  |  | -2.2 | -1.9 | -2 | 3 |
| *YGL094C* | *PAN2* |  |  |  | -2.1 | -2.1 | -2.2 | 3 |
| *YGR110W* |  |  |  |  | -1.9 | -1.8 | -1.9 | 3 |
| *YDL236W* | *PHO13* |  |  |  | -1.8 | -1.6 | -1.7 | 3 |
| *YKL051W* | *SFK1* |  |  |  | -1.8 | -1.7 | -1.8 | 3 |
| *YKR033C* |  |  |  |  | -1.8 | -1.5 | -1.6 | 3 |
| *YHL026C* |  |  |  |  | -1.7 | -1.4 | -1.5 | 3 |
| *YLR286C* | *CTS1* |  |  |  | 1.6 | 1.5 | 1.8 | 3 |
| *YDR124W* |  |  |  |  | 1.6 | 1.6 | 1.7 | 3 |
| *YML117W-A* |  |  |  |  | 1.8 | 1.5 | 1.6 | 3 |
| *YBR006W* | *UGA2* |  |  |  | 1.9 | 1.6 | 1.6 | 3 |
| *YFR026C* |  |  |  |  | 1.9 | 1.7 | 1.8 | 3 |
| *YDR220C* |  |  |  |  | 2 | 1.4 | 1.5 | 3 |
| *YDR221W* | *GTB1* |  |  |  | 2.2 | 1.8 | 2 | 3 |
| *YDR400W* | *URH1* |  |  |  | 2.2 | 1.8 | 1.7 | 3 |
| *YDL070W* | *BDF2* |  |  |  | 2.2 | 1.7 | 2.5 | 3 |
| *YMR299C* | *DYN3* |  |  |  | 2.3 | 2.2 | 2.3 | 3 |
| *YHR153C* | *SPO16* |  |  |  | 2.3 | 1.9 | 2 | 3 |
| *YPR140W* | *TAZ1* |  |  |  | 2.5 | 2.1 | 2.1 | 3 |
| *YBR149W* | *ARA1* |  |  |  | 2.5 | 2.3 | 2.3 | 3 |
| *YGR157W* | *CHO2* |  |  |  | 2.7 | 2 | 2.5 | 3 |
| *YKL096W-A* | *CWP2* |  |  |  | 2.8 | 1.9 | 2.1 | 3 |
| *YMR204C* | *INP1* |  |  |  | 3 | 2.5 | 2.7 | 3 |
| *YOR054C* | *VHS3* |  |  |  | 3.1 | 2.9 | 3.1 | 3 |
| *YML119W* |  |  |  |  | 3.5 | 2.7 | 2.7 | 3 |
| *YMR119W-A* |  |  |  |  | 3.5 | 3.5 | 3.5 | 3 |
| *YLR203C* | *MSS51* |  |  |  | 3.6 | 3.2 | 3.4 | 3 |
| *YNL047C* | *SLM2* |  |  |  | 4.1 | 4.3 | 4.2 | 3 |
| *YOL118C* |  |  |  |  | 4.2 | 3.9 | 3.9 | 3 |
| *YCR007C* |  |  |  |  | 4.2 | 3.9 | 3.9 | 3 |
| *YEL028W* |  |  |  |  | 4.4 | 3.7 | 4 | 3 |
| *YDL010W* |  |  |  |  | 4.5 | 4.5 | 4.3 | 3 |
| *YBR138C* |  |  |  |  | 4.6 | 4.1 | 4.4 | 3 |
| *YDR291W* | *HRQ1* |  |  |  | 4.9 | 4.3 | 4.5 | 3 |
| *YML009C* | *MRPL39* |  |  |  | 5 | 3.8 | 4 | 3 |
| *YBL065W* |  |  |  |  | 5.9 | 5.2 | 5.4 | 3 |
| *YHL011C* | *PRS3* | 2.3 | 2.2 |  |  |  |  | 2 |
| *YNL010W* |  | 1.6 |  | 1.2 |  |  |  | 2 |
| *YLL033W* | *IRC19* | 1.5 |  | 1.6 |  |  |  | 2 |
| *YHR048W* |  | 1.5 |  | 1.7 |  |  |  | 2 |
| *YCR082W* | *AHC2* | 1.4 |  | 1.4 |  |  |  | 2 |
| *YOR183W* | *FYV12* | 1.7 |  | 1.6 |  |  |  | 2 |
| *YGR207C* |  | 1.1 | 1.1 |  |  |  |  | 2 |
| *YNL123W* | *NMA111* | 1.4 |  | 1.6 |  |  |  | 2 |
| *YPL029W* | *SUV3* | 2 | 2.3 |  |  |  |  | 2 |
| *YLR131C* | *ACE2* | 1.3 |  | 1.3 |  |  |  | 2 |
| *YDL222C* | *FMP45* | -1.9 | -1.8 |  |  |  |  | 2 |
| *YKL080W* | *VMA5* |  | -1.9 | -2.6 |  |  |  | 2 |
| *YMR133W* | *REC114* |  | 1.2 | 1.3 |  |  |  | 2 |
| *YML118W* | *NGL3* |  | 1.3 | 1.1 |  |  |  | 2 |
| *YGL252C* | *RTG2* |  | 1.4 |  | 2.6 |  |  | 2 |
| *YPL140C* | *MKK2* |  | 1.5 | 1.5 |  |  |  | 2 |
| *YDR034C* | *LYS14* |  | 1.5 | 1.4 |  |  |  | 2 |
| *YJR051W* | *OSM1* |  | 1.5 | 1.4 |  |  |  | 2 |
| *YIL035C* | *CKA1* |  | 1.7 | 1.6 |  |  |  | 2 |
| *YBL081W* |  |  | 1.8 | 1.6 |  |  |  | 2 |
| *YPR139C* | *VPS66* |  | 1.8 | 1.8 |  |  |  | 2 |
| *YBR156C* | *SLI15* |  | 2 | 1.6 |  |  |  | 2 |
| *YEL051W* | *VMA8* |  | -2.1 | -2.4 |  |  |  | 2 |
| *YML097C* | *VPS9* |  |  | -2 |  | -2.4 |  | 2 |
| *YGR209C* | *TRX2* |  |  | -1.1 |  |  | -1.6 | 2 |
| *YOR124C* | *UBP2* |  |  | 1.3 |  |  | 1.5 | 2 |
| *YGR092W* | *DBF2* |  |  | 1.4 |  |  | 2 | 2 |
| *YDL019C* | *OSH2* |  |  | 1.6 | 2.6 |  |  | 2 |
| *YGR205W* |  | 1.8 |  | 1.5 |  |  |  | 2 |
| *YKL066W* |  | 2.2 |  | 2.1 |  |  |  | 2 |
| *YPR157W* |  |  | 1.7 | 1.7 |  |  |  | 2 |
| *YBL051C* | *PIN4* |  | 1.7 |  | -4 |  |  | 2 |
| *YGR097W* | *ASK10* |  | 1.8 | 1.9 |  |  |  | 2 |
| *YHR034C* | *PIH1* |  | 2 | 1.8 |  |  |  | 2 |
| *YKL037W* |  |  |  | -2 |  | -2.1 |  | 2 |
| *YNL140C* |  |  |  | 1.7 | 4.2 |  |  | 2 |
| *YGR004W* | *PEX31* |  |  |  | -4.4 |  | -1.6 | 2 |
| *YJL122W* | *ALB1* |  |  |  | -3.1 | -1.2 |  | 2 |
| *YBR245C* | *ISW1* |  |  |  | -3 |  | -1.2 | 2 |
| *YDR051C* |  |  |  |  | -2.9 | -1.3 |  | 2 |
| *YDR253C* | *MET32* |  |  |  | 3.3 |  | 1.8 | 2 |
| *YNL148C* | *ALF1* |  |  |  |  | -3.15 | -3.9 | 2 |
| *YPL170W* | *DAP1* |  |  |  |  | -3.6 | -4.95 | 2 |
| *YDR457W* | *TOM1* |  |  |  | -2.7 | -2.45 |  | 2 |
| *YML028W* | *TSA1* |  |  |  |  | -2.7 | -4 | 2 |
| *YLR025W* | *SNF7* |  |  |  |  | -2.05 | -3.55 | 2 |
| *YAL026C* | *DRS2* |  |  |  |  | -2.2 | -3.4 | 2 |
| *YGL007W* | *BRP1* |  |  |  |  | -2.05 | -3.2 | 2 |
| *YJL204C* | *RCY1* |  |  |  |  | -2.1 | -4.6 | 2 |
| *YHR206W* | *SKN7* |  |  |  |  | -1.7 | -4.1 | 2 |
| *YML053C* |  |  |  |  |  | -1.3 | -1.3 | 2 |
| *YKL164C* | *PIR1* |  |  |  |  | -1.3 | -1.3 | 2 |
| *YOR012W* |  |  |  |  |  | -1.2 | -1.3 | 2 |
| *YJR019C* | *TES1* |  |  |  |  | -1.2 | -1.2 | 2 |
| *YBR286W* | *APE3* |  |  |  |  | 1.2 | 1.2 | 2 |
| *YCL058C* | *FYV5* |  |  |  |  | 1.9 | 2.15 | 2 |
| *YDR171W* | *HSP42* |  |  |  | -2 | -1.8 |  | 2 |
| *YCL050C* | *APA1* |  |  |  | -1.6 | -1.3 |  | 2 |
| *YDL239C* | *ADY3* |  |  |  | -1.6 | -1.3 |  | 2 |
| *YBR129C* | *OPY1* |  |  |  | 1.6 | 1.4 |  | 2 |
| *YGR038W* | *ORM1* |  |  |  | 2 | 1.5 |  | 2 |
| *YMR238W* | *DFG5* |  |  |  | 2.2 | 1.7 |  | 2 |
| *YDR336W* |  |  |  |  | 2.3 |  | 2.1 | 2 |
| *YCL005W* | *LDB16* |  |  |  | 2.6 | 2.1 |  | 2 |
| *YGR242W* |  |  |  |  | 3 | 2 |  | 2 |
| *YJL046W* |  |  |  |  | 4.7 |  | 3.1 | 2 |
| *YHR030C* | *SLT2* |  |  |  |  | -3.2 | -3.6 | 2 |
| *YHR045W* |  |  |  |  |  | -2.9 | -5.5 | 2 |
| *YDR136C* | *VPS61* |  |  |  |  | -1.9 | -2.8 | 2 |
| *YER188W* |  |  |  |  |  | -1.6 | -1.6 | 2 |
| *YLR380W* | *CSR1* |  |  |  |  | -1.5 | -1.7 | 2 |
| *YKL046C* | *DCW1* |  |  |  |  | -1.5 | -1.4 | 2 |
| *YER084W* |  |  |  |  |  | -1.4 | -1.7 | 2 |
| *YKL146W* | *AVT3* |  |  |  |  | -1.3 | -1.4 | 2 |
| *YPL179W* | *PPQ1* |  |  |  |  | -1.3 | -1.5 | 2 |
| *YMR153C-A* |  |  |  |  |  | -1.2 | -1.2 | 2 |
| *YNL105W* |  |  |  |  |  | 1.2 | 1.4 | 2 |
| *YDL032W* |  |  |  |  |  | 1.4 | 1.5 | 2 |
| *YLR036C* |  |  |  |  |  | 1.6 | 1.7 | 2 |
| *YGR049W* | *SCM4* |  |  |  |  | 1.7 | 1.7 | 2 |
| *YIL110W* | *MNI1* |  |  |  |  | 1.9 | 2.7 | 2 |
| *YDL082W* | *RPL13A* |  |  |  |  | 2.1 | 2.1 | 2 |
| *YDR385W* | *EFT2* |  |  |  |  | 2.3 | 2.8 | 2 |
| *YOR092W* | *ECM3* |  |  |  |  | 2.4 | 3 | 2 |
| *YHR120W* | *MSH1* | 1.5 |  |  |  |  |  | 1 |
| *YLR370C* | *ARC18* | 1.4 |  |  |  |  |  | 1 |
| *YJR105W* | *ADO1* | 1.3 |  |  |  |  |  | 1 |
| *YBL083C* |  | -1.2 |  |  |  |  |  | 1 |
| *YKL147C* |  | -1.2 |  |  |  |  |  | 1 |
| *YCL030C* | *HIS4* | 2.6 |  |  |  |  |  | 1 |
| *YDL202W* | *MRPL11* | 1.3 |  |  |  |  |  | 1 |
| *YLR248W* | *RCK2* |  | 1.2 |  |  |  |  | 1 |
| *YOR137C* | *SIA1* |  | 1.3 |  |  |  |  | 1 |
| *YFL021W* | *GAT1* |  |  | -4.1 |  |  |  | 1 |
| *YDR495C* | *VPS3* |  |  | -2.15 |  |  |  | 1 |
| *YIR033W* | *MGA2* |  |  | -1.9 |  |  |  | 1 |
| *YKL077W* |  |  |  | -1.5 |  |  |  | 1 |
| *YMR021C* | *MAC1* |  |  | -1.4 |  |  |  | 1 |
| *YMR123W* | *PKR1* |  |  | -1.4 |  |  |  | 1 |
| *YOR068C* | *VAM10* |  |  | -1.3 |  |  |  | 1 |
| *YJR053W* | *BFA1* |  |  | 1.1 |  |  |  | 1 |
| *YMR152W* | *YIM1* |  |  | 1.1 |  |  |  | 1 |
| *YJL092W* | *HPR5* |  |  | 1.1 |  |  |  | 1 |
| *YLR187W* | *SKG3* |  |  | 1.3 |  |  |  | 1 |
| *YLR044C* | *PDC1* |  |  | 1.3 |  |  |  | 1 |
| *YDR447C* | *RPS17B* |  |  | 1.3 |  |  |  | 1 |
| *YOR078W* | *BUD21* |  |  | 1.3 |  |  |  | 1 |
| *YDL158C* |  |  |  | 1.4 |  |  |  | 1 |
| *YBR215W* | *HPC2* |  |  | 1.4 |  |  |  | 1 |
| *YDL191W* | *RPL35A* |  |  | 1.4 |  |  |  | 1 |
| *YJR079W* |  |  |  | 1.5 |  |  |  | 1 |
| *YGR231C* | *PHB2* |  |  | 1.6 |  |  |  | 1 |
| *YDR452W* | *PPN1* |  |  | 1.6 |  |  |  | 1 |
| *YBR159W* | *IFA38* |  |  | 1.6 |  |  |  | 1 |
| *YGR282C* | *BGL2* |  |  | 1.7 |  |  |  | 1 |
| *YJR021C* | *REC107* | -1.5 |  |  |  |  |  | 1 |
| *YJL095W* | *BCK1* |  | -2.8 |  |  |  |  | 1 |
| *YMR063W* | *RIM9* |  | 1.5 |  |  |  |  | 1 |
| *YPR044C* | *OPI11* |  | 2.4 |  |  |  |  | 1 |
| *YOR036W* | *PEP12* |  |  | -2.9 |  |  |  | 1 |
| *YGL095C* | *VPS45* |  |  | -2.6 |  |  |  | 1 |
| *YDR323C* | *PEP7* |  |  | -2.2 |  |  |  | 1 |
| *YEL027W* | *CUP5* |  |  | -1.9 |  |  |  | 1 |
| *YLR174W* | *IDP2* |  |  | 1.3 |  |  |  | 1 |
| *YMR289W* | *ABZ2* |  |  | 1.9 |  |  |  | 1 |
| *YGL200C* | *EMP24* |  |  | 2.2 |  |  |  | 1 |
| *YPL171C* | *OYE3* |  |  |  | -3.9 |  |  | 1 |
| *YNL292W* | *PUS4* |  |  |  | -3.9 |  |  | 1 |
| *YJR091C* | *JSN1* |  |  |  | -3.7 |  |  | 1 |
| *YLR152C* |  |  |  |  | -3.6 |  |  | 1 |
| *YHR110W* | *ERP5* |  |  |  | -3.5 |  |  | 1 |
| *YDR107C* |  |  |  |  | -3.5 |  |  | 1 |
| *YPL156C* | *PRM4* |  |  |  | -3.4 |  |  | 1 |
| *YJL123C* |  |  |  |  | -3.4 |  |  | 1 |
| *YER020W* | *GPA2* |  |  |  | -3.3 |  |  | 1 |
| *YNL314W* | *DAL82* |  |  |  | -3.3 |  |  | 1 |
| *YHR049C-A* |  |  |  |  | -3.3 |  |  | 1 |
| *YLL024C* | *SSA2* |  |  |  | -3.2 |  |  | 1 |
| *YFR039C* |  |  |  |  | -3.2 |  |  | 1 |
| *YBL089W* | *AVT5* |  |  |  | -3.2 |  |  | 1 |
| *YPR054W* | *SMK1* |  |  |  | -3.2 |  |  | 1 |
| *YLR381W* | *CTF3* |  |  |  | -3.2 |  |  | 1 |
| *YOR233W* | *KIN4* |  |  |  | -3.1 |  |  | 1 |
| *YDR068W* | *DOS2* |  |  |  | -3.1 |  |  | 1 |
| *YMR023C* | *MSS1* |  |  |  | -3 |  |  | 1 |
| *YER049W* | *TPA1* |  |  |  | -3 |  |  | 1 |
| *YER075C* | *PTP3* |  |  |  | -3 |  |  | 1 |
| *YOR153W* | *PDR5* |  |  |  | -3 |  |  | 1 |
| *YHR179W* | *OYE2* |  |  |  | -3 |  |  | 1 |
| *YOR215C* |  |  |  |  | -3 |  |  | 1 |
| *YOL104C* | *NDJ1* |  |  |  | -3 |  |  | 1 |
| *YGR258C* | *RAD2* |  |  |  | -3 |  |  | 1 |
| *YBR158W* | *AMN1* |  |  |  | -2.9 |  |  | 1 |
| *YBR250W* | *SPO23* |  |  |  | -2.9 |  |  | 1 |
| *YKL008C* | *LAC1* |  |  |  | -2.9 |  |  | 1 |
| *YDR490C* | *PKH1* |  |  |  | -2.9 |  |  | 1 |
| *YPR090W* |  |  |  |  | -2.9 |  |  | 1 |
| *YJL078C* | *PRY3* |  |  |  | -2.8 |  |  | 1 |
| *YPR092W* |  |  |  |  | -2.8 |  |  | 1 |
| *YPR014C* |  |  |  |  | -2.8 |  |  | 1 |
| *YKL128C* | *PMU1* |  |  |  | -2.8 |  |  | 1 |
| *YHR006W* | *STP2* |  |  |  | -2.8 |  |  | 1 |
| *YAL046C* |  |  |  |  | -2.8 |  |  | 1 |
| *YBR225W* |  |  |  |  | -2.8 |  |  | 1 |
| *YML066C* | *SMA2* |  |  |  | -2.7 |  |  | 1 |
| *YDR097C* | *MSH6* |  |  |  | -2.7 |  |  | 1 |
| *YKL148C* | *SDH1* |  |  |  | -2.7 |  |  | 1 |
| *YJL145W* | *SFH5* |  |  |  | -2.7 |  |  | 1 |
| *YGR126W* |  |  |  |  | -2.6 |  |  | 1 |
| *YER156C* |  |  |  |  | -2.6 |  |  | 1 |
| *YKR047W* |  |  |  |  | -2.6 |  |  | 1 |
| *YGL232W* | *TAN1* |  |  |  | -2.6 |  |  | 1 |
| *YJR148W* | *BAT2* |  |  |  | -2.6 |  |  | 1 |
| *YDL027C* |  |  |  |  | -2.6 |  |  | 1 |
| *YFR041C* | *ERJ5* |  |  |  | -2.6 |  |  | 1 |
| *YGR275W* | *RTT102* |  |  |  | -2.5 |  |  | 1 |
| *YNL013C* |  |  |  |  | -2.5 |  |  | 1 |
| *YPL033C* |  |  |  |  | -2.5 |  |  | 1 |
| *YHR096C* | *HXT5* |  |  |  | -2.5 |  |  | 1 |
| *YMR135W-A* |  |  |  |  | -2.5 |  |  | 1 |
| *YPR188C* | *MLC2* |  |  |  | -2.5 |  |  | 1 |
| *YDR063W* |  |  |  |  | -2.5 |  |  | 1 |
| *YBR177C* | *EHT1* |  |  |  | -2.5 |  |  | 1 |
| *YJL126W* | *NIT2* |  |  |  | -2.5 |  |  | 1 |
| *YPR083W* | *MDM36* |  |  |  | -2.4 |  |  | 1 |
| *YPR192W* | *AQY1* |  |  |  | -2.4 |  |  | 1 |
| *YGR271W* | *SLH1* |  |  |  | -2.4 |  |  | 1 |
| *YJL043W* |  |  |  |  | -2.4 |  |  | 1 |
| *YLR077W* | *FMP25* |  |  |  | -2.4 |  |  | 1 |
| *YML052W* | *SUR7* |  |  |  | -2.4 |  |  | 1 |
| *YBL064C* | *PRX1* |  |  |  | -2.4 |  |  | 1 |
| *YDR421W* | *ARO80* |  |  |  | -2.4 |  |  | 1 |
| *YHR138C* |  |  |  |  | -2.3 |  |  | 1 |
| *YNL122C* |  |  |  |  | -2.3 |  |  | 1 |
| *YJL178C* | *ATG27* |  |  |  | -2.3 |  |  | 1 |
| *YCR019W* | *MAK32* |  |  |  | -2.3 |  |  | 1 |
| *YPL105C* |  |  |  |  | -2.3 |  |  | 1 |
| *YIL055C* |  |  |  |  | -2.3 |  |  | 1 |
| *YEL040W* | *UTR2* |  |  |  | -2.3 |  |  | 1 |
| *YIR003W* |  |  |  |  | -2.3 |  |  | 1 |
| *YKL100C* |  |  |  |  | -2.3 |  |  | 1 |
| *YOL085C* |  |  |  |  | -2.3 |  |  | 1 |
| *YOR172W* | *YRM1* |  |  |  | -2.2 |  |  | 1 |
| *YLR209C* | *PNP1* |  |  |  | -2.2 |  |  | 1 |
| *YPL149W* | *ATG5* |  |  |  | -2.2 |  |  | 1 |
| *YMR234W* | *RNH1* |  |  |  | -2.2 |  |  | 1 |
| *YML120C* | *NDI1* |  |  |  | -2.1 |  |  | 1 |
| *YLR112W* |  |  |  |  | -2.1 |  |  | 1 |
| *YKL096W* | *CWP1* |  |  |  | -2.1 |  |  | 1 |
| *YNL311C* | *SKP2* |  |  |  | -2 |  |  | 1 |
| *YOR325W* |  |  |  |  | -1.9 |  |  | 1 |
| *YMR180C* | *CTL1* |  |  |  | -1.8 |  |  | 1 |
| *YLL038C* | *ENT4* |  |  |  | -1.8 |  |  | 1 |
| *YPR079W* | *MRL1* |  |  |  | -1.7 |  |  | 1 |
| *YER045C* | *ACA1* |  |  |  | -1.7 |  |  | 1 |
| *YMR276W* | *DSK2* |  |  |  | -1.7 |  |  | 1 |
| *YIL173W* | *VTH1* |  |  |  | -1.7 |  |  | 1 |
| *YKL097C* |  |  |  |  | -1.6 |  |  | 1 |
| *YOR017W* | *PET127* |  |  |  | -1.6 |  |  | 1 |
| *YDL241W* |  |  |  |  | -1.6 |  |  | 1 |
| *YPL245W* |  |  |  |  | -1.6 |  |  | 1 |
| *YGL096W* | *TOS8* |  |  |  | 1.4 |  |  | 1 |
| *YER180C* | *ISC10* |  |  |  | 1.5 |  |  | 1 |
| *YOL131W* |  |  |  |  | 1.7 |  |  | 1 |
| *YNL094W* | *APP1* |  |  |  | 1.9 |  |  | 1 |
| *YMR065W* | *KAR5* |  |  |  | 1.9 |  |  | 1 |
| *YLL052C* | *AQY2* |  |  |  | 2 |  |  | 1 |
| *YPL108W* |  |  |  |  | 2 |  |  | 1 |
| *YER032W* | *FIR1* |  |  |  | 2 |  |  | 1 |
| *YLR432W* | *IMD3* |  |  |  | 2 |  |  | 1 |
| *YGR066C* |  |  |  |  | 2 |  |  | 1 |
| *YER150W* | *SPI1* |  |  |  | 2.1 |  |  | 1 |
| *YBL003C* | *HTA2* |  |  |  | 2.2 |  |  | 1 |
| *YPL096W* | *PNG1* |  |  |  | 2.3 |  |  | 1 |
| *YIR027C* | *DAL1* |  |  |  | 2.3 |  |  | 1 |
| *YNL325C* | *FIG4* |  |  |  | 2.4 |  |  | 1 |
| *YNL125C* | *ESBP6* |  |  |  | 2.5 |  |  | 1 |
| *YDL133W* |  |  |  |  | 2.5 |  |  | 1 |
| *YLR074C* | *BUD20* |  |  |  | 2.5 |  |  | 1 |
| *YAR043C* |  |  |  |  | 2.5 |  |  | 1 |
| *YMR177W* | *MMT1* |  |  |  | 2.5 |  |  | 1 |
| *YIL090W* | *ICE2* |  |  |  | 2.7 |  |  | 1 |
| *YNL070W* | *TOM7* |  |  |  | 2.8 |  |  | 1 |
| *YGL258W* | *VEL1* |  |  |  | 2.9 |  |  | 1 |
| *YBR051W* |  |  |  |  | 3.4 |  |  | 1 |
| *YNL212W* | *VID27* |  |  |  | 3.5 |  |  | 1 |
| *YDR524C* | *AGE1* |  |  |  | 3.7 |  |  | 1 |
| *YDR261C* | *EXG2* |  |  |  | 3.7 |  |  | 1 |
| *YLL055W* |  |  |  |  | 3.9 |  |  | 1 |
| *YHR010W* | *RPL27A* |  |  |  |  | -2.7 |  | 1 |
| *YBR035C* | *PDX3* |  |  |  |  | -1.7 |  | 1 |
| *YKL199C* |  |  |  |  |  | -1.5 |  | 1 |
| *YGL010W* |  |  |  |  |  | -1.5 |  | 1 |
| *YGL164C* | *YRB30* |  |  |  |  | -1.5 |  | 1 |
| *YGL160W* |  |  |  |  |  | -1.2 |  | 1 |
| *YOR120W* | *GCY1* |  |  |  |  | 1.4 |  | 1 |
| *YDR354W* | *TRP4* |  |  |  |  | 1.7 |  | 1 |
| *YDL226C* | *GCS1* |  |  |  |  |  | -4 | 1 |
| *YDR098C* | *GRX3* |  |  |  |  |  | -3.35 | 1 |
| *YPR173C* | *VPS4* |  |  |  |  |  | -3.1 | 1 |
| *YOR322C* | *LDB19* |  |  |  |  |  | -2.85 | 1 |
| *YMR275C* | *BUL1* |  |  |  |  |  | -2.65 | 1 |
| *YLR417W* | *VPS36* |  |  |  |  |  | -3 | 1 |
| *YPR074C* | *TKL1* |  |  |  |  |  | -2.7 | 1 |
| *YIL098C* | *FMC1* |  |  |  |  |  | -2.3 | 1 |
| *YJR102C* | *VPS25* |  |  |  |  |  | -2.3 | 1 |
| *YCL008C* | *STP22* |  |  |  |  |  | -2.2 | 1 |
| *YJR033C* | *RAV1* |  |  |  |  |  | -2.1 | 1 |
| *YHL007C* | *STE20* |  |  |  |  |  | -2.1 | 1 |
| *YOR297C* | *TIM18* |  |  |  |  |  | -2.15 | 1 |
| *YMR154C* | *RIM13* |  |  |  |  |  | -2 | 1 |
| *YKR019C* | *IRS4* |  |  |  |  |  | -1.75 | 1 |
| *YBR151W* | *APD1* |  |  |  |  |  | -1.9 | 1 |
| *YJL154C* | *VPS35* |  |  |  |  |  | -2.1 | 1 |
| *YIL067C* |  |  |  |  |  |  | -1.8 | 1 |
| *YNL294C* | *RIM21* |  |  |  |  |  | -1.9 | 1 |
| *YLR047C* | *FRE8* |  |  |  |  |  | -1.85 | 1 |
| *YPL120W* | *VPS30* |  |  |  |  |  | -1.6 | 1 |
| *YBR290W* | *BSD2* |  |  |  |  |  | -1.5 | 1 |
| *YKL215C* |  |  |  |  |  |  | -1.4 | 1 |
| *YJL004C* | *SYS1* |  |  |  |  |  | -1.3 | 1 |
| *YLR369W* | *SSQ1* |  |  |  |  |  | 1.95 | 1 |
| *YML010C-B* |  |  |  |  |  |  | 1.8 | 1 |
| *YNL119W* | *NCS2* |  |  |  |  |  | 2.4 | 1 |
| *YKL177W* |  |  |  |  | -2.2 |  |  | 1 |
| *YKL161C* |  |  |  |  | -1.6 |  |  | 1 |
| *YNL338W* |  |  |  |  | -1.5 |  |  | 1 |
| *YGR028W* | *MSP1* |  |  |  | 1.9 |  |  | 1 |
| *YGL227W* | *VID30* |  |  |  | 1.9 |  |  | 1 |
| *YHR079C-B* |  |  |  |  | 2 |  |  | 1 |
| *YBR173C* | *UMP1* |  |  |  | 2 |  |  | 1 |
| *YLR341W* | *SPO77* |  |  |  | 2.1 |  |  | 1 |
| *YBR016W* |  |  |  |  | 2.1 |  |  | 1 |
| *YLR188W* | *MDL1* |  |  |  | 2.3 |  |  | 1 |
| *YCR101C* |  |  |  |  | 2.4 |  |  | 1 |
| *YKR041W* |  |  |  |  | 3.6 |  |  | 1 |
| *YHL025W* | *SNF6* |  |  |  |  | -3.5 |  | 1 |
| *YCR094W* | *CDC50* |  |  |  |  | -2.6 |  | 1 |
| *YOR141C* | *ARP8* |  |  |  |  | -2.6 |  | 1 |
| *YKR048C* | *NAP1* |  |  |  |  | -2.5 |  | 1 |
| *YCR028C* | *FEN2* |  |  |  |  | -2.4 |  | 1 |
| *YPL060W* | *LPE10* |  |  |  |  | -2.2 |  | 1 |
| *YMR278W* |  |  |  |  |  | -1.4 |  | 1 |
| *YKL184W* | *SPE1* |  |  |  |  | -1.2 |  | 1 |
| *YML027W* | *YOX1* |  |  |  |  | 1.2 |  | 1 |
| *YPR122W* | *AXL1* |  |  |  |  | 1.3 |  | 1 |
| *YBR027C* |  |  |  |  |  | 1.3 |  | 1 |
| *YML100W-A* |  |  |  |  |  | 1.4 |  | 1 |
| *YNL108C* |  |  |  |  |  | 1.6 |  | 1 |
| *YFL041W* | *FET5* |  |  |  |  | 1.9 |  | 1 |
| *YDR384C* | *ATO3* |  |  |  |  | 2.2 |  | 1 |
| *YPL065W* | *VPS28* |  |  |  |  |  | -2.9 | 1 |
| *YJL053W* | *PEP8* |  |  |  |  |  | -2.5 | 1 |
| *YLR262C* | *YPT6* |  |  |  |  |  | -2.4 | 1 |
| *YGL212W* | *VAM7* |  |  |  |  |  | -2.2 | 1 |
| *YNL231C* | *PDR16* |  |  |  |  |  | -2.2 | 1 |
| *YOR132W* | *VPS17* |  |  |  |  |  | -1.8 | 1 |
| *YCR049C* |  |  |  |  |  |  | -1.7 | 1 |
| *YDR455C* |  |  |  |  |  |  | -1.7 | 1 |
| *YOR164C* |  |  |  |  |  |  | -1.7 | 1 |
| *YBR131W* | *CCZ1* |  |  |  |  |  | -1.6 | 1 |
| *YDR497C* | *ITR1* |  |  |  |  |  | -1.5 | 1 |
| *YKR014C* | *YPT52* |  |  |  |  |  | -1.5 | 1 |
| *YJL027C* |  |  |  |  |  |  | -1.5 | 1 |
| *YER118C* | *SHO1* |  |  |  |  |  | -1.3 | 1 |
| *YCL026C-A* | *FRM2* |  |  |  |  |  | -1.1 | 1 |
| *YPL265W* | *DIP5* |  |  |  |  |  | 1.2 | 1 |
| *YOR043W* | *WHI2* |  |  |  |  |  | 1.3 | 1 |
| *YIL112W* | *HOS4* |  |  |  |  |  | 1.5 | 1 |
| *YBL079W* | *NUP170* |  |  |  |  |  | 1.6 | 1 |
| *YNL109W* |  |  |  |  |  |  | 1.7 | 1 |
| *YMR188C* | *MRPS17* |  |  |  |  |  | 1.7 | 1 |
| *YBR075W* |  |  |  |  |  |  | 2.1 | 1 |
| *YDR237W* | *MRPL7* |  |  |  |  |  | 2.2 | 1 |
| *YDR388W* | *RVS167* |  |  |  |  |  | 2.4 | 1 |
