# Supplementary material for: Genome-Wide Functional Profiling Reveals Genes Required for Tolerance to Benzene Metabolites in Yeast
Source: PLoS One. 2011 Aug 30;6(8):e24205. doi: 10.1371/journal.pone.0024205 (PMC3166172; doi:10.1371/journal.pone.0024205)
Supplement: Table S3 — Complete list of yeast genes (n = 604) identified by DSSA after treatment with 1,2,4-benzenetriol (BT), ranked by the number of hits in 6 treatments. Yeast pools were exposed to 3 different concentrations of 1,2,4-benzenetriol for two generation-points, for a total of 6 treatments. The yeast ORFs/genes correspond to deletion strains that exhibited a significant change in growth in at least one treatment with 1,2,4-benzenetriol (q<0.05). Numeric values are fitness scores (log2 ratios) calculated only for significant genes in each individual treatment. Empty cells indicate that the gene was not significant in that particular treatment. (DOC) [file pone.0024205.s009.doc]

**Table S3. Complete list of yeast genes (n = 604) identified by DSSA after treatment with 1,2,4-benzenetriol (BT), ranked by the number of hits in 6 treatments.** Yeast pools were exposed to 3 different concentrations of 1,2,4-benzenetriol for two generation-points, for a total of 6 treatments. The yeast ORFs / genes correspond to deletion strains that exhibited a significant change in growth in at least one treatment with 1,2,4-benzenetriol (q < 0.05). Numeric values are fitness scores (log_2_ ratios) calculated only for significant genes in each individual treatment. Empty cells indicate that the gene was not significant in that particular treatment.

|  |  | 5 generations | | | 15 generations | | |  |
| --- | --- | --- | --- | --- | --- | --- | --- | --- |
| ORF | Gene | 25% IC_20_ | 50% IC_20_ | IC_20_ | 25% IC_20_ | 50% IC_20_ | IC_20_ | # of hits |
|  |  | 87.5µM | 175µM | 350µM | 87.5µM | 175µM | 350µM |  |
| *YPL157W* | *TGS1* | 2.85 | 3.6 | 3.6 | 2.3 | 3.7 | 4 | 6 |
| *YLR169W* |  | 2.1 | 2 | 2.4 | 3.3 | 2.6 | 2.2 | 6 |
| *YBR213W* | *MET8* | 2.7 | 2.7 | 2.8 | 2.7 | 2.7 | 2.9 | 6 |
| *YDR458C* | *HEH2* | 2.9 | 2.65 | 2.75 | 2.9 | 3.5 | 3.7 | 6 |
| *YOR133W* | *EFT1* | 2.8 | 3 | 2.9 | 2.2 | 3.4 | 3.4 | 6 |
| *YMR304W* | *UBP15* | 2.7 | 3.7 | 1.8 |  | 2.6 | 3.5 | 5 |
| *YJR088C* | *NA* |  | 2.7 | 2.8 | 2.35 | 3.2 | 3.3 | 5 |
| *YDL223C* | *HBT1* | 2.2 | 2.1 | 2.6 |  | 2.8 | 2.7 | 5 |
| *YEL012W* | *UBC8* | 3.1 | 2.8 | 3 | 2.6 | 3 |  | 5 |
| *YDR153C* | *ENT5* | 3.2 | 3 |  | 2.3 | 3.4 | 3.5 | 5 |
| *YGR263C* | *SAY1* |  | 1.9 | 2.2 | 2.2 | 2.8 | 3.4 | 5 |
| *YFR018C* |  |  | 2.2 | 2.7 | 2.5 | 2.9 | 3.1 | 5 |
| *YBR292C* |  | -2.3 | -1.5 | -1.2 |  |  | -1.5 | 4 |
| *YJR044C* | *VPS55* |  | 2.1 | 1.9 |  | 2.5 | 2.5 | 4 |
| *YOR314W* |  |  | 2.1 | 2 |  | 2.3 | 2.3 | 4 |
| *YOR352W* |  |  | 2.1 |  | 2.2 | -1.7 | -1.8 | 4 |
| *YJL120W* |  |  |  | -3.25 | -2.95 | -4.5 | -4.6 | 4 |
| *YDR414C* | *ERD1* |  |  | 1.3 | 2 | 2.2 | 4.35 | 4 |
| *YBR114W* | *RAD16* |  |  | 2.2 | 3.2 | 2.8 | 2.7 | 4 |
| *YKR078W* |  |  |  | 2.2 | 2.4 | 2.2 | 2.2 | 4 |
| *YGR130C* |  | -2.1 | -2.4 | -2.2 |  |  |  | 3 |
| *YLR131C* | *ACE2* | 2.1 | 1.6 | 1.5 |  |  |  | 3 |
| *YER066C-A* |  | 2.2 | 2.1 | 2.3 |  |  |  | 3 |
| *YGL087C* | *MMS2* | 2.3 | 2.2 |  | 2.8 |  |  | 3 |
| *YBR187W* | *GDT1* | 2.3 | 2.8 | 3.2 |  |  |  | 3 |
| *YDR105C* | *TMS1* | 2.5 | 2.4 | 2.1 |  |  |  | 3 |
| *YER002W* | *NOP16* | 2.8 | 3 | 3.1 |  |  |  | 3 |
| *YDR179C* | *CSN9* | 3 |  |  |  | -2 | -2.2 | 3 |
| *YGL138C* |  | 3 |  | 2.2 |  |  | 2.8 | 3 |
| *YOR021C* |  | 3 | 2.8 | 2 |  |  |  | 3 |
| *YMR272C* | *SCS7* | 2.7 | 1.8 |  |  |  | 1.8 | 3 |
| *YBR044C* | *TCM62* |  | 2.3 |  |  | 2.3 | 2.5 | 3 |
| *YJL121C* | *RPE1* |  |  | -2.6 | -3.1 |  | -4.2 | 3 |
| *YHR206W* | *SKN7* |  |  | -2.45 | -3.7 | -4.7 |  | 3 |
| *YML028W* | *TSA1* |  |  | -2.2 |  | -2.7 | -3.3 | 3 |
| *YGL148W* | *ARO2* |  |  | -1.3 |  | -2.6 | -3.05 | 3 |
| *YPR074C* | *TKL1* |  |  | -1.3 |  | -3.75 | -3.9 | 3 |
| *YLR380W* | *CSR1* |  |  | -1.1 |  | -4.6 | -2.8 | 3 |
| *YDR175C* | *RSM24* |  |  | 1.5 |  | 3.3 | 4.1 | 3 |
| *YLR133W* | *CKI1* | 2.4 | 2.2 | 2.3 |  |  |  | 3 |
| *YMR294W-A* |  |  | 2.1 |  | 2.3 | 2.2 |  | 3 |
| *YNL101W* | *AVT4* |  | 2.1 |  |  | 2.8 | 2.8 | 3 |
| *YBR035C* | *PDX3* |  |  |  | -2.2 | -2.4 | -2.6 | 3 |
| *YIL162W* | *SUC2* |  |  |  | -2 | -3.3 | -3.7 | 3 |
| *YDR314C* | *RAD34* |  |  |  | 1.8 | 2.3 | 2.1 | 3 |
| *YPL015C* | *HST2* |  |  |  | 1.9 | 2 | 2.1 | 3 |
| *YNL278W* | *CAF120* |  |  |  | 2 | 2.4 | 2.3 | 3 |
| *YDR321W* | *ASP1* |  |  |  | 2 | 1.9 | 2 | 3 |
| *YJR110W* | *YMR1* |  |  |  | 2 | 2.3 | 2.4 | 3 |
| *YEL068C* |  |  |  |  | 2 | 2.7 | 3 | 3 |
| *YJR080C* | *FMP26* |  |  |  | 2 | 2.6 | 2.6 | 3 |
| *YKR088C* | *TVP38* |  |  |  | 2.1 | 2 | 2 | 3 |
| *YPL052W* | *OAZ1* |  |  |  | 2.1 | 2.3 | 2.4 | 3 |
| *YMR289W* | *ABZ2* |  |  |  | 2.2 | 3.5 | 3.7 | 3 |
| *YCR022C* |  |  |  |  | 2.2 | 2.5 | 2.5 | 3 |
| *YHR177W* |  |  |  |  | 2.2 | 2.5 | 2.6 | 3 |
| *YDL161W* | *ENT1* |  |  |  | 2.2 | 2.4 | 2.6 | 3 |
| *YBL107C* |  |  |  |  | 2.3 | 2.2 | 2.1 | 3 |
| *YOR137C* | *SIA1* |  |  |  | 2.3 | 2.3 | 2.1 | 3 |
| *YOL101C* | *IZH4* |  |  |  | 2.4 | 2 | 2.2 | 3 |
| *YGL229C* | *SAP4* |  |  |  | 2.4 | 2.3 | 2.2 | 3 |
| *YJL108C* | *PRM10* |  |  |  | 2.4 | 2.7 | 2.7 | 3 |
| *YLR042C* |  |  |  |  | 2.5 | 2.8 | 3.2 | 3 |
| *YNL010W* |  |  |  |  | 2.5 | 2.5 | 2.4 | 3 |
| *YFL011W* | *HXT10* |  |  |  | 2.5 | 2.7 | 3 | 3 |
| *YBR169C* | *SSE2* |  |  |  | 2.5 | 2.3 | 1.8 | 3 |
| *YDR326C* | *YSP2* |  |  |  | 2.6 | 2.2 | 2 | 3 |
| *YBR273C* | *UBX7* |  |  |  | 2.6 | 2.1 | 2 | 3 |
| *YGR107W* |  |  |  |  | 2.7 | 2.6 | 2.5 | 3 |
| *YFL019C* |  |  |  |  | 2.7 | 3.6 | 4.4 | 3 |
| *YER087C-A* |  |  |  |  | 2.7 | 2.9 | 2.7 | 3 |
| *YER084W* |  |  |  |  | 2.7 | -2.3 | -2.7 | 3 |
| *YGL232W* | *TAN1* |  |  |  | 2.8 | 2.6 | 2.7 | 3 |
| *YBR019C* | *GAL10* |  |  |  | 2.9 | 2.3 | 2.3 | 3 |
| *YPR200C* | *ARR2* |  |  |  | 3 | 2.4 | 1.8 | 3 |
| *YNR024W* |  |  |  |  | 3 | 2.9 | 2.6 | 3 |
| *YGR230W* | *BNS1* |  |  |  | 3 | 3.3 | 3.4 | 3 |
| *YOR173W* | *DCS2* |  |  |  | 3 | 3.6 | 3.6 | 3 |
| *YMR291W* |  |  |  |  | 3 | 3.1 | 2.9 | 3 |
| *YNL122C* |  |  |  |  | 3.1 | 2.5 | 2.1 | 3 |
| *YIL032C* |  |  |  |  | 3.1 | 3.3 | 3.5 | 3 |
| *YDL230W* | *PTP1* |  |  |  | 3.1 | 3.3 | 3.4 | 3 |
| *YPR011C* |  |  |  |  | 3.2 | 3.7 | 3.9 | 3 |
| *YDR319C* |  |  |  |  | 3.2 | 2.2 | 1.8 | 3 |
| *YFL044C* | *OTU1* |  |  |  | 3.3 | 2.5 | 2.4 | 3 |
| *YPR128C* | *ANT1* |  |  |  | 3.3 | 3.5 | 3.4 | 3 |
| *YFL013W-A* |  |  |  |  | 3.3 | 3.7 | 4.2 | 3 |
| *YDR312W* | *SSF2* |  |  |  | 3.4 | 3.7 | 3.9 | 3 |
| *YDL135C* | *RDI1* |  |  |  | 2.85 | 2.9 | 2.7 | 3 |
| *YBR113W* |  |  |  |  | 4.2 | 4.4 | 4.4 | 3 |
| *YFL032W* |  |  |  |  | 4.4 | 4.4 | 4.6 | 3 |
| *YMR042W* | *ARG80* |  |  |  | 4.4 | 3.6 | 3.2 | 3 |
| *YFR036W* | *CDC26* |  |  |  | -3.1 | -4.3 | -4.4 | 3 |
| *YDR220C* |  |  |  |  | 2 | 2.2 | 2 | 3 |
| *YNL109W* |  |  |  |  | 2 | 1.7 | 1.9 | 3 |
| *YBR016W* |  |  |  |  | 2 | 3.1 | 3.1 | 3 |
| *YMR280C* | *CAT8* |  |  |  | 2.1 | 2.3 | 2.3 | 3 |
| *YBR170C* | *NPL4* |  |  |  | 2.2 | 3.1 | 3.2 | 3 |
| *YBR149W* | *ARA1* |  |  |  | 2.4 | 2.3 | 2.4 | 3 |
| *YLR203C* | *MSS51* |  |  |  | 2.4 | 3.4 | 3.5 | 3 |
| *YGL227W* | *VID30* |  |  |  | 2.4 | 2.2 | 2 | 3 |
| *YHR153C* | *SPO16* |  |  |  | 2.4 | 2.3 | 2.3 | 3 |
| *YKL133C* |  |  |  |  | 2.4 | 3.2 | 2.8 | 3 |
| *YGR038W* | *ORM1* |  |  |  | 2.5 | 1.9 | 2.2 | 3 |
| *YFR026C* |  |  |  |  | 2.6 | 2.1 | 2.1 | 3 |
| *YDR336W* |  |  |  |  | 2.6 | 3 | 3.1 | 3 |
| *YGR058W* |  |  |  |  | 2.7 | 2 | 2.4 | 3 |
| *YMR262W* |  |  |  |  | 2.8 | 2.4 | 2.5 | 3 |
| *YMR204C* | *INP1* |  |  |  | 2.9 | 3.1 | 3.1 | 3 |
| *YPR140W* | *TAZ1* |  |  |  | 3 | 2.8 | 2.8 | 3 |
| *YOL118C* |  |  |  |  | 3.1 | 4.3 | 4.2 | 3 |
| *YJR079W* |  |  |  |  | 3.1 | 4.3 | 4.4 | 3 |
| *YML119W* |  |  |  |  | 3.2 | 3.2 | 3.3 | 3 |
| *YDL010W* |  |  |  |  | 3.3 | 4.5 | 5 | 3 |
| *YEL028W* |  |  |  |  | 3.5 | 4.1 | 4.3 | 3 |
| *YKL096W-A* | *CWP2* |  |  |  | 3.5 | 2.7 | 2.5 | 3 |
| *YBR138C* |  |  |  |  | 3.6 | 4.8 | 4.9 | 3 |
| *YMR105C* | *PGM2* |  |  |  | 3.6 | 4.5 | 4.6 | 3 |
| *YDR291W* | *HRQ1* |  |  |  | 3.7 | 5 | 4.9 | 3 |
| *YOR092W* | *ECM3* |  |  |  | 3.8 | 4.8 | 4.9 | 3 |
| *YPR109W* |  |  |  |  | 4 | 4.5 | 4.8 | 3 |
| *YNL264C* | *PDR17* |  |  |  | 4.2 | 3.2 | 3.1 | 3 |
| *YKL174C* | *TPO5* |  |  |  | 4.2 | 5.9 | 5.4 | 3 |
| *YBL065W* |  |  |  |  | 4.2 | 6.2 | 6.4 | 3 |
| *YDL211C* |  | -1.7 |  | -1.3 |  |  |  | 2 |
| *YER156C* |  | -1.7 | -1.7 |  |  |  |  | 2 |
| *YOR330C* | *MIP1* | 2.1 |  | 1.9 |  |  |  | 2 |
| *YGR041W* | *BUD9* | 2.5 |  | 2.1 |  |  |  | 2 |
| *YER169W* | *RPH1* | 2.5 | 2.3 |  |  |  |  | 2 |
| *YGL203C* | *KEX1* | 2.5 |  | 3.4 |  |  |  | 2 |
| *YDL158C* |  | 3.1 |  | 2.4 |  |  |  | 2 |
| *YBR156C* | *SLI15* | 4 |  | 2 |  |  |  | 2 |
| *YKL080W* | *VMA5* |  | -2.1 | -1.7 |  |  |  | 2 |
| *YBR159W* | *IFA38* |  | 1.8 |  |  | 2.4 |  | 2 |
| *YMR286W* | *MRPL33* |  | 2.2 | 1.8 |  |  |  | 2 |
| *YIL139C* | *REV7* |  | 2.3 | 2.2 |  |  |  | 2 |
| *YGL226C-A* | *OST5* |  | 2.4 |  | 2.7 |  |  | 2 |
| *YMR250W* | *GAD1* |  | 2.4 | 2.2 |  |  |  | 2 |
| *YKL166C* | *TPK3* |  | 2.6 | 1.7 |  |  |  | 2 |
| *YCL030C* | *HIS4* |  | 2.8 | 2.6 |  |  |  | 2 |
| *YDL160C* | *DHH1* | -2.5 |  | -3 |  |  |  | 2 |
| *YGL071W* | *AFT1* |  | -2.9 | -1.65 |  |  |  | 2 |
| *YDR285W* | *ZIP1* |  |  | 2 | 4.3 |  |  | 2 |
| *YMR261C* | *TPS3* |  |  | 2.4 | 2.6 |  |  | 2 |
| *YCR027C* | *RHB1* | 1.9 |  |  | 2.8 |  |  | 2 |
| *YGR261C* | *APL6* | 2.7 | 3.5 |  |  |  |  | 2 |
| *YGL118C* |  | 2.8 |  | 2.7 |  |  |  | 2 |
| *YML070W* | *DAK1* | 2.9 | 3.7 |  |  |  |  | 2 |
| *YPL274W* | *SAM3* |  | 2.9 | 2.7 |  |  |  | 2 |
| *YIR028W* | *DAL4* |  | 3.7 | 3.6 |  |  |  | 2 |
| *YHR178W* | *STB5* |  |  | -2.3 |  |  | -3 | 2 |
| *YNL294C* | *RIM21* |  |  | -1.9 |  |  | -1.7 | 2 |
| *YLR047C* | *FRE8* |  |  | -1.8 |  |  | -2.2 | 2 |
| *YKL086W* | *SRX1* |  |  | -1.4 |  |  | -4.25 | 2 |
| *YBR175W* | *SWD3* |  |  | 2.7 | 2.4 |  |  | 2 |
| *YBR024W* | *SCO2* |  |  |  | 2.1 | 1.9 |  | 2 |
| *YCR011C* | *ADP1* |  |  |  | 2.3 | 2.4 |  | 2 |
| *YER092W* | *IES5* |  |  |  | 2.3 |  | 1.7 | 2 |
| *YNL123W* | *NMA111* |  |  |  | 2.4 | 2.2 |  | 2 |
| *YMR175W* | *SIP18* |  |  |  | 2.4 | 2.2 |  | 2 |
| *YOR041C* |  |  |  |  | 2.5 | 1.8 |  | 2 |
| *YGR192C* | *TDH3* |  |  |  | 2.5 | 2.3 |  | 2 |
| *YDR252W* | *BTT1* |  |  |  | 2.5 | 2.1 |  | 2 |
| *YKR096W* |  |  |  |  | 2.5 | 2.3 |  | 2 |
| *YDR496C* | *PUF6* |  |  |  | 2.5 | 2.2 |  | 2 |
| *YML115C* | *VAN1* |  |  |  | 2.6 |  | 2.3 | 2 |
| *YMR016C* | *SOK2* |  |  |  | 2.7 |  | 1.6 | 2 |
| *YIR030C* | *DCG1* |  |  |  | 2.7 | 2.1 |  | 2 |
| *YBR184W* |  |  |  |  | 2.8 | 2.1 |  | 2 |
| *YDR354W* | *TRP4* |  |  |  | 2.8 | 2.2 |  | 2 |
| *YFL021W* | *GAT1* |  |  |  | 3.1 | 3.7 |  | 2 |
| *YPL013C* | *MRPS16* |  |  |  | 2.75 | 2.8 |  | 2 |
| *YHR157W* | *REC104* |  |  |  | 3.8 | 4.2 |  | 2 |
| *YML079W* |  |  |  |  | 3.9 | 4.1 |  | 2 |
| *YIR037W* | *HYR1* |  |  |  |  | -4.3 | -4.6 | 2 |
| *YDL226C* | *GCS1* |  |  |  |  | -3.35 | -4.35 | 2 |
| *YLR025W* | *SNF7* |  |  |  |  | -2.5 | -3 | 2 |
| *YMR022W* | *QRI8* |  |  |  |  | -2.2 | -2.7 | 2 |
| *YDL018C* | *ERP3* |  |  |  |  | -2.1 | -2.3 | 2 |
| *YOL163W* |  |  |  |  |  | 1.7 | 1.5 | 2 |
| *YBR177C* | *EHT1* |  |  |  |  | 1.8 | 1.9 | 2 |
| *YBR225W* |  |  |  |  |  | 1.9 | 1.8 | 2 |
| *YDR430C* | *CYM1* |  |  |  |  | 1.9 | 2.2 | 2 |
| *YJL218W* |  |  |  |  |  | 2.3 | 2.6 | 2 |
| *YNR019W* | *ARE2* |  |  |  |  | 2.5 | 2.7 | 2 |
| *YDR377W* | *ATP17* |  |  |  |  | 2.7 | 3.6 | 2 |
| *YFL020C* | *PAU5* |  |  |  |  | 2.7 | 2.9 | 2 |
| *YBR216C* | *YBP1* |  |  |  |  | -4.5 | -4.4 | 2 |
| *YML022W* | *APT1* |  |  |  | 3.1 |  | 1.85 | 2 |
| *YBR103W* | *SIF2* |  |  |  | 2 |  | 2.55 | 2 |
| *YDR337W* | *MRPS28* |  |  |  |  | 2 | 2.85 | 2 |
| *YLR108C* |  |  |  |  |  | 2.3 | 2.8 | 2 |
| *YDL202W* | *MRPL11* |  |  |  | 2.1 |  | 2.85 | 2 |
| *YDR306C* |  |  |  |  | 2 | 1.9 |  | 2 |
| *YGL215W* | *CLG1* |  |  |  | 2 |  | 2.3 | 2 |
| *YDR391C* |  |  |  |  | 2.1 |  | 2.6 | 2 |
| *YKR041W* |  |  |  |  | 2.4 |  | 3.2 | 2 |
| *YGL242C* |  |  |  |  | 2.5 |  | 1.9 | 2 |
| *YGR242W* |  |  |  |  | 2.5 | 2.6 |  | 2 |
| *YMR135W-A* |  |  |  |  | 2.6 |  | 1.6 | 2 |
| *YOR054C* | *VHS3* |  |  |  | 2.7 | 3.3 |  | 2 |
| *YJL046W* |  |  |  |  | 2.7 |  | 3.8 | 2 |
| *YHR079C-B* |  |  |  |  | 2.8 | 2.3 |  | 2 |
| *YHR151C* |  |  |  |  | 3 | 2 |  | 2 |
| *YGR070W* | *ROM1* |  |  |  | 3 | 2.6 |  | 2 |
| *YGR157W* | *CHO2* |  |  |  | 5.2 | 2.4 |  | 2 |
| *YDR112W* | *IRC2* |  |  |  |  | -3.8 | -3.7 | 2 |
| *YEL056W* | *HAT2* |  |  |  |  | -3.3 | -3.4 | 2 |
| *YPL138C* | *SPP1* |  |  |  |  | -3.1 | -3.9 | 2 |
| *YMR153W* | *NUP53* |  |  |  |  | -2.6 | -2.6 | 2 |
| *YOR089C* | *VPS21* |  |  |  |  | -2.5 | -3.7 | 2 |
| *YGL094C* | *PAN2* |  |  |  |  | -2.3 | -2.4 | 2 |
| *YCR049C* |  |  |  |  |  | -2.3 | -3 | 2 |
| *YKL123W* |  |  |  |  |  | -2.3 | -2.4 | 2 |
| *YDR171W* | *HSP42* |  |  |  |  | -2.2 | -2.5 | 2 |
| *YHL026C* |  |  |  |  |  | -2 | -2.1 | 2 |
| *YKR033C* |  |  |  |  |  | -1.9 | -2 | 2 |
| *YDL236W* | *PHO13* |  |  |  |  | -1.9 | -1.9 | 2 |
| *YKL051W* | *SFK1* |  |  |  |  | -1.8 | -2 | 2 |
| *YCL050C* | *APA1* |  |  |  |  | -1.7 | -1.9 | 2 |
| *YOR338W* |  |  |  |  |  | -1.5 | -1.6 | 2 |
| *YKL071W* |  |  |  |  |  | -1.5 | -1.7 | 2 |
| *YNL105W* |  |  |  |  |  | 1.5 | 1.4 | 2 |
| *YGR028W* | *MSP1* |  |  |  |  | 1.9 | 2.2 | 2 |
| *YDR392W* | *SPT3* |  |  |  |  | 2 | 3.2 | 2 |
| *YLR286C* | *CTS1* |  |  |  |  | 2.1 | 2.3 | 2 |
| *YDR400W* | *URH1* |  |  |  |  | 2.1 | 2.1 | 2 |
| *YDL070W* | *BDF2* |  |  |  |  | 2.3 | 2.3 | 2 |
| *YMR299C* | *DYN3* |  |  |  |  | 2.3 | 2.3 | 2 |
| *YDR065W* |  |  |  |  |  | 2.4 | 2.9 | 2 |
| *YLR335W* | *NUP2* |  |  |  |  | 2.6 | 2.8 | 2 |
| *YDR385W* | *EFT2* |  |  |  |  | 2.6 | 3 | 2 |
| *YHR034C* | *PIH1* |  |  |  |  | 2.7 | 3.4 | 2 |
| *YMR119W-A* |  |  |  |  |  | 3.5 | 3.4 | 2 |
| *YNL047C* | *SLM2* |  |  |  |  | 4.1 | 4.2 | 2 |
| *YCR007C* |  |  |  |  |  | 4.2 | 4 | 2 |
| *YML009C* | *MRPL39* |  |  |  |  | 5.2 | 5.2 | 2 |
| *YLL043W* | *FPS1* | -4.5 |  |  |  |  |  | 1 |
| *YJL117W* | *PHO86* | -3.3 |  |  |  |  |  | 1 |
| *YLR396C* | *VPS33* | -2.4 |  |  |  |  |  | 1 |
| *YBR279W* | *PAF1* | -2.2 |  |  |  |  |  | 1 |
| *YKL097C* |  | -1.6 |  |  |  |  |  | 1 |
| *YGL147C* | *RPL9A* | -1.5 |  |  |  |  |  | 1 |
| *YKL090W* | *CUE2* | -1.4 |  |  |  |  |  | 1 |
| *YDL214C* | *PRR2* | 1.6 |  |  |  |  |  | 1 |
| *YOR019W* |  | 2.1 |  |  |  |  |  | 1 |
| *YGR231C* | *PHB2* | 2.5 |  |  |  |  |  | 1 |
| *YKL147C* |  |  | -1.6 |  |  |  |  | 1 |
| *YMR008C* | *PLB1* |  | -1.3 |  |  |  |  | 1 |
| *YMR072W* | *ABF2* |  | 1.8 |  |  |  |  | 1 |
| *YFR048W* | *RMD8* |  | 2.3 |  |  |  |  | 1 |
| *YMR021C* | *MAC1* |  |  | -2.7 |  |  |  | 1 |
| *YPL006W* | *NCR1* |  |  | -2.5 |  |  |  | 1 |
| *YKL037W* |  |  |  | -2.2 |  |  |  | 1 |
| *YLR435W* | *TSR2* |  |  | -2.1 |  |  |  | 1 |
| *YML007W* | *YAP1* |  |  | -2.1 |  |  |  | 1 |
| *YLR386W* | *VAC14* |  |  | -2 |  |  |  | 1 |
| *YKL119C* | *VPH2* |  |  | -1.85 |  |  |  | 1 |
| *YHL007C* | *STE20* |  |  | -1.7 |  |  |  | 1 |
| *YGL168W* | *HUR1* |  |  | -1.6 |  |  |  | 1 |
| *YOR068C* | *VAM10* |  |  | -1.4 |  |  |  | 1 |
| *YJR073C* | *OPI3* |  |  | -1.55 |  |  |  | 1 |
| *YOR325W* |  |  |  | -1.3 |  |  |  | 1 |
| *YER020W* | *GPA2* |  |  | -1.2 |  |  |  | 1 |
| *YLR193C* | *UPS1* |  |  | -1.2 |  |  |  | 1 |
| *YHL046C* | *PAU13* |  |  | -1.1 |  |  |  | 1 |
| *YJL070C* |  |  |  | 1.1 |  |  |  | 1 |
| *YDR251W* | *PAM1* |  |  | 1.3 |  |  |  | 1 |
| *YJR053W* | *BFA1* |  |  | 1.3 |  |  |  | 1 |
| *YNL058C* |  |  |  | 1.3 |  |  |  | 1 |
| *YLR257W* |  |  |  | 1.4 |  |  |  | 1 |
| *YML048W-A* |  |  |  | 1.4 |  |  |  | 1 |
| *YOR044W* | *IRC23* |  |  | 1.5 |  |  |  | 1 |
| *YGL214W* |  |  |  | 1.6 |  |  |  | 1 |
| *YPL140C* | *MKK2* |  |  | 1.7 |  |  |  | 1 |
| *YCL046W* |  |  |  | 1.8 |  |  |  | 1 |
| *YLR121C* | *YPS3* |  |  | 1.9 |  |  |  | 1 |
| *YCR006C* |  |  |  | 1.9 |  |  |  | 1 |
| *YLR044C* | *PDC1* |  |  | 2 |  |  |  | 1 |
| *YDR162C* | *NBP2* | -5.5 |  |  |  |  |  | 1 |
| *YDR069C* | *DOA4* | -3.5 |  |  |  |  |  | 1 |
| *YBL093C* | *ROX3* | -3.1 |  |  |  |  |  | 1 |
| *YGR229C* | *SMI1* | -2.9 |  |  |  |  |  | 1 |
| *YOR080W* | *DIA2* | -2.7 |  |  |  |  |  | 1 |
| *YML094W* | *GIM5* | -2.6 |  |  |  |  |  | 1 |
| *YPL125W* | *KAP120* | -2.4 |  |  |  |  |  | 1 |
| *YOR331C* |  | -2.3 |  |  |  |  |  | 1 |
| *YGR034W* | *RPL26B* | 1.9 |  |  |  |  |  | 1 |
| *YIL003W* | *CFD1* | 2.6 |  |  |  |  |  | 1 |
| *YGL020C* | *GET1* | 2.8 |  |  |  |  |  | 1 |
| *YKL135C* | *APL2* | 2.9 |  |  |  |  |  | 1 |
| *YMR166C* |  |  | 2.6 |  |  |  |  | 1 |
| *YKR035C* | *OPI8* |  | 2.9 |  |  |  |  | 1 |
| *YCL007C* |  |  |  | -2.7 |  |  |  | 1 |
| *YDR200C* | *VPS64* |  |  | -2.2 |  |  |  | 1 |
| *YGL004C* | *RPN14* |  |  | 1.7 |  |  |  | 1 |
| *YLL057C* | *JLP1* |  |  | 1.9 |  |  |  | 1 |
| *YKL066W* |  |  |  | 1.9 |  |  |  | 1 |
| *YBL087C* | *RPL23A* |  |  | 2.1 |  |  |  | 1 |
| *YPL035C* |  |  |  | 2.2 |  |  |  | 1 |
| *YGL179C* | *TOS3* |  |  | 3 |  |  |  | 1 |
| *YDR431W* |  |  |  |  | -2.4 |  |  | 1 |
| *YPR166C* | *MRP2* |  |  |  | 1.6 |  |  | 1 |
| *YDR255C* | *RMD5* |  |  |  | 1.8 |  |  | 1 |
| *YDR271C* | *NA* |  |  |  | 2.2 |  |  | 1 |
| *YLR053C* |  |  |  |  | 1.9 |  |  | 1 |
| *YIR024C* |  |  |  |  | 1.9 |  |  | 1 |
| *YNL128W* | *TEP1* |  |  |  | 2.45 |  |  | 1 |
| *YNL338W* |  |  |  |  | 2 |  |  | 1 |
| *YOR138C* | *RUP1* |  |  |  | 2 |  |  | 1 |
| *YHL037C* |  |  |  |  | 2 |  |  | 1 |
| *YGL241W* | *KAP114* |  |  |  | 2 |  |  | 1 |
| *YHR180W* |  |  |  |  | 2 |  |  | 1 |
| *YLR057W* |  |  |  |  | 2 |  |  | 1 |
| *YJL013C* | *MAD3* |  |  |  | 2 |  |  | 1 |
| *YIR025W* | *MND2* |  |  |  | 2 |  |  | 1 |
| *YPL003W* | *ULA1* |  |  |  | 2.25 |  |  | 1 |
| *YKR105C* |  |  |  |  | 2.1 |  |  | 1 |
| *YJR149W* |  |  |  |  | 2.1 |  |  | 1 |
| *YFL006W* |  |  |  |  | 2.1 |  |  | 1 |
| *YLR142W* | *PUT1* |  |  |  | 2.1 |  |  | 1 |
| *YMR137C* | *PSO2* |  |  |  | 2.1 |  |  | 1 |
| *YDR258C* | *HSP78* |  |  |  | 2.1 |  |  | 1 |
| *YNL217W* |  |  |  |  | 2.1 |  |  | 1 |
| *YBR231C* | *SWC5* |  |  |  | 2.1 |  |  | 1 |
| *YCR107W* | *AAD3* |  |  |  | 2.2 |  |  | 1 |
| *YLR190W* | *MMR1* |  |  |  | 2.2 |  |  | 1 |
| *YER088C* | *DOT6* |  |  |  | 2.2 |  |  | 1 |
| *YBR219C* |  |  |  |  | 2.2 |  |  | 1 |
| *YER042W* | *MXR1* |  |  |  | 2.2 |  |  | 1 |
| *YJL168C* | *SET2* |  |  |  | 2.2 |  |  | 1 |
| *YLL061W* | *MMP1* |  |  |  | 2.2 |  |  | 1 |
| *YOR033C* | *EXO1* |  |  |  | 2.2 |  |  | 1 |
| *YLR170C* | *APS1* |  |  |  | 2.2 |  |  | 1 |
| *YDR461W* | *MFA1* |  |  |  | 2.3 |  |  | 1 |
| *YBR210W* | *ERV15* |  |  |  | 2.3 |  |  | 1 |
| *YLR443W* | *ECM7* |  |  |  | 2.3 |  |  | 1 |
| *YOR167C* | *RPS28A* |  |  |  | 2.3 |  |  | 1 |
| *YOR086C* | *TCB1* |  |  |  | 2.3 |  |  | 1 |
| *YFR024C-A* | *LSB3* |  |  |  | 2.3 |  |  | 1 |
| *YMR053C* | *STB2* |  |  |  | 2.3 |  |  | 1 |
| *YLR098C* | *CHA4* |  |  |  | 2.3 |  |  | 1 |
| *YHR176W* | *FMO1* |  |  |  | 2.4 |  |  | 1 |
| *YDR322W* | *MRPL35* |  |  |  | 2.6 |  |  | 1 |
| *YHR095W* |  |  |  |  | 2.4 |  |  | 1 |
| *YIR031C* | *DAL7* |  |  |  | 2.4 |  |  | 1 |
| *YDR193W* |  |  |  |  | 2.4 |  |  | 1 |
| *YHR195W* | *NVJ1* |  |  |  | 2.4 |  |  | 1 |
| *YDR456W* | *NHX1* |  |  |  | 2.4 |  |  | 1 |
| *YNL098C* | *RAS2* |  |  |  | 2.4 |  |  | 1 |
| *YLR049C* |  |  |  |  | 2.4 |  |  | 1 |
| *YHR150W* | *PEX28* |  |  |  | 2.4 |  |  | 1 |
| *YPR106W* | *ISR1* |  |  |  | 2.4 |  |  | 1 |
| *YLR151C* | *PCD1* |  |  |  | 2.5 |  |  | 1 |
| *YJL208C* | *NUC1* |  |  |  | 2.5 |  |  | 1 |
| *YIL006W* | *YIA6* |  |  |  | 2.5 |  |  | 1 |
| *YBR164C* | *ARL1* |  |  |  | 2.5 |  |  | 1 |
| *YHR189W* | *PTH1* |  |  |  | 2.5 |  |  | 1 |
| *YIL092W* |  |  |  |  | 2.6 |  |  | 1 |
| *YIL152W* |  |  |  |  | 2.6 |  |  | 1 |
| *YBR250W* | *SPO23* |  |  |  | 2.6 |  |  | 1 |
| *YNL130C* | *CPT1* |  |  |  | 2.6 |  |  | 1 |
| *YDR269C* |  |  |  |  | 2.6 |  |  | 1 |
| *YMR083W* | *ADH3* |  |  |  | 2.6 |  |  | 1 |
| *YHR147C* | *MRPL6* |  |  |  | 3.05 |  |  | 1 |
| *YKR093W* | *PTR2* |  |  |  | 2.7 |  |  | 1 |
| *YIR020C* |  |  |  |  | 2.7 |  |  | 1 |
| *YNR064C* |  |  |  |  | 2.7 |  |  | 1 |
| *YBR008C* | *FLR1* |  |  |  | 2.8 |  |  | 1 |
| *YGR121C* | *MEP1* |  |  |  | 2.8 |  |  | 1 |
| *YMR002W* | *MIC17* |  |  |  | 2.9 |  |  | 1 |
| *YOL028C* | *YAP7* |  |  |  | 2.9 |  |  | 1 |
| *YGL199C* | *NA* |  |  |  | 2.6 |  |  | 1 |
| *YGR143W* | *SKN1* |  |  |  | 2.8 |  |  | 1 |
| *YOL079W* |  |  |  |  | 3.1 |  |  | 1 |
| *YOR308C* | *SNU66* |  |  |  | 3.1 |  |  | 1 |
| *YPR201W* | *ARR3* |  |  |  | 3.2 |  |  | 1 |
| *YIL052C* | *RPL34B* |  |  |  | 3.3 |  |  | 1 |
| *YOL031C* | *SIL1* |  |  |  | 3.3 |  |  | 1 |
| *YDR305C* | *HNT2* |  |  |  | 2.65 |  |  | 1 |
| *YHR133C* | *NSG1* |  |  |  | 3.4 |  |  | 1 |
| *YIL044C* | *AGE2* |  |  |  | 3.7 |  |  | 1 |
| *YDL222C* | *FMP45* |  |  |  | 3.9 |  |  | 1 |
| *YIL096C* |  |  |  |  | 4.1 |  |  | 1 |
| *YLR417W* | *VPS36* |  |  |  |  | -2.4 |  | 1 |
| *YPR160W* | *GPH1* |  |  |  |  | 1.7 |  | 1 |
| *YPL221W* | *FLC1* |  |  |  |  | 1.8 |  | 1 |
| *YHR185C* | *PFS1* |  |  |  |  | 1.8 |  | 1 |
| *YER080W* | *FMP29* |  |  |  |  | 1.8 |  | 1 |
| *YJR032W* | *CPR7* |  |  |  |  | 2.2 |  | 1 |
| *YCR073W-A* | *SOL2* |  |  |  |  | 2.3 |  | 1 |
| *YGR209C* | *TRX2* |  |  |  |  |  | -4.45 | 1 |
| *YPL170W* | *DAP1* |  |  |  |  |  | -3.5 | 1 |
| *YAL009W* | *SPO7* |  |  |  |  |  | -2.8 | 1 |
| *YIL154C* | *IMP2'* |  |  |  |  |  | -3.2 | 1 |
| *YLR387C* | *REH1* |  |  |  |  |  | -2.6 | 1 |
| *YHR004C* | *NEM1* |  |  |  |  |  | -2.5 | 1 |
| *YKL199C* |  |  |  |  |  |  | -2.5 | 1 |
| *YOL089C* | *HAL9* |  |  |  |  |  | -2.4 | 1 |
| *YGL164C* | *YRB30* |  |  |  |  |  | -2.4 | 1 |
| *YPL147W* | *PXA1* |  |  |  |  |  | -2.3 | 1 |
| *YKR020W* | *VPS51* |  |  |  |  |  | -2.3 | 1 |
| *YKL215C* |  |  |  |  |  |  | -2.2 | 1 |
| *YML053C* |  |  |  |  |  |  | -2 | 1 |
| *YJR019C* | *TES1* |  |  |  |  |  | -1.9 | 1 |
| *YAL005C* | *SSA1* |  |  |  |  |  | -1.9 | 1 |
| *YMR019W* | *STB4* |  |  |  |  |  | -1.7 | 1 |
| *YGL146C* |  |  |  |  |  |  | -1.7 | 1 |
| *YJL004C* | *SYS1* |  |  |  |  |  | -1.7 | 1 |
| *YKL131W* |  |  |  |  |  |  | -1.6 | 1 |
| *YKR052C* | *MRS4* |  |  |  |  |  | -1.5 | 1 |
| *YCR106W* | *RDS1* |  |  |  |  |  | -1.5 | 1 |
| *YDL242W* |  |  |  |  |  |  | -1.4 | 1 |
| *YLL013C* | *PUF3* |  |  |  |  |  | 1.2 | 1 |
| *YMR155W* |  |  |  |  |  |  | 1.3 | 1 |
| *YPR065W* | *ROX1* |  |  |  |  |  | 1.7 | 1 |
| *YER167W* | *BCK2* |  |  |  |  |  | 1.7 | 1 |
| *YDR447C* | *RPS17B* |  |  |  |  |  | 1.8 | 1 |
| *YGR271W* | *SLH1* |  |  |  |  |  | 1.9 | 1 |
| *YDR440W* | *DOT1* |  |  |  |  |  | 1.9 | 1 |
| *YIL060W* |  |  |  |  |  |  | 1.9 | 1 |
| *YML124C* | *TUB3* |  |  |  |  |  | 1.9 | 1 |
| *YBR071W* |  |  |  |  |  |  | 2.2 | 1 |
| *YLR327C* | *TMA10* |  |  |  |  |  | 2.2 | 1 |
| *YJL217W* |  |  |  |  |  |  | 2.3 | 1 |
| *YOR123C* | *LEO1* |  |  |  |  |  | 2.5 | 1 |
| *YGR220C* | *MRPL9* |  |  |  |  |  | 2.4 | 1 |
| *YGR276C* | *RNH70* |  |  |  |  |  | 2.4 | 1 |
| *YOR201C* | *MRM1* |  |  |  |  |  | 2.4 | 1 |
| *YBL038W* | *MRPL16* |  |  |  |  |  | 3.05 | 1 |
| *YDL044C* | *MTF2* |  |  |  |  |  | 2.5 | 1 |
| *YJL052W* | *TDH1* |  |  |  |  |  | 2.6 | 1 |
| *YOR006C* |  |  |  |  |  |  | 2.6 | 1 |
| *YMR193W* | *MRPL24* |  |  |  |  |  | 3.25 | 1 |
| *YGR102C* |  |  |  |  |  |  | 2.45 | 1 |
| *YLL033W* | *IRC19* |  |  |  |  |  | 2.6 | 1 |
| *YFL025C* | *BST1* |  |  |  |  |  | 2.7 | 1 |
| *YMR044W* | *IOC4* |  |  |  |  |  | 2.7 | 1 |
| *YPL118W* | *MRP51* |  |  |  |  |  | 2.7 | 1 |
| *YFL013C* | *IES1* |  |  |  |  |  | 2.8 | 1 |
| *YML061C* | *PIF1* |  |  |  |  |  | 2.8 | 1 |
| *YDR347W* | *MRP1* |  |  |  |  |  | 2.75 | 1 |
| *YNL284C* | *MRPL10* |  |  |  |  |  | 2.65 | 1 |
| *YEL050C* | *RML2* |  |  |  |  |  | 2.85 | 1 |
| *YER077C* |  |  |  |  |  |  | 2.9 | 1 |
| *YML010W-A* |  |  |  |  |  |  | 3 | 1 |
| *YPL005W* | *AEP3* |  |  |  |  |  | 3.05 | 1 |
| *YER087W* |  |  |  |  |  |  | 3 | 1 |
| *YER050C* | *RSM18* |  |  |  |  |  | 2.8 | 1 |
| *YBR251W* | *MRPS5* |  |  |  |  |  | 3.2 | 1 |
| *YMR267W* | *PPA2* |  |  |  |  |  | 3.3 | 1 |
| *YMR097C* | *MTG1* |  |  |  |  |  | 3.2 | 1 |
| *YLR055C* | *SPT8* |  |  |  |  |  | 2.9 | 1 |
| *YIL036W* | *CST6* |  |  |  |  |  | 4.45 | 1 |
| *YNL099C* | *OCA1* |  |  |  | 1.7 |  |  | 1 |
| *YNL154C* | *YCK2* |  |  |  | 1.7 |  |  | 1 |
| *YOL068C* | *HST1* |  |  |  | 1.7 |  |  | 1 |
| *YJL048C* | *UBX6* |  |  |  | 1.8 |  |  | 1 |
| *YBR058C* | *UBP14* |  |  |  | 1.9 |  |  | 1 |
| *YJL150W* | *NA* |  |  |  | 1.9 |  |  | 1 |
| *YGL220W* | *NA* |  |  |  | 1.9 |  |  | 1 |
| *YNL203C* | *NA* |  |  |  | 1.9 |  |  | 1 |
| *YMR306W* | *FKS3* |  |  |  | 1.9 |  |  | 1 |
| *YBR158W* | *AMN1* |  |  |  | 1.9 |  |  | 1 |
| *YNL170W* | *NA* |  |  |  | 1.9 |  |  | 1 |
| *YHR105W* | *YPT35* |  |  |  | 1.9 |  |  | 1 |
| *YBR269C* | *FMP21* |  |  |  | 1.9 |  |  | 1 |
| *YJL082W* | *IML2* |  |  |  | 2 |  |  | 1 |
| *YGR043C* | *NA* |  |  |  | 2 |  |  | 1 |
| *YIL114C* | *POR2* |  |  |  | 2 |  |  | 1 |
| *YPL113C* | *NA* |  |  |  | 2 |  |  | 1 |
| *YGR004W* | *PEX31* |  |  |  | 2 |  |  | 1 |
| *YDR360W* | *OPI7* |  |  |  | 2 |  |  | 1 |
| *YHR108W* | *GGA2* |  |  |  | 2 |  |  | 1 |
| *YGR108W* | *CLB1* |  |  |  | 2.1 |  |  | 1 |
| *YMR084W* | *NA* |  |  |  | 2.1 |  |  | 1 |
| *YDR405W* | *MRP20* |  |  |  | 2.1 |  |  | 1 |
| *YER103W* | *SSA4* |  |  |  | 2.1 |  |  | 1 |
| *YDL138W* | *RGT2* |  |  |  | 2.1 |  |  | 1 |
| *YPR052C* | *NHP6A* |  |  |  | 2.1 |  |  | 1 |
| *YPL183C* | *NA* |  |  |  | 2.1 |  |  | 1 |
| *YCR082W* | *AHC2* |  |  |  | 2.1 |  |  | 1 |
| *YHR182W* | *NA* |  |  |  | 2.1 |  |  | 1 |
| *YBR031W* | *RPL4A* |  |  |  | 2.1 |  |  | 1 |
| *YBR197C* | *NA* |  |  |  | 2.1 |  |  | 1 |
| *YJL214W* | *HXT8* |  |  |  | 2.2 |  |  | 1 |
| *YHR106W* | *TRR2* |  |  |  | 2.2 |  |  | 1 |
| *YGR133W* | *PEX4* |  |  |  | 2.2 |  |  | 1 |
| *YBR162C* | *TOS1* |  |  |  | 2.2 |  |  | 1 |
| *YNL270C* | *ALP1* |  |  |  | 2.3 |  |  | 1 |
| *YLR341W* | *SPO77* |  |  |  | 2.3 |  |  | 1 |
| *YGL090W* | *LIF1* |  |  |  | 2.3 |  |  | 1 |
| *YDR286C* | *NA* |  |  |  | 2.3 |  |  | 1 |
| *YMR101C* | *SRT1* |  |  |  | 2.3 |  |  | 1 |
| *YLR225C* | *NA* |  |  |  | 2.3 |  |  | 1 |
| *YHL002W* | *HSE1* |  |  |  | 2.3 |  |  | 1 |
| *YBR084C-A* | *RPL19A* |  |  |  | 2.4 |  |  | 1 |
| *YPL099C* | *FMP14* |  |  |  | 2.4 |  |  | 1 |
| *YBL010C* | *NA* |  |  |  | 2.4 |  |  | 1 |
| *YDR248C* | *NA* |  |  |  | 2.4 |  |  | 1 |
| *YDR333C* | *NA* |  |  |  | 2.4 |  |  | 1 |
| *YGL261C* | *PAU11* |  |  |  | 2.4 |  |  | 1 |
| *YLR366W* | *NA* |  |  |  | 2.5 |  |  | 1 |
| *YLR004C* | *THI73* |  |  |  | 2.5 |  |  | 1 |
| *YDR370C* | *NA* |  |  |  | 2.5 |  |  | 1 |
| *YDL200C* | *MGT1* |  |  |  | 2.5 |  |  | 1 |
| *YML076C* | *WAR1* |  |  |  | 2.6 |  |  | 1 |
| *YBL013W* | *FMT1* |  |  |  | 2.6 |  |  | 1 |
| *YJL145W* | *SFH5* |  |  |  | 2.7 |  |  | 1 |
| *YJL007C* | *NA* |  |  |  | 2.8 |  |  | 1 |
| *YMR139W* | *RIM11* |  |  |  | 2.8 |  |  | 1 |
| *YGR052W* | *FMP48* |  |  |  | 2.8 |  |  | 1 |
| *YDR144C* | *MKC7* |  |  |  | 2.8 |  |  | 1 |
| *YOR234C* | *RPL33B* |  |  |  | 2.8 |  |  | 1 |
| *YNL141W* | *AAH1* |  |  |  | 2.8 |  |  | 1 |
| *YOR230W* | *WTM1* |  |  |  | 2.9 |  |  | 1 |
| *YOR380W* | *RDR1* |  |  |  | 2.9 |  |  | 1 |
| *YLR346C* | *NA* |  |  |  | 2.9 |  |  | 1 |
| *YBR107C* | *IML3* |  |  |  | 2.9 |  |  | 1 |
| *YGR053C* | *NA* |  |  |  | 2.9 |  |  | 1 |
| *YHR152W* | *SPO12* |  |  |  | 3 |  |  | 1 |
| *YOR275C* | *RIM20* |  |  |  | 3.1 |  |  | 1 |
| *YMR161W* | *HLJ1* |  |  |  | 3.1 |  |  | 1 |
| *YOR386W* | *PHR1* |  |  |  | 3.1 |  |  | 1 |
| *YPL132W* | *COX11* |  |  |  | 3.2 |  |  | 1 |
| *YOR118W* | *NA* |  |  |  | 3.2 |  |  | 1 |
| *YMR195W* | *ICY1* |  |  |  | 3.3 |  |  | 1 |
| *YHR160C* | *PEX18* |  |  |  | 3.3 |  |  | 1 |
| *YDL175C* | *AIR2* |  |  |  | 3.3 |  |  | 1 |
| *YLL021W* | *SPA2* |  |  |  | 3.3 |  |  | 1 |
| *YBR015C* | *MNN2* |  |  |  | 3.3 |  |  | 1 |
| *YNL140C* | *NA* |  |  |  | 3.3 |  |  | 1 |
| *YNL135C* | *FPR1* |  |  |  | 3.4 |  |  | 1 |
| *YHR171W* | *ATG7* |  |  |  | 3.5 |  |  | 1 |
| *YOR045W* | *TOM6* |  |  |  | 3.5 |  |  | 1 |
| *YGL088W* | *NA* |  |  |  | 3.7 |  |  | 1 |
| *YDR340W* | *NA* |  |  |  | 3.8 |  |  | 1 |
| *YDR122W* | *KIN1* |  |  |  | 3.8 |  |  | 1 |
| *YEL010W* | *NA* |  |  |  | 3.8 |  |  | 1 |
| *YBR172C* | *SMY2* |  |  |  | 3.9 |  |  | 1 |
| *YMR164C* | *MSS11* |  |  |  | 4 |  |  | 1 |
| *YBR084W* | *MIS1* |  |  |  | 4.2 |  |  | 1 |
| *YNL177C* | *MRPL22* |  |  |  | 4.2 |  |  | 1 |
| *YHL025W* | *SNF6* |  |  |  |  | -3.7 |  | 1 |
| *YDL020C* | *RPN4* |  |  |  |  | -2.4 |  | 1 |
| *YKL222C* |  |  |  |  |  | -2.4 |  | 1 |
| *YGR110W* |  |  |  |  |  | -2.2 |  | 1 |
| *YNL041C* | *COG6* |  |  |  |  | -2.2 |  | 1 |
| *YER188W* |  |  |  |  |  | -1.8 |  | 1 |
| *YKL161C* |  |  |  |  |  | -1.7 |  | 1 |
| *YDL239C* | *ADY3* |  |  |  |  | -1.6 |  | 1 |
| *YMR153C-A* |  |  |  |  |  | -1.5 |  | 1 |
| *YMR182C* | *RGM1* |  |  |  |  | 2.1 |  | 1 |
| *YMR184W* | *ADD37* |  |  |  |  | 2.3 |  | 1 |
| *YJR130C* | *STR2* |  |  |  |  | 2.4 |  | 1 |
| *YDR388W* | *RVS167* |  |  |  |  | 2.4 |  | 1 |
| *YMR244C-A* |  |  |  |  |  | 2.6 |  | 1 |
| *YMR114C* |  |  |  |  |  | 3 |  | 1 |
| *YJL204C* | *RCY1* |  |  |  |  |  | -3.3 | 1 |
| *YCR094W* | *CDC50* |  |  |  |  |  | -3.1 | 1 |
| *YHR045W* |  |  |  |  |  |  | -3 | 1 |
| *YLR111W* |  |  |  |  |  |  | -2.6 | 1 |
| *YBR298C* | *MAL31* |  |  |  |  |  | -2.6 | 1 |
| *YCL001W-A* |  |  |  |  |  |  | -2.5 | 1 |
| *YEL065W* | *SIT1* |  |  |  |  |  | -2.1 | 1 |
| *YJR040W* | *GEF1* |  |  |  |  |  | -2.1 | 1 |
| *YOL002C* | *IZH2* |  |  |  |  |  | -1.8 | 1 |
| *YDR338C* |  |  |  |  |  |  | 1.5 | 1 |
| *YIL107C* | *PFK26* |  |  |  |  |  | 1.5 | 1 |
| *YDR424C* | *DYN2* |  |  |  |  |  | 1.6 | 1 |
| *YBR129C* | *OPY1* |  |  |  |  |  | 1.6 | 1 |
| *YLR334C* |  |  |  |  |  |  | 1.7 | 1 |
| *YGL016W* | *KAP122* |  |  |  |  |  | 1.8 | 1 |
| *YMR284W* | *YKU70* |  |  |  |  |  | 2 | 1 |
| *YMR188C* | *MRPS17* |  |  |  |  |  | 2 | 1 |
| *YIL112W* | *HOS4* |  |  |  |  |  | 2.1 | 1 |
| *YDR423C* | *CAD1* |  |  |  |  |  | 2.1 | 1 |
| *YBL079W* | *NUP170* |  |  |  |  |  | 2.2 | 1 |
| *YHR111W* | *UBA4* |  |  |  |  |  | 2.2 | 1 |
| *YPL182C* |  |  |  |  |  |  | 2.3 | 1 |
| *YBL071C* |  |  |  |  |  |  | 2.4 | 1 |
| *YCL005W* | *LDB16* |  |  |  |  |  | 2.5 | 1 |
| *YER122C* | *GLO3* |  |  |  |  |  | 2.5 | 1 |
| *YDR384C* | *ATO3* |  |  |  |  |  | 2.5 | 1 |
| *YGL064C* | *MRH4* |  |  |  |  |  | 2.6 | 1 |
| *YLR069C* | *MEF1* |  |  |  |  |  | 2.6 | 1 |
| *YOR205C* | *FMP38* |  |  |  |  |  | 2.7 | 1 |
| *YFL036W* | *RPO41* |  |  |  |  |  | 2.7 | 1 |
| *YGR215W* | *RSM27* |  |  |  |  |  | 2.7 | 1 |
| *YMR228W* | *MTF1* |  |  |  |  |  | 2.7 | 1 |
| *YPL102C* |  |  |  |  |  |  | 2.8 | 1 |
| *YDR268W* | *MSW1* |  |  |  |  |  | 2.9 | 1 |
| *YLR382C* | *NAM2* |  |  |  |  |  | 2.9 | 1 |
| *YOL023W* | *IFM1* |  |  |  |  |  | 3 | 1 |
| *YNL120C* |  |  |  |  |  |  | 3 | 1 |
| *YMR098C* |  |  |  |  |  |  | 3.1 | 1 |
| *YDR237W* | *MRPL7* |  |  |  |  |  | 3.2 | 1 |
| *YNL119W* | *NCS2* |  |  |  |  |  | 3.2 | 1 |
| *YLR402W* |  |  |  |  |  |  | 3.3 | 1 |
| *YGL240W* | *DOC1* |  |  |  |  |  | 3.3 | 1 |
| *YDR298C* | *ATP5* |  |  |  |  |  | 3.4 | 1 |
| *YIL009C-A* | *EST3* |  |  |  |  |  | 3.5 | 1 |
| *YDR359C* | *VID21* |  |  |  |  |  | 3.7 | 1 |
| *YBL090W* | *MRP21* |  |  |  |  |  | 3.8 | 1 |
